# Theoretical Study of the Photochemical Mechanisms of the Electronic Quenching of NO(*A*^2^Σ^+^) with CH_4_, CH_3_OH, and CO_2_

**DOI:** 10.1021/acs.jpca.3c03981

**Published:** 2023-08-08

**Authors:** Aerial N. Bridgers, Justin A. Urquilla, Julia Im, Andrew S. Petit

**Affiliations:** Department of Chemistry and Biochemistry, California State University—Fullerton, Fullerton, California 92834-6866, United States

## Abstract

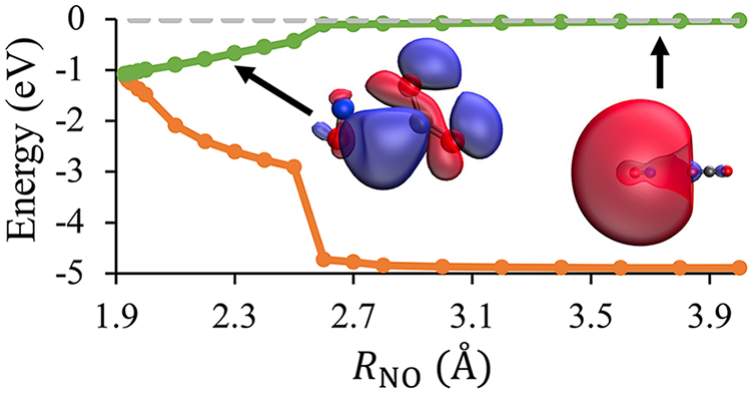

The electronic quenching of NO(*A*^2^Σ^+^) with molecular partners occurs through complex non-adiabatic dynamics that occurs on multiple coupled potential energy surfaces. Moreover, the propensity for NO(*A*^2^Σ^+^) electronic quenching depends heavily on the strength and nature of the intermolecular interactions between NO(*A*^2^Σ^+^) and the molecular partner. In this paper, we explore the electronic quenching mechanisms of three systems: NO(*A*^2^Σ^+^) + CH_4_, NO(*A*^2^Σ^+^) + CH_3_OH, and NO(*A*^2^Σ^+^) + CO_2_. Using EOM-EA-CCSD calculations, we rationalize the very low electronic quenching cross-section of NO(*A*^2^Σ^+^) + CH_4_ as well as the outcomes observed in previous NO + CH_4_ photodissociation studies. Our analysis of NO(*A*^2^Σ^+^) + CH_3_OH suggests that it will undergo facile electronic quenching mediated by reducing the intermolecular distance and significantly stretching the O–H bond of CH_3_OH. For NO(*A*^2^Σ^+^) + CO_2_, intermolecular attractions lead to a series of low-energy ON–OCO conformations in which the CO_2_ is significantly bent. For both the NO(*A*^2^Σ^+^) + CH_3_OH and NO(*A*^2^Σ^+^) + CO_2_ systems, we see evidence of the harpoon mechanism and low-energy conical intersections between NO(*A*^2^Σ^+^) + M and NO(*X*^2^Π) + M. Overall, this work provides the first detailed theoretical study on the NO(*A*^2^Σ^+^) + M potential energy surface of each of these systems and will inform future velocity map imaging experiments.

## Introduction

Nitric oxide (NO) is an atmospherically important free radical generated in the combustion of fossil fuels and biomass. NO is often quantified using laser-induced fluorescence (LIF) on the *A*^2^Σ^+^ ← *X*^2^Π transition band.^1−4^ However, electronic quenching can interfere with the ability of LIF to provide an accurate quantification of NO. Electronic quenching occurs when a collision between NO(*A*^2^Σ^+^) and an atomic or molecular partner causes nonradiative relaxation back to the electronic ground state, NO(*X*^2^Π). Such pathways compete with fluorescence and cause the concentration of NO quantified through LIF to appear lower than the true concentration.^5−7^

Electronic quenching pathways can be separated into two general classes: reactive and nonreactive. In nonreactive electronic quenching, which is illustrated in eq 1, a collision between NO(*A*^2^Σ^+^) and a molecular partner induces nonradiative relaxation down to NO(*X*^2^Π). The energy released by this nonradiative NO(*A*^2^Σ^+^) → NO(*X*^2^Π) electronic transition is divided between the translational, rotational, and vibrational degrees of freedom of the two molecules. As a result, electronic quenching can cause a change in the vibrational and rotational states of both molecules. Equations 2a–2c illustrate different possible reactive electronic quenching pathways for NO(*A*^2^Σ^+^) and CO_2_ proposed in the literature.^8,9^ In all these, the collision between NO(*A*^2^Σ^+^) and CO_2_ causes a chemical reaction.
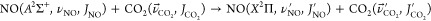
1
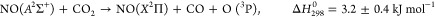
2a
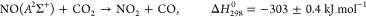
2b
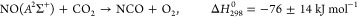
2c

To correct inaccuracies in the quantification of NO through LIF, the electronic quenching cross-sections of NO(*A*^2^Σ^+^) with various molecular partners have been experimentally determined.^6,7,9−11^Table 1 summarizes the electronic quenching cross-section of NO(*A*^2^Σ^+^) with four representative molecular partners. As seen in the table, H_2_O has the largest electronic quenching cross section, indicating that it is the most effective of these molecules at quenching NO(*A*^2^Σ^+^). The electronic quenching cross section for NO(*A*^2^Σ^+^) + CO_2_ is also quite large at 68.3 Å^2^ at 294 K. In contrast, NO(*A*^2^Σ^+^) + CO has an electronic quenching cross section around 5% that of NO(*A*^2^Σ^+^) + H_2_O while NO(*A*^2^Σ^+^) + CH_4_ does not undergo appreciable electronic quenching at 296 K. At lower temperatures, NO(*A*^2^Σ^+^) + H_2_O, NO(*A*^2^Σ^+^) + CO_2_, and NO(*A*^2^Σ^+^) + CO have larger electronic quenching cross sections, consistent with collision complexes playing a role in facilitating the quenching. Though these experimental electronic quenching cross sections are helpful for correcting LIF measurements, they do not reveal the photochemical pathways responsible for electronic quenching.

**Table 1:** Experimentally Determined Electronic Quenching Cross Sections of NO(*A*^2^Σ^+^) with Molecular Partners CH_4_, CO, CO_2_, and H_2_O near Room Temperature^9,12^

molecular partner	electronic quenching cross section (Å^2^)	temperature (K)
CH_4_	<0.001	296
CO	6.55	294
CO_2_	68.3	294
H_2_O	121	294

While the photochemical mechanisms responsible for the electronic quenching of NO(*A*^2^Σ^+^) with atomic partners have been well studied, the non-adiabatic dynamics associated with the electronic quenching of NO(*A*^2^Σ^+^) with molecular partners remain relatively unexplored.^13−17^ Three recent studies of the NO(*A*^2^Σ^+^) + O_2_ system provide a notable exception to this.^18−20^ In the first of these, Few and co-workers used time-resolved Fourier-transform infrared emission spectroscopy to probe the pathways for nonreactive electronic quenching.^18^ Their analysis revealed a bimodal distribution of NO(*X*^2^Π, ν_NO_^′^ = 2–22) products, suggesting the presence of two nonreactive electronic quenching channels. They attributed the formation of NO(*X*^2^Π) with high vibrational excitation to a channel with an O_2_(*X*^3^Σ_g_^–^) or O_2_(*a*^1^Δ_g_) co-product. Few and co-workers speculated that the low ν_NO_^′^ NO(*X*^2^Π) were generated in a second nonreactive electronic quenching channel which involves either the formation of O_2_(*c*^1^Σ_u_^–^) via a harpoon mechanism or the generation of O_2_(*X*^3^Σ_*g*_^–^) through an inefficient process. Recent work by Blackshaw and co-workers used velocity map imaging (VMI) to probe, with quantum state resolution, the formation of NO(*X*^2^Π, ν_NO_^′^ = 0) through NO(*A*^2^Σ^+^) + O_2_ electronic quenching.^19^ By analyzing the total kinetic energy release (TKER), they concluded that O_2_ receives a significant fraction of the available energy. Phase space theory simulations of the TKER distributions strongly suggested that the co-product of the nonreactive electronic quenching is O_2_(*c*^1^Σ_u_^–^), consistent with most of the available energy inducing electronic excitation of the O_2_. Recent theoretical work by Soulié and Paterson, performed using the multireference methods SA-CASSCF and XMS-CASSCF, developed cuts of the NO + O_2_ potential energy surfaces (PESs) to rationalize the experimental observations on this system.^20,21^ They argued that their PESs are consistent with two nonradiative relaxation channels for NO(*A*^2^Σ^+^) + O_2_. The first proceeds through a transient ion-pair generated by electron transfer from NO(*A*^2^Σ^+^) to O_2_. This pathway exhibited a strong dependence on the intermolecular orientation and likely results in significant vibrational excitation in the products due to the large molecular geometry changes caused by the transient electron transfer. The second channel proceeds via conical intersections accessed when the O_2_ bond length becomes significantly elongated. Collectively, the previous studies on NO(*A*^2^Σ^+^) + O_2_ illustrate the complex chemical physics that can be associated with NO(*A*^2^Σ^+^) electronic quenching.

**Figure 1:**
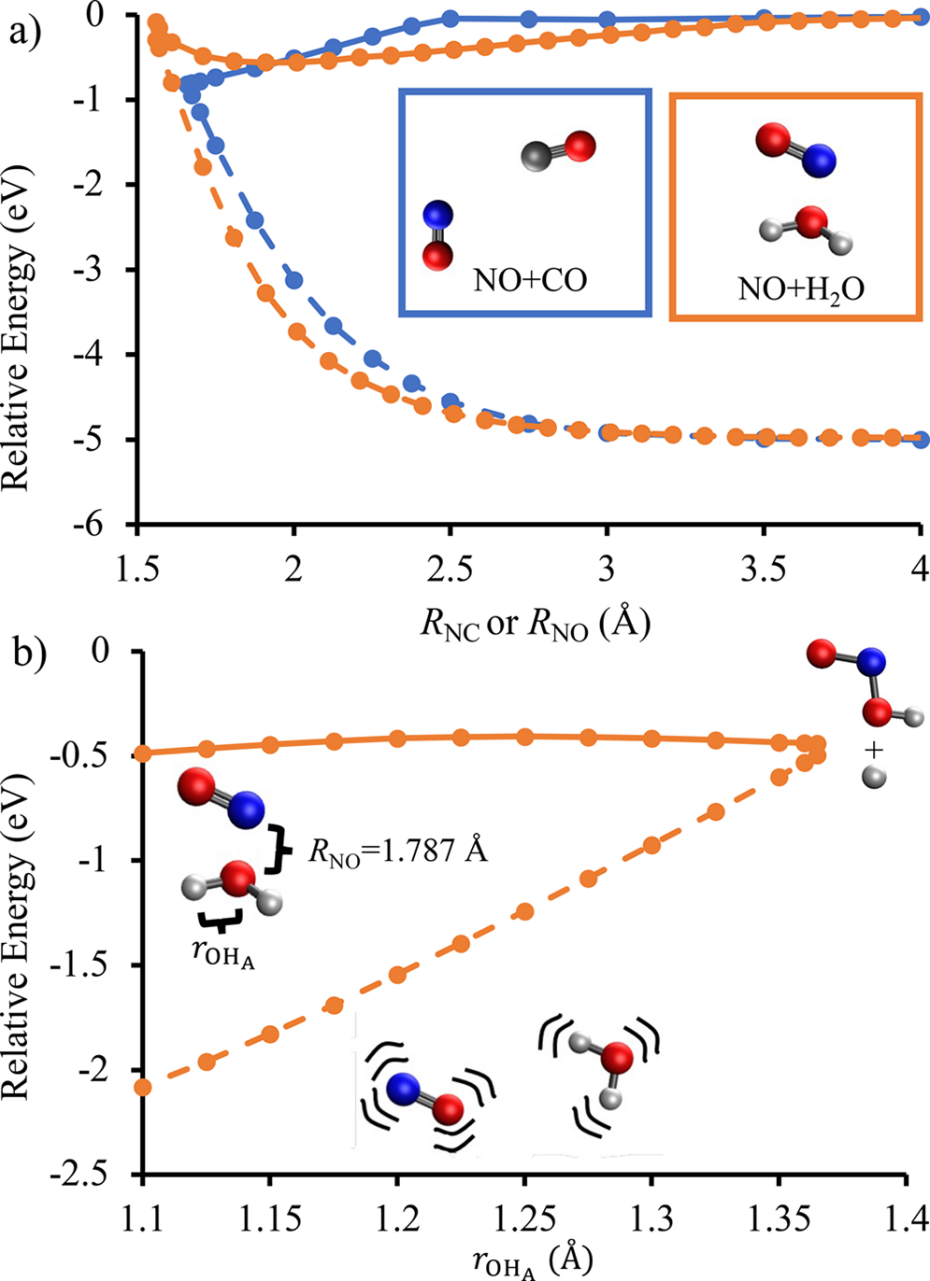
Panel (a) shows the relative energies of the D_1_ (dashed lines) and D_2_ (solid lines) states of the NO + H_2_O (orange) and NO + CO (blue) systems as a function of the intermolecular distance. Panel (b) shows the relative energies of the D_1_ and D_2_ states of NO + H_2_O as a function of one of the OH bond lengths of water, *r*_OH_A__, at a fixed intermolecular distance of *R*_ON_ = 1.787 Å. All energies are reported relative to an optimized geometry on D_2_ with an intermolecular distance of 10 Å. Adapted with permission from the American Chemical Society, ref (22), and the Royal Society of Chemistry, ref (23).

As summarized in Figure 1, our two recent computational studies provide insights into NO(*A*^2^Σ^+^) + H_2_O and NO(*A*^2^Σ^+^) + CO electronic quenching mechanisms.^22,23^ Here, we introduce the notation D_2_ to represent the NO(*A*^2^Σ^+^) + M electronic state, while D_0_ and D_1_ are associated with NO(*X*^2^Π) + M. Note that NO(*X*^2^Π) + M is doubly-degenerate at very large intermolecular distances but splits into two low-lying electronic states due to intermolecular interactions with the molecular partner. Figure 1a shows that the NO + H_2_O and NO + CO intermolecular interactions are attractive for the D_2_ state and strongly repulsive for D_1_. In our previous work, we demonstrated that the attractive intermolecular interactions on D_2_ can be rationalized using the harpoon mechanism. Specifically, electron density shifts from the NO(*A*^2^Σ^+^) 3*s*σ Rydberg orbital to the intermolecular partner’s lowest unoccupied molecular orbital. This creates a transient ion-pair that experiences attractive Coulombic intermolecular interactions. Figure 1 further shows that both NO + H_2_O and NO + CO possess D_1_–D_2_ conical intersections that are energetically downhill in energy from the asymptotic limit. These D_2_–D_1_ conical intersections facilitate the NO(*A*^2^Σ^+^) + H_2_O and NO(*A*^2^Σ^+^) + CO electronic quenching.

Figure 1 also highlights significant differences between NO + H_2_O and NO + CO, which allow for a mechanistic rationalization of the large difference between the electronic quenching cross-sections of NO(*A*^2^Σ^+^) + H_2_O and NO(*A*^2^Σ^+^) + CO. NO + H_2_O has significantly stronger attractive intermolecular interactions on D_2_ than NO + CO. Moreover, the D_2_ PES of NO + H_2_O funnels a wide variety of initial intermolecular orientations to the same low-energy conformation shown in the inset of Figure 1a. In contrast, the NO + CO D_2_ PES is much more anisotropic, with only a narrow range of initial intermolecular orientations producing attractive intermolecular interactions that lead to a D_2_–D_1_ conical intersection. Figure 1b shows that the pathway to the D_2_–D_1_ conical intersection for NO + H_2_O involves the significant stretching of one of the O–H bonds of H_2_O. Nonreactive electronic quenching will produce H_2_O with substantial vibrational excitation in an O–H local mode. Alternatively, a reactive electronic quenching channel is available, which will produce HONO and a H-atom, consistent with previous experimental observations made by Umemoto and co-workers.^24^ Only nonreactive electronic quenching is observed at low collision energies for NO(*A*^2^Σ^+^) + CO.

In this computational study, three different systems are considered: NO(*A*^2^Σ^+^) + CH_4_, NO(*A*^2^Σ^+^)+CH_3_OH, and NO(*A*^2^Σ^+^)+CO_2_. In choosing these systems, we sought to explore how the electronic structure of the molecular partner affects the photochemistry of NO(*A*^2^Σ^+^). CH_4_ is a larger polyatomic molecule than H_2_O with no low-lying π molecular orbitals (MOs). CO_2_ represents a polyatomic extension of our previously studied CO, with multiple heavy atoms and frontier MOs with π-symmetry. CH_3_OH represents a hybrid between methane, which has a very small electronic quenching cross section, and H_2_O, which has a very large electronic quenching cross section. Before outlining the goals of our work, we summarize previous experimental and theoretical work on the NO + CH_4_ and NO + CO_2_ systems; to the best of our knowledge, the NO + CH_3_OH system has not been previously studied.

Several previous studies have computationally and spectroscopically explored the NO(*X*^2^Π) + CH_4_ complex.^25−28^ Crespo-Otero et al. characterized the NO(*X*^2^Π) + CH_4_ PESs at the RCCSD(T)/aug-cc-pVTZ level of theory.^25^ This study identified multiple complexes stabilized by intermolecular interactions between NO and a C–H bond or NO and a CH_3_ face. The NO(*X*^2^Π) + CH_4_ complexes were found to be significantly impacted by the Jahn–Teller effect, with the interaction potential driving geometric distortions away from more symmetric structures. As such, C_s_ geometries were generally more stable than higher-symmetry C_3v_ geometries. The lowest-energy NO(*X*^2^Π) + CH_4_ complex identified in this study has the NO interacting with a CH_3_ face and oriented nearly perpendicular to the intermolecular bond. Experimentally, the earliest work used REMPI on the *A* ← *X* band to probe the structure of NO(*X*^2^Π) + CH_4_. Such spectra were interpreted by Musgrave et al. to reveal a vibrational progression in the NO bending mode that originally appeared to suggest effective C_3v_ structures for both NO(*X*^2^Π) + CH_4_ and NO(*A*^2^Σ^+^) + CH_4_.^26^ The direct observation of NO(*X*^2^Π) + CH_4_ rovibrational transitions was accomplished by Wen and Meyer using near IR-REMPI double resonance spectroscopy.^27^ Careful analysis of their spectra revealed that NO(*X*^2^Π) + CH_4_ is best characterized as a Jahn–Teller distorted system with the NO preferentially oriented perpendicular to the intermolecular bond and undergoing large-amplitude vibrational motions. Recently, Kidwell and co-workers used VMI to probe the product state distributions associated with the infrared photodissociation of the NO(*X*^2^Π) + CH_4_ complex.^28^ They rationalized the observed product state distributions through a careful analysis of symmetry-restrictions and a pair of Jahn–Teller NO(*X*^2^Π) + CH_4_ PESs (D_0_ and D_1_).

The dissociation dynamics of the NO(*A*^2^Σ^+^) + CH_4_ complex have been experimentally interrogated in a series of studies by Lawrance and co-workers.^29,30^ These experiments begin by electronically exciting to above the dissociation threshold of NO(*A*^2^Σ^+^) + CH_4_. The NO(*A*^2^Σ^+^) generated by the fragmentation of the complex was then probed using REMPI. Changing the photon energy used in the REMPI scheme allows one to selectively analyze different ro-vibrational states of NO(*A*^2^Σ^+^). Using VMI detection and energy conservation, Lawrance and co-workers obtained correlated product state distributions for the NO(*A*^2^Σ^+^) and CH_4_ products. The NO(*A*^2^Σ^+^) is produced in a broad distribution of rotational states which span the entire energetically accessible range. In contrast, the dissociation of NO(*A*^2^Σ^+^) + CH_4_ strongly favors small changes to the rotational angular momentum of CH_4_, with the dominant product channels having Δ*J* = 0 or Δ*J* = 1 for CH_4_. Consistent with several previous studies, Lawrance and co-workers showed that NO + CH_4_ is more strongly bound in the excited state than in the ground state. Specifically, the most recently measured NO(*X*^2^Π) + CH_4_ and NO(*A*^2^Σ^+^) + CH_4_ binding energies are 108 ± 2 and 203 ± 2 cm^–1^, respectively.^30^ Finally, note that while the NO(*A*^2^Σ^+^) + CH_4_ complex has been studied experimentally, it has not, to the best of our knowledge, been characterized computationally.

Turning to the NO(*A*^2^Σ^+^) + CO_2_ system, several experimental studies attempted to characterize the products of the reactive electronic quenching channel(s).^7,8,31,32^ Early work by Cohen and Heicklen used gas chromatography to identify CO as the major product of the reactive electronic quenching, which they attributed to eq 2b.^31^ More recent work by Azcárate et al. utilized infrared spectroscopy to determine that 26% of NO(*A*^2^Σ^+^) + CO_2_ electronic quenching produces CO and NO_2_.^32^ Settersten and co-workers used LIF to monitor the kinetics associated with the regeneration of NO(*X*^2^Π, ν_NO_^′^ = 0) through NO(*A*^2^Σ^+^) + CO_2_ electronic quenching.^7^ They found that approximately 60% of the electronic quenching results in the formation of NO(*X*^2^Π) in its vibrational ground state. Note that this population represents a portion of that following the nonreactive electronic quenching channel (eq 1) along with all the population following the reactive quenching channel given by eq 2a; the endothermicity of eq 2a ensures that NO(*X*^2^Π) will only be formed in its ground vibrational state. Interestingly, the formation of NO(*X*^2^Π, ν_NO_^′^ = 0) accounts for only approximately 30% of the electronic quenching population for NO + CO, NO + O_2_, and NO + H_2_O.^7^

The most complete experimental characterization of the NO(*A*^2^Σ^+^) + CO_2_ electronic quenching pathways was performed by Hancock and co-workers.^8^ Here, the reactive and nonreactive electronic quenching product distributions were measured using Fourier transform infrared emission spectroscopy. Focusing first on nonreactive electronic quenching, they found that CO_2_ is produced in a wide range of vibrational states, with approximately 62% of the available energy ending up in the vibrational degrees of freedom of CO_2_. While NO(*X*^2^Π) is also observed in a broad distribution of vibrational states, approximately 80% of the electronic quenching results in the formation of NO(*X*^2^Π) with ν_NO_^′^ = 0 or ν_NO_^′^ = 1, with the major product being NO(*X*^2^Π) in its vibrational ground state. Hancock and co-workers attribute approximately 6% of this to nonreactive quenching (eq 1) based on an extrapolation of the distribution of NO(*X*^2^Π, ν_NO_^′^ ≥ 2) population determined using infrared emission. The remaining 74% is ascribed to reactive quenching via eq 2a, along with additional nonreactive electronic quenching that exceeds that predicted by their extrapolation.

Hancock and co-workers further argue that the other reactive quenching pathways (eqs 2b and 2c) are inconsistent with the observed infrared emission spectra. Specifically, they rule out the second reactive electronic quenching pathway (eq 2b) due to the absence of vibrationally hot NO_2_ and CO in the emission spectra. Because this reaction is highly exothermic, it should produce vibrationally excited NO_2_ and CO. The formation of NO_2_ observed in previous studies is instead ascribed to the reaction between O atoms generated via eq 2a and NO. Turning to eq 2c, the generated NCO is known to undergo reactions with NO. The infrared emission spectra associated with these reactions have previously been measured and are distinct from that measured by Hancock and co-workers.

In this study, we apply similar computational methodologies to those employed previously in the studies summarized in Figure 1. We identify the most important regions of the D_2_ PES for NO + CH_4_, NO + CH_3_OH, and NO + CO_2_ and develop a mechanistic analysis of the photochemical pathways responsible for electronic quenching in each of these systems. In doing so, we explore the viability of both nonreactive and reactive electronic quenching pathways. Throughout, we attempt to develop rationalizations for the experimentally known electronic quenching cross-sections and product state distributions. We additionally explore the extent to which the harpoon mechanism explains the long-range intermolecular attractive interactions in these systems. Finally, our work makes clear predictions about the NO(*A*^2^Σ^+^) + M photochemistry of all three systems which will inform future experimental studies.

## Methods

As in our previous studies, we employ the equation-of-motion electron attachment coupled-cluster singles and doubles (EOM-EA-CCSD) methodology in this work.^22,23^ This allows us to use the closed-shell NO^+^ + M reference to avoid the challenges associated with using an open-shell reference. EOM-EA-CCASD will accurately describe all target doublet states of NO + M whose dominant electron configurations can be generated by adding an electron to a virtual orbital of the NO^+^ + M reference. This is true regardless of whether a NO + M electronic excited state has valence, Rydberg, or charge-transfer character.^33−35^ The EOM-EA-CCSD methodology provides dynamic electron correlation to the target states by incorporating electronic configurations that are doubly excited (and higher) from the NO^+^ + M reference. EOM-EA-CCSD will have much lower accuracy for target states with significant double excitation character; such states would have dominant electronic configurations consistent with exciting an electron in the NO^+^ + M reference and adding an electron to a virtual orbital.^33,34^ All states analyzed in this study were verified to consistently have dominant single excitation character; singly excited determinants accounted for over 93% of the D_0_, D_1_, and D_2_ states for each geometry analyzed in this study.

Throughout this study, we employed a protocol designed to effectively balance accuracy and computational cost. The basis sets used for the geometry optimizations ranged from aug-cc-pVDZ to d-aug-cc-pVTZ and are clearly identified in the text. Larger basis sets were required for the geometry optimizations when the intermolecular interactions were especially weak and while mapping out pathways near D_2_–D_1_ conical intersections. For NO–CO_2_, we performed the single-point calculations using the d-aug-cc-pVQZ basis set for the N and O atoms of NO and the aug-cc-pVQZ basis set for the C and O atoms of CO_2_; we refer to this basis set below as AVQZ. Because NO(*A*^2^Σ^+^) + M has significant Rydberg character, using a doubly augmented basis set is necessary to achieve accurate energetics. As described in the text, we did assess the impact of using the smaller d-aug-cc-pVTZ basis set for the single-point calculations and verified that doing so does not significantly affect the overall mechanistic picture; see Figures S24–S34 in the Supporting Information. Similar results were observed in our previous study of NO(*A*^2^Σ^+^) + CO.^22^ As a result, we used EOM-EA-CCSD/d-aug-cc-pVTZ to describe the energetics of NO(*A*^2^Σ^+^) + CH_3_OH. Löwdin spin densities and partial charges were investigated to determine the electronic properties of the D_2_ and D_1_ states. This analysis was performed at the EOM-EA-CCSD/aug-cc-pVTZ level of theory. All calculations were performed using Q-Chem 6.0 and analyzed using IQmol 3.0.1.^36^ For NO + CH_3_OH, we also analyzed natural transition orbitals using wxMacMolPlt.^37,38^

The weak intermolecular interactions associated with NO(*A*^2^Σ^+^) + CH_4_ necessitated a more careful treatment of basis set superposition and basis set incompleteness errors. As such, we performed a three-point extrapolation to the complete basis set (CBS) limit using the formula
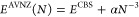
3where *E*^CBS^ is the extrapolated energy in the CBS limit and α is a fitting parameter.^39^ Our three-point extrapolations were based on *N* = 2, 3, and 4 with AVNZ taken to be d-aug-cc-pVNZ for the N and O atoms and aug-cc-pVNZ for C and H atoms. Figure S1 shows an example of our three-point extrapolation to the CBS limit. For all geometries analyzed, the *R*^2^ of the linear regression was always greater than or equal to 0.999.

## Results and Discussion

### NO + CH_4_

**Figure 2:**
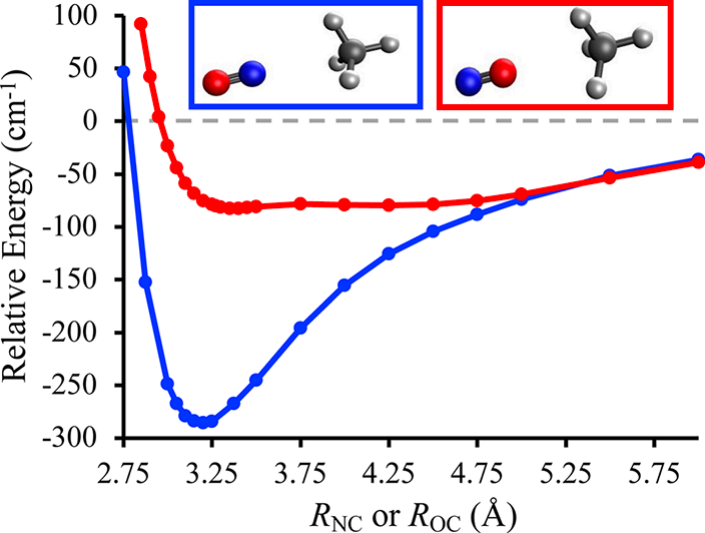
Energy of the D_2_ state of the ON-H_3_CH (blue data) and NO-H_3_CH (red data) conformations plotted against the intermolecular distance of the two interacting atoms. The geometry optimizations were calculated using EOM-EA-CCSD/aug-cc-pVTZ and a three-point extrapolation to the CBS limit was performed for the electronic energies. All energies are reported relative to a D_2_-optimized geometry with an intermolecular distance of 20 Å.

For the NO(*A*^2^Σ^+^) + CH_4_ system, we constructed a wide variety of initial intermolecular orientations and allowed each to optimize on D_2_ using EOM-EA-CCSD/aug-cc-pVTZ without any constraints. Each initial conformation relaxed into one of four molecular geometries. Two of these, which are depicted in the inset of Figure 2, have the NO interacting with one of the CH_3_ faces of CH_4_, while the other two have the NO interacting with a C–H bond. All four stationary points exhibited C_3v_ symmetry despite the geometry optimizations beginning from C_1_ initial geometries.

In order to obtain accurate molecular geometries and interaction energies for the four stationary points, we re-optimized them using EOM-EA-CCSD with the d-aug-cc-pVTZ basis set for the N and O atoms and the aug-cc-pVTZ basis set for the C and H atoms. Harmonic vibrational frequency analysis demonstrated that the ON–H_3_CH and NO–H_3_CH complexes are true minima, while the ON–HCH_3_ and NO–HCH_3_ complexes are saddle points. For the saddle points, the vibrational modes with imaginary frequencies correspond to motions toward the ON–H_3_CH and NO–H_3_CH geometries. The minimum energy geometry of the ON–H_3_CH complex has an intermolecular distance of *R*_NC_ = 3.17 Å and an electronic binding energy of 295.7 cm^–1^ in the CBS limit. This is in reasonable agreement with the NO(*A*^2^Σ^+^) + CH_4_ binding energy determined by recent velocity map imaging experiments, 203 ± 2 cm^–1^.^30^ Better agreement with experiment would require the incorporation of anharmonic zero-point energy. Turning to the other minimum energy geometry, the NO–H_3_CH complex has an intermolecular distance of *R*_OC_ = 3.25 Å and an electronic binding energy of 82.1 cm^–1^.

Figure 2 shows the results of relaxed scans on D_2_ for the ON–H_3_CH and NO–H_3_CH complexes along the intermolecular distance, *R*_NC_ or *R*_OC_. We performed a three-point extrapolation to the CBS limit at every geometry in these scans and the energies are reported relative to the energy of the D_2_ state when the molecules are 20 Å apart. The relative energies are negative for both potential energy curves at larger values of *R*, indicating attractive intermolecular interactions. Consistent with the analysis described above, Figure 2 shows that the ON–H_3_CH complex has a significantly deeper well than the NO–H_3_CH complex. Both potential energy curves become strongly repulsive at closer intermolecular distances. The molecular geometries remained in the C_3v_ point group throughout the attractive region of the PES and only dropped to lower symmetry in the repulsive region.

We now compare the D_2_ PES with those of D_0_ and D_1_. Previous computational work on NO(*X*^2^Π) + CH_4_ revealed eight different local minima, encompassing ON–H_3_CH, NO–H_3_CH, ON–HCH_3_, and NO–HCH_3_ complexes with both C_3v_ and C_s_ symmetry.^25^ In contrast, NO(*A*^2^Σ^+^) preferentially interacts with a CH_3_ face of CH_4_; we identified no minimum-energy geometries where NO(*A*^2^Σ^+^) interacts with a C–H bond. Both theory and experiment agree that NO(*X*^2^Π) + CH_4_ undergoes Jahn–Teller distortion away from C_3v_ structures, with the lowest energy conformations having C_s_ symmetry with the NO oriented perpendicular to the intermolecular bond.^25,27^ In contrast, we find no evidence of Jahn–Teller distortions in the attractive region of the D_2_ PES; all geometries in the attractive region of Figure 2 belonged to the C_3v_ point group. Repeating representative constrained geometry optimizations from molecular geometries distorted into the C_1_ point group resulted in the same C_3v_ structures. Moreover, Figure S2 in the Supporting Information shows that the D_2_ energy increases significantly as the O–N–C angle of ON–H_3_CH is decreased from 180° (C_3v_) to 90° (C_s_). Figure S3 further shows the same behavior for the NO–H_3_CH complex. We posit that the preference for higher-symmetry structures on D_2_ originates from the unpaired electron of NO residing in the more symmetric 3*s*σ MO instead of one of the 2*p*π* MOs.

Our observation that the NO(*A*^2^Σ^+^) + CH_4_ PES supports only C_3v_ minimum energy structures is consistent with the recent experimental work reported by Lawrance and co-workers in which velocity map imaging was used to obtain correlated product state distributions for the photodissociation of NO(*A*^2^Σ^+^) + CH_4_.^30^ Upon NO(*X*^2^Π) + CH_4_ → NO(*A*^2^Σ^+^) + CH_4_ electronic excitation, the NO will re-orient itself from being perpendicular with the intermolecular bond (C_s_ symmetry) to being oriented head-on with the CH_3_ face (C_3v_ symmetry). As such, the vibrational relaxation on NO(*A*^2^Σ^+^) + CH_4_ will include hindered rotation of the NO resulting from a torque imposed on the NO by the D_2_ PES. This supports the experimental observation that the photodissociation of NO(*A*^2^Σ^+^) + CH_4_ produces NO(*A*^2^Σ^+^) in a broad range of final rotational states, including the highest rotational state that was energetically allowed under the experimental conditions. In contrast, the NO(*A*^2^Σ^+^) + CH_4_ photodissociation imparts little rotational energy to the CH_4_.

**Figure 3:**
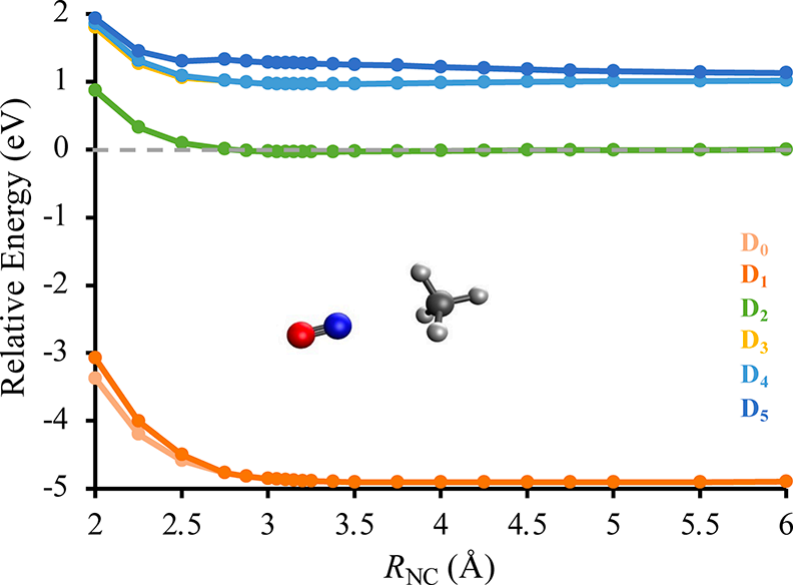
Potential energy curves of the ON–H_3_CH complex showing how the relative energy depends on the intermolecular distance, *R*_NC_. The calculations were performed at the EOM-EA-CCSD/AVQZ//EOM-EA-CCSD/aug-cc-pVTZ level of theory, and all energies are reported relative to a D_2_-optimized geometry with an intermolecular distance of 20 Å. The AVQZ basis set uses d-aug-cc-pVQZ for the N and O atoms and aug-cc-pVQZ for the C and H atoms.

Figure 3 shows how the energies of the other electronic states vary with the intermolecular distance, *R*_NC_, for the ON–H_3_CH complex. The energy gap between the D_2_ and D_1_ states remain above 3.94 eV across all intermolecular distances despite the D_2_ potential energy curve ranging from weakly attractive to strongly repulsive. As such, there is no pathway for electronic quenching at low-collision energies for NO(*A*^2^Σ^+^) + CH_4_ in the ON–H_3_CH conformation. Figure S4 in the Supporting Information shows that the same behavior is observed with the NO–H_3_CH conformation. This is consistent with the experimental observation that the electronic quenching cross section of NO(*A*^2^Σ^+^) + CH_4_ at *T* = 296 K is less than 0.001 Å^2^.^12^

### NO + CH_3_OH

**Figure 4:**
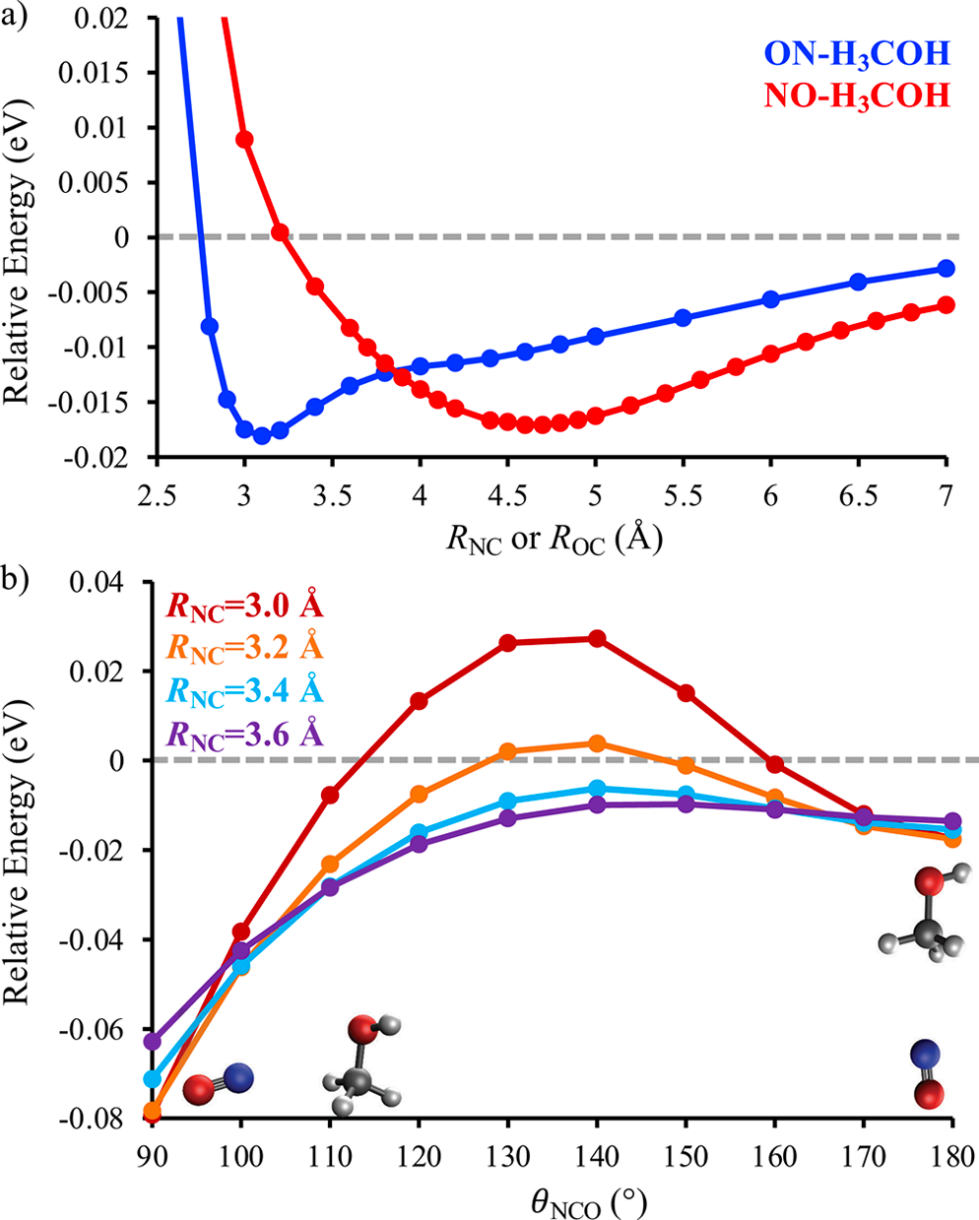
Panel (a) shows the energy of the D_2_ state as a function of the intermolecular distance when the NO is interacting with the CH_3_ group of CH_3_OH. The blue data are for ON–H_3_COH geometries, while the red data are for NO–H_3_COH geometries. Panel (b) shows the energy of the D_2_ state as a function of the intermolecular N–C–O angle (θ_NCO_) at fixed intermolecular distances *R*_NC_ = 3.0 Å (red data), *R*_NC_ = 3.2 Å (orange data), *R*_NC_ = 3.4 Å (blue data), and *R*_NC_ = 3.6 Å (purple data).The geometry optimizations were performed using EOM-EA-CCSD with a d-aug-cc-pVTZ basis set for the C, N, and O atoms and an aug-cc-pVDZ basis set for the H atoms. The single-point energies were evaluated at the EOM-EA-CCSD/d-aug-cc-pVTZ level of theory and all energies are reported relative to a D_2_-optimized geometry with an intermolecular distance of 20 Å.

We begin our analysis of this system by considering intermolecular orientations in which the NO(*A*^2^Σ^+^) is interacting with the CH_3_ face of CH_3_OH. Figure 4a demonstrates that the D_2_ PES supports weakly bound ON–H_3_COH and NO–H_3_COH complexes. For the ON–H_3_COH orientation, the strongest intermolecular attractions on D_2_ occur at an intermolecular distance of 3.1 Å, where the energy is −0.018 eV relative to an optimized geometry with an intermolecular distance of 20 Å. The most stable NO–H_3_COH geometry occurs at a significantly larger intermolecular distance, 4.6 Å, but has a comparable energy, −0.017 eV. Both minimum-energy geometries exhibit nearly C_3v_ symmetry and have large D_2_–D_1_ energy gaps of over 4.8 eV, suggesting that these pathways do not facilitate electronic quenching. Finally, as discussed in more detail in Figure S6 in the Supporting Information, the weak nature of these intermolecular attractions necessitated using a larger basis set for the geometry optimizations consisting of d-aug-cc-pVTZ for the C, N, and O atoms and aug-cc-pVDZ for the H atoms.

In order to ascertain the overall importance of the ON-H_3_COH complexes on the NO(*A*^2^Σ^+^) + CH_3_OH photochemistry, we show in Figure 4b how the D_2_ energy depends on the intermolecular N–C–O bond angle (θ_NCO_) at fixed intermolecular distances, *R*_NC_, between the nitrogen atom of NO and the carbon atom of CH_3_OH. This varies the relative intermolecular orientation from the NO being head-on with the CH_3_ face (θ_NCO_ = 180°) to the NO approaching the methyl group from the side (θ_NCO_ = 90°). At all four intermolecular distances considered in Figure 4b, the energy of the D_2_ state is lower when the NO interacts with the methyl group from the side rather than head-on. Figure 4b shows that when *R*_NC_ ≥ 3.6 Å, the D_2_ potential supports a nearly barrierless transition from the local minimum at θ_NCO_ = 180° to the lower-energy conformations with θ_NCO_ ≤ 90°. As *R*_NC_ decreases, a barrier grows between the local minimum at θ_NCO_ = 180° and the conformations with θ_NCO_ ≤ 90°. Overall, the flatness of the D_2_ potential at *R*_NC_ ≥ 3.6 Å and θ_NCO_ > 130° and the development of a barrier at smaller *R*_NC_ suggest that collisions that begin with the NO approaching the methyl group can become trapped in the local minimum at θ_NCO_ = 180°. For those collisions that reach conformations with θ_NCO_ ≤ 90°, unconstrained geometry optimizations on D_2_ show that the system will ultimately reach conformations in which the nitrogen of NO is interacting with the oxygen of CH_3_OH. Finally, Figure S7 in the Supporting Information shows that NO–H_3_COH conformations with θ_OCO_ = 180° are separated from lower-energy conformations with θ_OCO_ = 90° by a very small barrier of 0.002–0.003 eV. Unconstrained geometry optimizations from conformations with θ_OCO_ = 90° lead to geometries in which the oxygen of NO is interacting with the oxygen of CH_3_OH.

**Figure 5:**
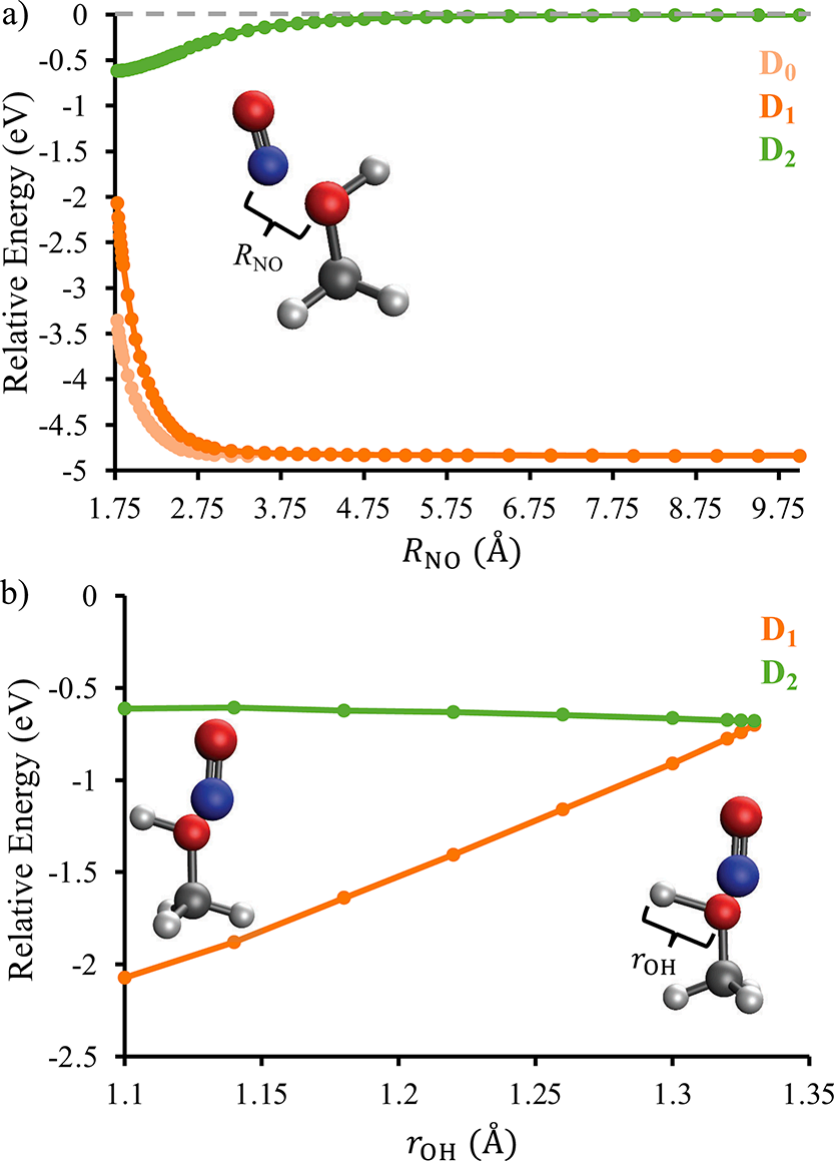
Panel (a) shows the energy of the D_0_ (light orange), D_1_ (orange), and D_2_ (green) states as a function of the intermolecular distance, *R*_NO_, when the N of NO is interacting with O of CH_3_OH. The inset shows the optimized geometry with *R*_NO_ = 1.78 Å. Panel (b) shows the energy of the D_1_ and D_2_ states as a function of the OH-bond length (*r*_OH_) at a fixed intermolecular distance of *R*_NO_ = 1.78 Å. The geometry optimizations were performed using EOM-EA-CCSD with a d-aug-cc-pVTZ basis set for the C, N, and O atoms and an aug-cc-pVDZ basis set for the H atoms. The single-point energies were evaluated at the EOM-EA-CCSD/d-aug-cc-pVTZ level of theory and all energies are reported relative to a D_2_-optimized geometry with an intermolecular distance of 20 Å.

Figure 5a shows how the electronic energies of the D_0_, D_1_, and D_2_ states varies with the intermolecular distance, *R*_NO_, for conformations in which the nitrogen of NO interacts with the oxygen of CH_3_OH. At large intermolecular distances, *R*_NO_ > 3.75 Å, the intermolecular interactions are very weakly attractive on D_2_. As the molecules move closer together, the strength of the intermolecular attractions increases significantly on D_2_, growing from  eV when *R*_NO_ = 3.95 Å to  eV when *R*_NO_ = 1.78 Å. In contrast, the D_0_ and D_1_ states become strongly repulsive as the intermolecular distance is reduced, with  increasing by over 2.75 eV when *R*_NO_ decreases from 3.95 to 1.78 Å. Figure S8 in the Supporting Information shows that the D_3_, D_4_, and D_5_ states remain well separated from the D_2_ throughout this range of intermolecular distances. Finally, note that the abrupt end to this figure reflects our difficulty obtaining converged optimized geometries at smaller intermolecular distances due to the presence of a D_2_–D_1_ conical intersection; we will return to this point below.

In order to understand the physical origin of the intermolecular attractions on D_2_, we analyze in Figure S9 in the Supporting Information the total Löwdin spin densities and partial charges on NO and CH_3_OH as a function of *R*_NO_. At large intermolecular distances, both molecules are neutral and the spin density is localized on the NO. As the molecules move closer together, the spin density begins to delocalize onto CH_3_OH, eventually becoming primarily localized onto CH_3_OH for *R*_NO_ ≤ 2.55 Å. As this occurs, NO develops a partial positive charge and CH_3_OH a partial negative charge, consistent with intermolecular electron transfer. Similar behavior was observed in our previous studies of the NO(*A*^2^Σ^+^) + CO and NO(*A*^2^Σ^+^) + H_2_O systems and is consistent with the harpoon mechanism, wherein the formation of a transient ion-pair increases the strength of the intermolecular attractions.^22,23,40−43^

In Figures S10–S12, we analyze the natural transition orbitals associated with the D_0_ → D_1_ and D_0_ → D_2_ transitions at representative intermolecular distances. Figure S10 shows that at large *R*_NO_, the D_1_ and D_2_ states exhibit clear 2*p*π* and 3*s*σ electronic character, respectively. As the molecules move closer together, the singly occupied molecular orbital (SOMO) of the D_2_ state becomes increasingly delocalized between the two molecules, with the region of space around the OH group of methanol especially gaining electron density. At *R*_NO_ = 1.78 Å, the SOMO for D_2_ has its greatest amplitude around the OH group of CH_3_OH; see Figure S12 in the Supporting Information. This further demonstrates that the D_0_ → D_2_ transition develops significant charge-transfer character as *R*_NO_ is reduced, consistent with the harpoon mechanism.

Returning to Figure 5, panel (b) shows clear evidence for a D_2_–D_1_ conical intersection at *R*_NO_ = 1.78 Å and an extended O–H bond length of *r*_OH_ ≈ 1.33 Å. On D_1_, stretching the O–H bond is associated with a significant increase in energy. In contrast, the D_2_ potential is nearly flat along this coordinate, with the energy decreasing from −0.61 to −0.68 eV along the path shown in Figure 5b. Figure S13 in the Supporting Information shows a similar D_2_–D_1_ conical intersection at an alternative conformation in which the NO lies above the methyl group of CH_3_OH and *R*_NO_ = 1.78 Å. Figure S14 in the Supporting Information shows that at larger intermolecular distances, increasing *r*_OH_ is not as energetically favorable on D_2_ and does not as readily decrease . For example, at *R*_NO_ = 1.90 Å,  increases from −0.56 eV at *r*_OH_ = 1.10 Å to −0.46 eV at *r*_OH_ = 1.33 Å, while  remains larger than 1.03 eV.

As with NO(*A*^2^Σ^+^) + H_2_O, we surmise that the pathways shown in Figure 5 support both nonreactive and reactive electronic quenching. Focusing first on nonreactive electronic quenching, Figure 5 shows an energetically downhill pathway toward a D_2_–D_1_ conical intersection that will facilitate rapid internal conversion. Because reaching the D_2_–D_1_ conical intersection requires the significant stretching of an O–H bond of CH_3_OH, we predict that nonreactive NO(*A*^2^Σ^+^) + CH_3_OH electronic quenching will produce CH_3_OH in a range of product states with vibrational excitation in the O–H stretch. The similarities between Figures 1 and 5b suggest a competing reactive electronic quenching pathway with products H + CH_3_ONO. Our calculations show that the reaction
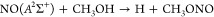
4is thermodynamically feasible. Specifically, the electronic Δ*E* = −52.8 kcal/mol when both products are generated in their ground electronic states and CH_3_ONO is in its trans-conformation.

We additionally considered potential electronic quenching pathways associated with stretching the O–C bond of methanol, *r*_OC_. From a thermodynamic perspective, the reaction
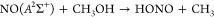
5is feasible, with an electronic Δ*E* = −77.8 kcal/mol when both products are generated in their ground electronic states and HONO is in its trans-conformation. In Figure S15 in the Supporting Information, we evaluate how the energies of the D_2_ and D_1_ states change as *r*_OC_ is increased at a fixed intermolecular distance of *R*_NO_ = 1.78 Å. These potential energy curves differ markedly from those shown in Figure 5b. The energy of the D_2_ state increases significantly as the O–C bond is stretched, ranging from −0.61 eV when *r*_OC_ = 1.44 Å to −0.30 eV when *r*_OC_ = 1.68 Å. At the same time, the D_2_ and D_1_ states remain energetically well separated, with  ranging from 1.53 to 1.36 eV. The absence of a D_2_–D_1_ conical intersection in Figure S15 along with the significant energetic cost of increasing *r*_OC_ suggest that this pathway will not play an important role in NO(*A*^2^Σ^+^) + CH_3_OH photochemistry.

### NO + CO_2_

We first considered the possibility that NO(*A*^2^Σ^+^) favorably interacts with the carbon atom of CO_2_. We analyzed both ON–CO_2_ and NO–CO_2_ complexes and varied the intermolecular distance and the angle NO makes with the CO_2_ (θ_ONC_ for ON–CO_2_ or θ_NOC_ for NO–CO_2_). Throughout these constrained geometry optimizations, the atom of NO interacting with CO_2_ was constrained to be at a 90° angle from one of the C=O bonds. As shown in Figure S16 in the Supporting Information, the intermolecular interactions in these conformations become increasingly repulsive as the intermolecular distance is reduced from 4.0 to 3.0 Å. This is true for all intermolecular orientations that we considered. As a result, we did not further consider conformations in which the NO(*A*^2^Σ^+^) directly interacts with the carbon atom of CO_2_.

**Figure 6:**
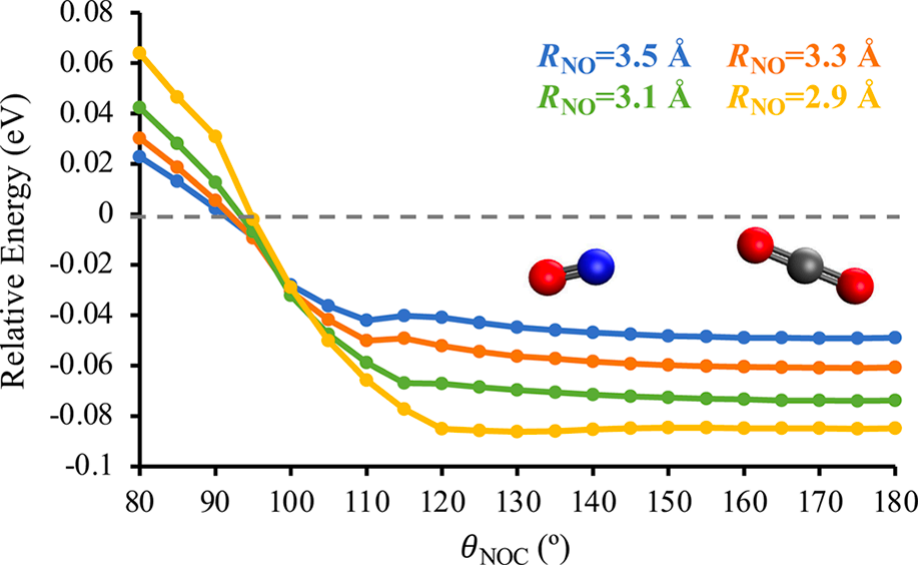
Energy of the D_2_ state of ON + OCO as a function of the intermolecular angle θ_NOC_ at the intermolecular distances *R*_NO_ = 3.5 Å (blue), *R*_NO_ = 3.3 Å (orange), *R*_NO_ = 3.1 Å (green), and *R*_NO_ = 2.9 Å (yellow). These data were calculated at the EOM-EA-CCSD/AVQZ//EOM-EA-CCSD/aug-cc-pVDZ level of theory. All energies are reported relative to a D_2_-optimized geometry with an intermolecular distance of 20 Å.

We next considered pathways in which the nitrogen atom of NO interacts with one of the oxygen atoms of CO_2_. Figure 6 shows how the energy of the D_2_ state varies with the intermolecular bond angle θ_NOC_ for the larger intermolecular distances, *R*_NO_, of 3.5, 3.3, 3.1, and 2.9 Å. These data were obtained by performing constrained geometry optimizations on D_2_ at fixed values of θ_NOC_ and *R*_NO_ using the EOM-EA-CCSD/AVQZ//EOM-EA-CCSD/aug-cc-pVDZ level of theory; the AVQZ basis consists of d-aug-cc-pVQZ for the N and O atoms of NO and aug-cc-pVQZ for the C and O atoms of CO_2_. The conformations analyzed in Figure 6 are all nearly planar; non-planar geometries were consistently found to be higher in energy. For each of the intermolecular distances, the energy increases as θ_NOC_ decreases, suggesting an energetic preference for the nitrogen atom of NO and an oxygen atom of CO_2_ to approach each other nearly head-on. Additionally, the intermolecular attractions between the two molecules increase as *R*_NO_ decreases, causing the two molecules to move closer together.

In Figures S17–S19 in the Supporting Information, we compare the data summarized in Figure 6 to conformations in which the oxygen atom of NO is interacting with one of the oxygen atoms of CO_2_. The qualitative picture for these NO + OCO conformations is the same as that shown in Figure 6. However, the energies of the NO + OCO conformations are consistently less attractive than the corresponding ON + OCO conformations by as much as 0.023 eV. This indicates that while there are attractive intermolecular interactions for NO + OCO conformations, they are not as favorable as the orientations shown in Figure 6.

**Figure 7:**
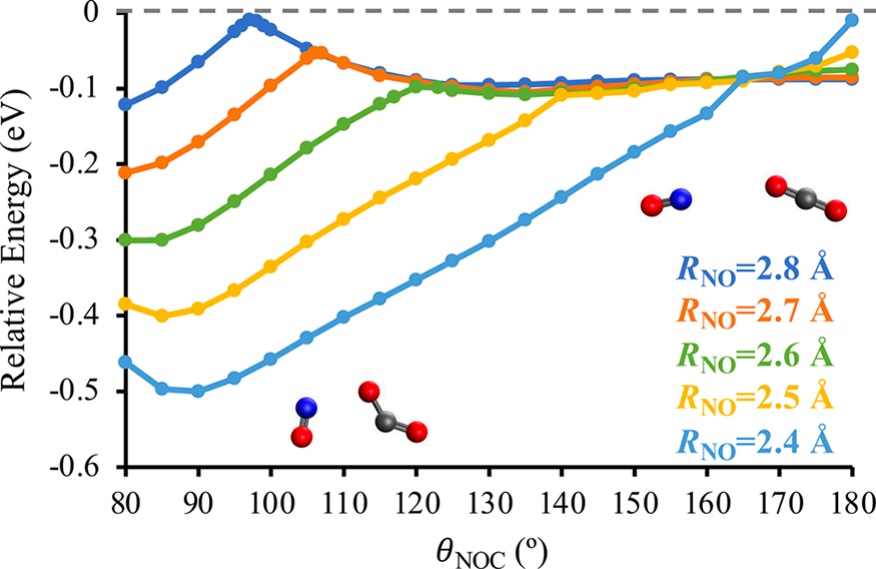
Energy of the D_2_ state of ON + OCO as a function of the intermolecular angle θ_NOC_ at the intermolecular distances *R*_NO_ = 2.8 Å (blue), *R*_NO_ = 2.7 Å (orange), *R*_NO_ = 2.6 Å (green), *R*_NO_ = 2.5 Å (yellow), and *R*_NO_ = 2.4 Å (light blue). These data were calculated at the EOM-EA-CCSD/AVQZ//EOM-EA-CCSD/aug-cc-pVDZ level of theory. All energies are reported relative to a D_2_-optimized geometry with an intermolecular distance of 20 Å. As indicated by the insets, the low-energy conformations at smaller θ_NOC_ are associated with the linear-to-bent isomerization of CO_2_.

As shown in Figure 7, the cuts of the D_2_ PES along θ_NOC_ are dramatically different for intermolecular distances *R*_ON_ ≤ 2.8 Å then what is seen in Figure 6. For *R*_NO_ = 2.8 Å and *R*_NO_ = 2.7 Å, there is a general increase in energy as θ_NOC_ decreases until a barrier is reached around θ_NOC_ = 97° and  eV for *R*_NO_ = 2.8 Å and θ_NOC_ = 107° and  eV for *R*_NO_ = 2.7 Å. At smaller θ_NOC_ after the barrier, the energy sharply decreases as CO_2_ adopts a bent conformation with a O–C–O bond angle of around 140°. In addition, the linear to bent isomerization of CO_2_ is accompanied by an elongation of both C–O bond lengths, *r*_OC_, with the largest elongation occurring at the C–O bond that is interacting with the NO. For example, at *R*_ON_ = 2.7 Å, the *r*_OC_ are 1.167 and 1.172 Å when θ_NOC_ = 180° and 1.218 and 1.258 Å when θ_NOC_ = 80°. The barrier preceding the linear to bent isomerization of CO_2_ further decreases at *R*_NO_ = 2.6 Å and disappears for *R*_NO_ ≤ 2.5 Å. As such, the D_2_ PES drives ON–OCO into conformations in which the CO_2_ is significantly bent. Finally, Figure 7 shows that the strength of the attractive intermolecular interactions between the two molecules increases as the molecules move closer together. For example, at *R*_NO_ = 2.8 Å, the minimum energy is −0.12 eV, while at *R*_NO_ = 2.4 Å, the minimum energy is −0.50 eV. At the minimum energy geometry with *R*_NO_ = 2.4 Å, the O–C–O angle is 138.4° and the *r*_OC_ are 1.210 and 1.268 Å.

Figure S20 shows the energies of the D_1_ and D_2_ states as a function of θ_NOC_ for the representative intermolecular distances *R*_NO_ = 2.8 Å, *R*_NO_ = 2.6 Å, and *R*_NO_ = 2.4 Å. The transition from linear to bent CO_2_, which causes a drop in the energy of the D_2_ state, results in a significant increase in the energy of the D_1_ state. As a result, this isomerization causes the two states to become significantly closer together in energy. For example, at *R*_NO_ = 2.4 Å, the D_2_–D_1_ energy gap, , is 4.80 eV when θ_NOC_ = 180° and 2.68 eV when θ_NOC_ = 160°, where CO_2_ first isomerizes into a bent geometry. Collectively, our analysis suggests that decreasing *R*_NO_ and the associated linear-to-bent isomerization of CO_2_ play a significant role in pushing ON + CO_2_ toward a D_2_–D_1_ conical intersection that can facilitate electronic quenching.

Figure S21 in the Supporting Information extends the analysis shown in Figure 7 to NO + OCO conformations. Similar to the *R*_NO_ = 2.8 Å and *R*_NO_ = 2.7 Å data in Figure 7, the energy of D_2_ initially increases as *θ*_OOC_ decreases, reaching a barrier that precedes a drop in energy associated with CO_2_ adopting a bent geometry. The energies in Figure S21 are significantly less negative than those shown in Figure 7, indicative of weaker intermolecular attractions in NO + OCO conformations than ON + OCO conformations. The intermolecular attractions in NO + OCO conformations become even weaker as the molecules move closer together. Moreover, the barrier preceding the linear-to-bent isomerization of CO_2_ in the NO + OCO conformations fall above the asymptotic limit. This is quite different from Figure 7, where the barrier to bent CO_2_ for ON + OCO conformations consistently lies below the asymptotic limit and eventually disappears at closer intermolecular distances. Figure S21 therefore provides additional support for NO + OCO conformations being significantly less important for electronic quenching than ON–OCO conformations.

**Figure 8:**
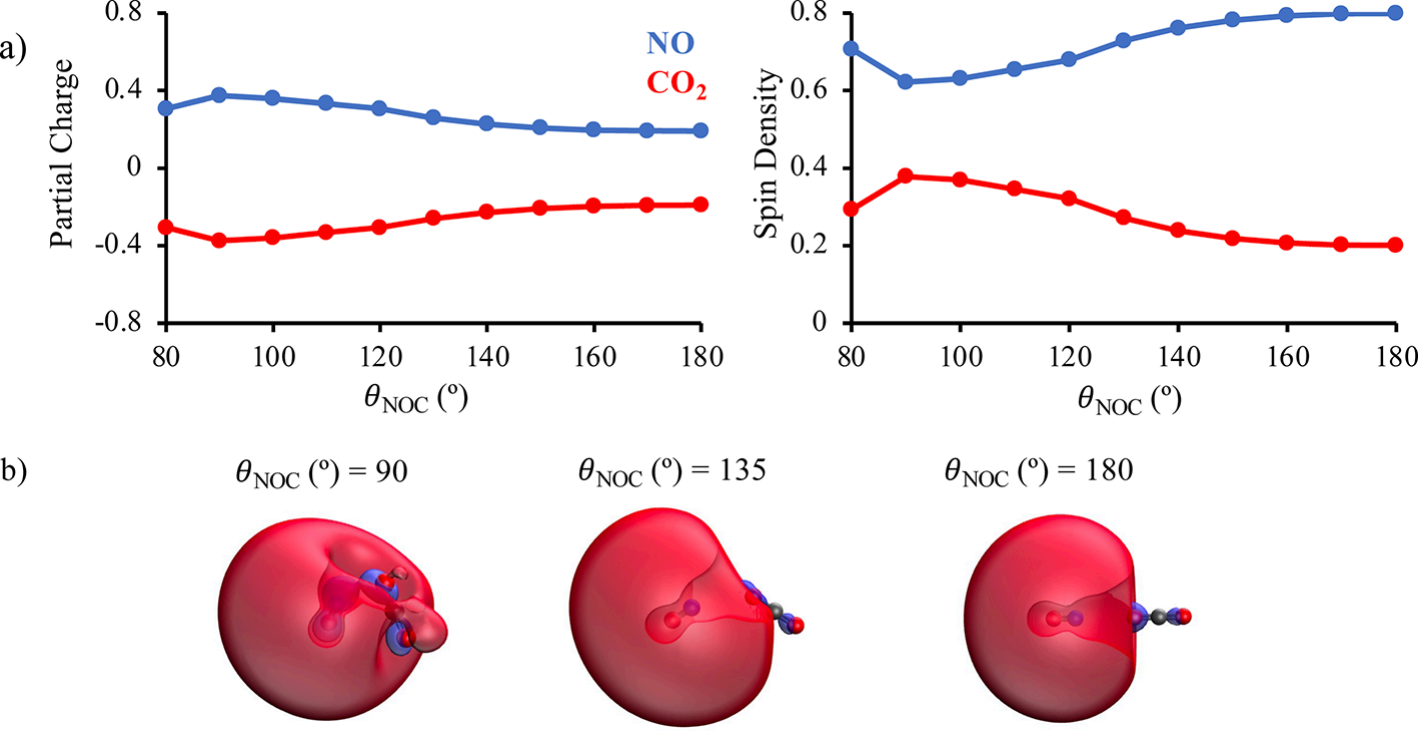
Panel (a) shows the Löwdin population analysis of the total spin densities and partial charges of NO and CO_2_ as a function of θ_NOC_ at *R*_NO_ = 2.9 Å. The total spin density of a molecule is obtained by summing together the spin densities of all atoms belonging to that molecule. The total partial charges are obtained analogously. Panel (b) shows the SOMOs for the D_2_ state of ON + OCO at representative θ_NOC_ and *R*_NO_ = 2.9 Å.

**Figure 9:**
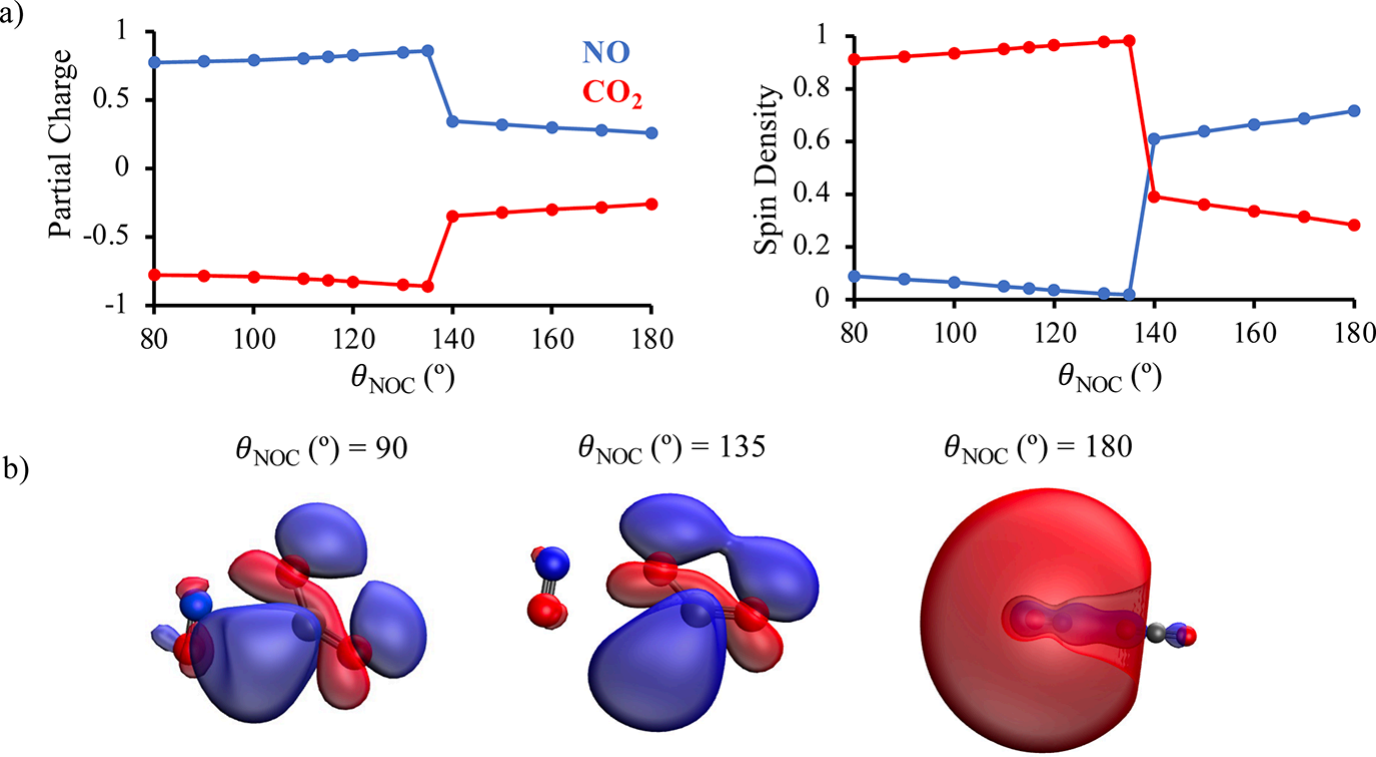
Panel (a) shows the Löwdin population analysis of the total spin densities and partial charges of NO and CO_2_ as a function of θ_NOC_ at *R*_NO_ = 2.5 Å. The total spin density of a molecule is obtained by summing together the spin densities of all atoms belonging to that molecule. The total partial charges are obtained analogously. Panel (b) shows the SOMOs for the D_2_ state of ON + OCO at representative θ_NOC_ and *R*_NO_ = 2.5 Å. Note that the electronic character of the SOMO changes from 3*s*σ on NO at θ_NOC_ = 180° to 2*p*π* on CO_2_ for θ_NOC_ < 140°. This, along with the increased charge and spin density on CO_2_ for θ_NOC_ < 140°, is consistent with electron transfer occurring between the two molecules, i.e., the harpoon mechanism.

In order to better understand the origin of the attractive intermolecular interactions between NO(*A*^2^Σ^+^) and CO_2_ shown in Figures 6 and 7, we analyze in Figures 8 and 9 the Löwdin partial charges and spin densities for ON + OCO conformations at *R*_ON_*=* 2.9 and *R*_ON_*=* 2.5 Å. These figures additionally show the singly occupied molecule orbital (SOMO) of the D_2_ state at representative θ_NOC_. The SOMOs shown in Figure 8 demonstrate that at *R*_ON_*=* 2.9 Å, an intermolecular distance where the linear-to-bent isomerization of CO_2_ does not occur, the electron in the 3*s*σ Rydberg orbital on NO becomes somewhat delocalized onto CO_2_ as θ_NOC_ is reduced. As a result, the Löwdin analysis reveals evidence of charge-transfer, with CO_2_ gaining a partial charge ranging from −0.19 to −0.37 and a spin density ranging from 0.20 to 0.38. Figure 9 shows that at *R*_ON_*=* 2.5, a distance where CO_2_ does become bent for θ_NOC_ < 140°, there is a dramatic change in the distribution of electron density in the complex. Specifically, CO_2_ develops a partial charge of approximately −0.77 and nearly all of the spin density becomes localized on CO_2_, both of which clearly indicate electron transfer. At the same time, the SOMO changes from having 3*s*σ character on NO to 2*p*π* character on the CO_2_. As such, the un-paired electron has transferred from a diffuse Rydberg orbital centered on NO to the LUMO of CO_2_.

Collectively, the analysis presented in Figure 9 allows us to rationalize several aspects of the D_2_ PES shown in Figure 7. Specifically, the strong intermolecular attractions on the D_2_ state of ON + OCO originate from the harpoon mechanism in which electron transfer from NO to CO_2_ creates a transient ion-pair which coulombically attract one another. Moreover, the linear-to-bent isomerization of CO_2_ reflects electron transfer from NO(*A*^2^Σ^+^) to CO_2_ as the CO_2_ radical anion is experimentally known to exist in a significantly bent geometry with an O–C–O angle of approximately 127° ± 8°.^44^

**Figure 10:**
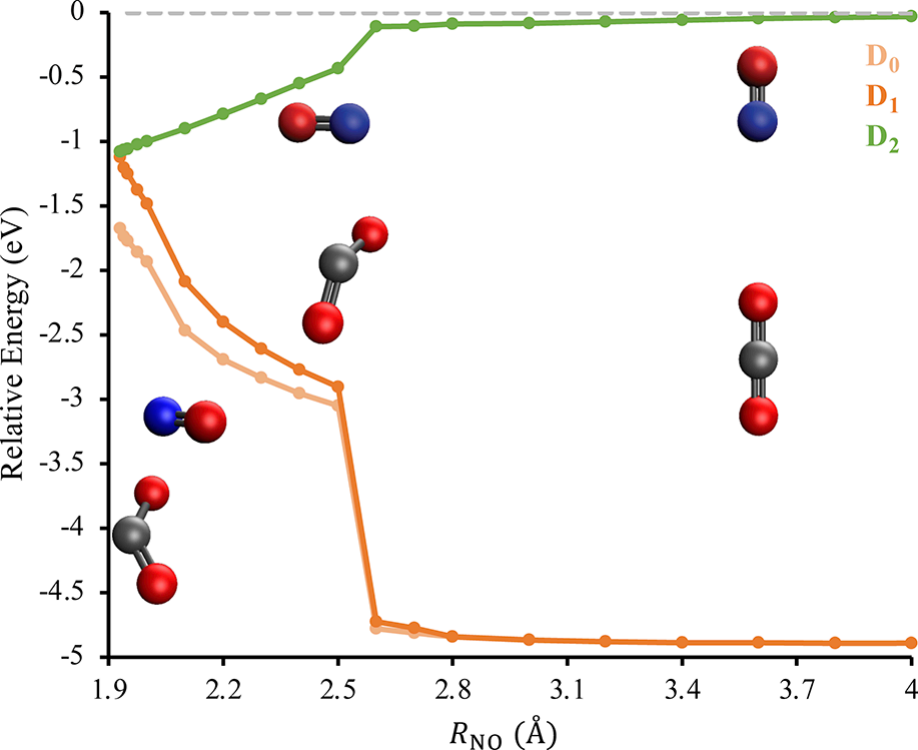
Energy of the D_0_ (light orange), D_1_ (orange), and D_2_ (green) states as a function of the intermolecular distance, *R*_NO_, when the N of NO is interacting with an O of CO_2_. The insets show representative molecular geometries along this pathway. These calculations were performed at the EOM-EA-CCSD/AVQZ//EOM-EA-CCSD/aug-cc-pVTZ level of theory and all energies are reported relative to a D_2_-optimized geometry with an intermolecular distance of 20 Å.

An overview of the photochemical pathway responsible for NO(*A*^2^Σ^+^) + CO_2_ electronic quenching is provided in Figure 10. Note that we employed the larger aug-cc-pVTZ basis set for these geometry optimizations to obtain an improved description of the region in the vicinity of the D_2_–D_1_ conical intersection. Consistent with Figure 6, the two molecules initially approach each other head-on in a linear arrangement and experience relatively weak intermolecular attractions ranging from −0.03 to −0.11 eV as *R*_NO_ is reduced. As in Figure 7, the sudden drop in  at *R*_NO_ = 2.5 Å is associated with CO_2_ adopting a bent conformation. As shown by the inset in Figure 10, the lowest energy complex at this intermolecular distance is non-planar. The D_2_ potential becomes significantly more attractive after the linear-to-bent isomerization of the CO_2_. Figure 10 further suggests that the D_2_ PES is effective at funneling population to a D_2_–D_1_ conical intersection which occurs at approximately *R*_NO_ = 1.93 Å and  eV. At the approximate D_2_–D_1_ conical intersection, the CO_2_ molecule has an O–C–O bond angle of 133.1° and O–C bond lengths of 1.30 and 1.19 Å, while the N–O bond length of NO is 1.09 Å. Comparing this to the geometry at *R*_NO_ = 20 Å, where the O–C–O bond angle is 180.0°, the O–C bond lengths are 1.16 Å, and the N–O bond length is 1.06 Å, we see that the pathway shown in Figure 10 causes a significant distortion to the geometry of CO_2_ and a smaller change to the bond length NO.

Figure S23 in the Supporting Information shows an alternative pathway to a D_2_–D_1_ conical intersection for ON + OCO in which the O atom of NO remains oriented away from the C atom of CO_2_ throughout. Beginning at *R*_NO_ = 2.6 Å, the system adopts a planar conformation with a bent CO_2_. As in Figure 10, the linear-to-bent isomerization of CO_2_ is associated with a significant increase in the attractive intermolecular interactions on D_2_. The D_2_ potential funnels the two molecules closer together until a D_2_–D_1_ conical intersection is reached. This D_2_–D_1_ conical intersection occurs at a nonplanar geometry at approximately *R*_NO_ = 1.925 Å and  eV. At this approximate D_2_–D_1_ conical intersection, the O–C–O bond angle is 134.3°, the O–C bond lengths are 1.30 and 1.18 Å, and the N–O bond length is 1.09 Å.

We now turn to the connection between our computational analysis of NO(*A*^2^Σ^+^) + CO_2_ photochemistry and existing experimental data. The large room temperature electronic quenching cross section of NO(*A*^2^Σ^+^) + CO_2_ reported in Table 1 is consistent with the presence of multiple pathways to D_2_–D_1_ conical intersections that are downhill in energy from the asymptotic limit. Moreover, our analysis suggests that the D_2_ PES can effectively funnel a wide range of initial intermolecular orientations to D_2_–D_1_ conical intersections, particularly those in which the N atom of NO is oriented toward an O atom of CO_2_. The significant distortions to the geometry of CO_2_ observed at our approximate D_2_–D_1_ conical intersections are consistent with the experimental observation that NO(*A*^2^Σ^+^) + CO_2_ electronic quenching releases a large fraction of the available energy, approximately 62%, into the vibrational degrees of freedom of CO_2_.^8^ In particular, the pathways shown in Figures 10 and S23 are consistent with the formation of CO_2_ with significant vibrational excitation in its bending and asymmetric stretching modes. Additionally, experiments show a clear preference for NO(*X*^2^Π) being produced with ν_NO_^′^ = 0 or ν_NO_^′^ = 1.^7^ This is consistent with the fact that the NO geometry is not nearly as distorted at the D_2_–D_1_ conical intersection as CO_2_; the N–O bond length is 1.09 Å at the D_2_–D_1_ conical intersection and 1.16 Å at the equilibrium geometry of NO(*X*^2^Π).

Turning to reactive quenching, we do not believe that the pathways shown in Figures 10 and S23 support the direct production of CO + O(^3^P) atoms via eq 2a for several reasons. First, these pathways only involve a modest increase in the O–C bond length of around 0.14 Å. In contrast, the pathways to a D_2_–D_1_ conical intersection for NO + H_2_O and NO + CH_3_OH shown in Figures 1 and 5 require an O–H bond to stretch by a significantly greater amount, 0.36–0.41 Å. Second, because the most elongated O–C bond in Figures 10 and S23 contains the O atom that is interacting with the NO, these molecular geometries appear more consistent with the formation of NO_2_ + CO via eq 2b than NO(*X*^2^Π) + CO + O(^3^P). This contrasts with the pathways shown in Figures 1 and 5 where the H atom of the stretched O–H bond is not directly interacting with the NO and hence can freely dissociate from the complex. Finally, the geometries of the D_2_–D_1_ conical intersections shown in Figures 10 and S23 more closely resemble NO + CO_2_ than the products of either eq 2a or 2b, consistent with nonreactive electronic quenching. Moreover, upon internal conversion from D_2_ to D_1_, the D_1_ PES will rapidly drive the NO(*X*^2^Π) and CO_2_ molecules apart.

Based on our computational analysis and the existing experimental data, we hypothesize that the experimentally observed production of CO originates from the reaction between highly vibrationally excited CO_2_ produced through eq 1 and NO(*X*^2^Π)
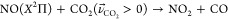
6Using standard thermodynamic values, eq 6 becomes exothermic if greater than 42.96% of the available energy from the NO(*A*^2^Σ^+^) + CO_2_ electronic quenching is partitioned into the vibrational degrees of freedom of the CO_2_. Hancock and co-workers experimentally demonstrated that NO(*A*^2^Σ^+^) + CO_2_ electronic quenching produces highly vibrationally excited CO_2_, with approximately 62% of the available energy partitioned into the vibrational degrees of freedom of CO_2_. As such, NO(*A*^2^Σ^+^) + CO_2_ electronic quenching readily produces CO_2_ with enough internal energy to make eq 6 thermodynamically favorable. Note that eq 6 also accounts for the formation of NO_2_ which has also been observed experimentally.

## Conclusions

Our exploration of the electronic quenching of NO(*A*^2^Σ^+^) with molecular partners provides new insights into the photochemistry of open-shell molecular systems. In the case of NO(*A*^2^Σ^+^) + CH_4_, the only attractive complexes are of C_3v_ symmetry, which quickly become repulsive at smaller intermolecular distances. The absence of a low-energy D_2_–D_1_ conical intersection is consistent with the near-zero electronic quenching cross sections of NO(*A*^2^Σ^+^) + CH_4_ observed experimentally. Our work supports the experimental observation that the photodissociation of NO(*A*^2^Σ^+^) + CH_4_ produces NO(*A*^2^Σ^+^) in a broad range of final rotational states, while imparting little rotational energy to CH_4_. Specifically, upon NO(*X*^2^Π) + CH_4_→NO(*A*^2^Σ^+^) + CH_4_ electronic excitation, the NO re-orients itself from being perpendicular with the intermolecular bond (C_s_ symmetry) to being oriented head-on with the CH_3_ face (C_3v_ symmetry). As a result, vibrational relaxation on the D_2_ PES away from the Franck–Condon region imposes a torque on the NO, consistent with NO(*A*^2^Σ^+^) receiving much more rotational energy than CH_4_ when the complex dissociates.

NO(*A*^2^Σ^+^) + CH_3_OH presents a very different story. Although there are weakly bound complexes where NO interacts with the CH_3_ face, the strongest attractive intermolecular interactions occur in conformations where the N atom of NO interacts with the O atom of CH_3_OH. We attribute the strong attractive intermolecular interactions on D_2_ to the harpoon mechanism, with transient electron transfer occurring from NO(*A*^2^Σ^+^) to CH_3_OH. In addition, similar to NO(*A*^2^Σ^+^) + H_2_O, our results suggest that NO(*A*^2^Σ^+^) + CH_3_OH has the potential to undergo both reactive and nonreactive electronic quenching. This is because the downhill pathway to a D_2_–D_1_ conical intersection involves both decreasing the intermolecular distance and significantly stretching the O–H bond of CH_3_OH. The reactive pathway will produce H and CH_3_ONO, while the nonreactive pathway will result in CH_3_OH being formed in a range of product states with a vibrational progression in the O–H stretch. Future experimental and ab initio dynamics studies on this system are warranted to test the mechanistic predictions made in this study as well as determine the relative branching between reactive and non-reactive electronic quenching.

Finally, we discuss the conclusions of our mechanistic study of the NO(*A*^2^Σ^+^) + CO_2_ system. We showed that the most energetically favorable conformations on the D_2_ PES have the N atom of NO interacting with an O atom of CO_2_. The attractive intermolecular interactions increase as the molecules grow closer together and eventually drive the isomerization of CO_2_ into a bent conformation. The linear-to-bent isomerization of CO_2_ results in significantly increased intermolecular attractions and reflects the formation of a transient NO^+^ + CO_2_^–^ ion pair through the harpoon mechanism. We further showed that the D_2_ PES provides multiple, energetically downhill pathways to D_2_–D_1_ conical intersections which facilitate electronic quenching. These pathways induce significant geometric distortions to CO_2_, consistent with the experimental observation that NO(*A*^2^Σ^+^) + CO_2_ electronic quenching releases a large fraction of the available energy into the vibrational degrees of freedom of CO_2_. Finally, we hypothesize that the highly vibrationally excited CO_2_ produced through NO(*A*^2^Σ^+^) + CO_2_ electronic quenching will subsequently react with ground-state NO to produce the experimentally observed CO and NO_2_. Future velocity map imaging experiments, in conjunction with ab initio dynamics simulations, on this system are needed to shed light on the electronic quenching pathways responsible for producing NO(*X*^2^Π, ν_NO_^′^ ≤ 1) as well as test whether the CO and NO_2_ products are produced directly (eqs 2a–2c) or through the subsequent reaction of vibrationally hot CO_2_ (eq 6).

## Supporting Information Available

PESs of the NO + CH_4_ system along with tabulated data to construct all PES plots; PESs of the NO + CH_3_OH system and tabulated data to construct all PES plots: Löwdin spin and partial charge plots for NO + CH_3_OH at various *R*_NO_; SOMOs for NO + CH_3_OH over various intermolecular distances; comparison of O–H and O–C stretching pathways in the NO + CH_3_OH system with tabulated values; comparison of pathways for the NO + CO_2_ system in which the C of CO_2_ is interacting with the NO; comparison between the D_2_ states of the NO + CO_2_ system with either the O or N of NO approaching the O of CO_2_ at various intermolecular distances; PESs of both long and short-range intermolecular distances based on the θ_NOC_ or θ_OOC_ of both NO + OCO and ON + OCO complexes along with tabulated data; PESs of conical intersection pathways for the NO + CO_2_ system with the tabulated data; and results of assessing the basis set dependence for NO + CH_4_ and NO + CO_2_ (PDF)
