# Supplementary material for: Theoretical Study of the Photochemical Mechanisms of the Electronic Quenching of NO(A2Σ+) with CH4, CH3OH, and CO2
Source: J Phys Chem A. 2023 Aug 8;127(34):7228–40. doi: 10.1021/acs.jpca.3c03981 (PMC10476188; doi:10.1021/acs.jpca.3c03981)
Supplement: Supplementary file 1 — jp3c03981_si_001.pdf [file jp3c03981_si_001.pdf]

Supporting Information for "Theoretical Study of the Photochemical Mechanisms of the Electronic Quenching of NO ($A^2\Sigma^+$) with CH_4, CH_3OH, and CO_2"

Aerial N. Bridgers,[1] Justin A. Urquilla,[1] Julia Im,[1] Andrew S. Petit*[1]

[1] *Department of Chemistry and Biochemistry, California State University – Fullerton, Fullerton, CA 92834-6866, USA*

* Author to whom correspondence should be addressed. E-mail: apetit@fullerton.edu

Table of Contents

	Page(s)
**Figure S1**: Example of the three-point extrapolation to the complete basis set limit used for NO+CH_4.	S5
**Figures S2-S3**: D_2 energy comparison at different angles for NO+H_3CH and ON+H_3CH	S6-S7
**Figure S4**: Energies of the D_0 to D_5 states of the NO-H_3CH complex	S8
**Figure S5**: Basis set dependence of the D_2 energy versus R_{NC} for ON-H_3CH.	S9
**Figure S6**: Dependence of the D_2 PES along R_{NC} for ON-H_3COH on the basis set used for the geometry optimization.	S10
**Figures S7**: Energy of the D_2 state of NO-CH_3OH as a function of θ_{OCO} at R_{OC}=5.0 Å and R_{OC}=4.6 Å.	S11
**Figure S8**: Energies of the D_0 to D_5 states of ON+CH_3OH as a function of R_{NO} when the N of NO interacts with the O of CH_3OH.	S12
**Figure S9**: Löwdin spin density and partial charge analysis of NO+CH_3OH at various R_{NO}	S13
**Figures S10-S12**: Molecular orbital analysis of the ON+CH_3OH system	S14-S16
**Figure S13-S14**: Energies of the D_1 and D_2 states as a function of stretching the O-H bond of CH_3OH at various orientations of the complex.	S17-S18
**Figure S15**: Energies of the D_1 and D_2 states as a function of stretching the O-C bond of CH_3OH.	S19
**Figure S16**: Energies of the NO+CO_2 system with either the N or O approaching the carbon center	S20
**Figures S17-S19**: Comparison of the D_2 energy state of the NO+CO_2 system as a function of θ_{NOC} or θ_{OOC} at various intermolecular distances	S21-S23
**Figure S20**: The energy of the D_1 and D_2 states of ON+OCO as a function of the intermolecular angle θ_{NOC} at R_{NO}=2.8 Å, 2.6 Å, and 2.4 Å.	S24
**Figure S21**: The energy of the D_2 state of NO+OCO as a function of the intermolecular angle θ_{OOC} at R_{OO}=2.9 Å, 2.7 Å, 2.5 Å, and 2.4 Å.	S25
**Figure S22:** Energies of the D_0 to D_5 states shown for the pathway to a D_2-D_1 conical intersection shown in Figure 10.	S26
**Figure S23**: An alternative pathway to a D_2-D_1 conical intersection for NO+CO_2	S27

**Figures S24-S33**: Comparison between single-point energies evaluated using EOM-EA-CCSD/AVQZ and EOM-EA-CCSD/d-aug-ccpVTZ for the NO+CO_2 system.	S28-S32
**Figure S34**: Energies of the D_0, D_1, and D_2 states of ON-OCO as a function of R_{NO} evaluated using EOM-EA-CCSD/d-aug-ccpVTZ for the single-point energies.	S33
**Table S1:** Numerical values for the D_2 energy at different intermolecular angles for NO+H_3CH and ON+H_3CH; this data is plotted in Figures S2-S3.	S34
**Table S2**: Numerical values for the D_2 energy at different intermolecular distances for NO+H_3CH and ON+H_3CH; this data is plotted in Figure 2.	S35
**Table S3-S4:** Numerical values of the energies of the D_0 to D_5 states of various NO+CH_4 orientations shown in Figures 3 and S4.	S36-S37
**Tables S5**: Numerical values for the energy of the D_2 state as a function of the intermolecular distance when the NO is interacting with the CH_3 group of CH_3OH; this data is plotted in Figure 4a.	S38
**Table S6**: Numerical values of the energy of the D_2 state in Figure 4 as a function of the N-C-O angle (θ_{NCO}) at fixed intermolecular distances; this data is plotted in Figure 4b.	S39
**Table S7:** Numerical values of the data plotted in Figure S6.	S40
**Table S8:** Numerical values of the energies of the D_2 state of NO+CH_3OH as a function of θ_{OCO} at different intermolecular distances; this data is plotted in Figure S7.	S41
**Table S9:** Numerical values of the energies of the D_0 to D_5 states of NO+CH_3OH at various R_{NO}; this data is plotted in Figures 5a and S8.	S42-S43
**Table S10**: Numerical values of the energy of the D_1 and D_2 states as a function of the OH-bond length (r_{OH}) at a fixed intermolecular distance of R_{NO}=1.78 Å; this data is plotted in Figure 5b.	S44
**Tables S11-13:** Numerical values of the various energy states of the stretching of the O-H bond or O-C bond in CH_3OH with various constraints and at various intermolecular distances; this data is plotted in Figures S13-S15.	S45-S47
**Table S14:** Numerical values for the energies for the D_2 states of two different molecular orientations, NO-CO_2 and ON-CO_2, as a function of the intermolecular bond angles θ_{ONC} or θ_{NOC} at representative intermolecular distances; this data is plotted in Figure S16.	S47
**Table S15:** Numerical values for the energies of the D_2 states of ON-OCO as a function of θ_{NOC} for intermolecular distances R_{NO}=3.5 Å, 3.3 Å, 3.1 Å, and 2.7 Å; this data is plotted in Figure 6.	S48
**Tables S16-S18:** Numerical values of the comparison of the D_2 energy state of the NO+CO_2 system as a function of θ_{NOC} or θ_{OOC} at various intermolecular distances; this data is plotted in Figures S17-S19.	S49-S51

**Tables S19-S20.** Numerical values of the energies of the D_2 states of ON-OCO as a function of θ_{NOC} for intermolecular distances R_{NO}=2.8-2.4 Å; this data is plotted in Figure 7.	S52-S53
**Tables S21-S22:** Numerical values of the energies of the D_1 and D_2 states of ON-OCO as a function of θ_{NOC} for the intermolecular distances R_{NO}=2.8 Å, 2.6 Å, and 2.4 Å; this data is plotted in Figure S20.	S54-S55
**Table S23:** Numerical values of the energies of the D_2 states of NO-OCO as a function of θ_{OOC} for R_{OO}=2.9-2.4 Å; this data is plotted in Figure S21.	S56
**Tables S24-S25:** Numerical values of the energies of the D_0, D_1, and D_2 states of ON-OCO as a function of R_{NO} for two different conformations; this data is plotted in Figures 8 and S22.	S57-S58

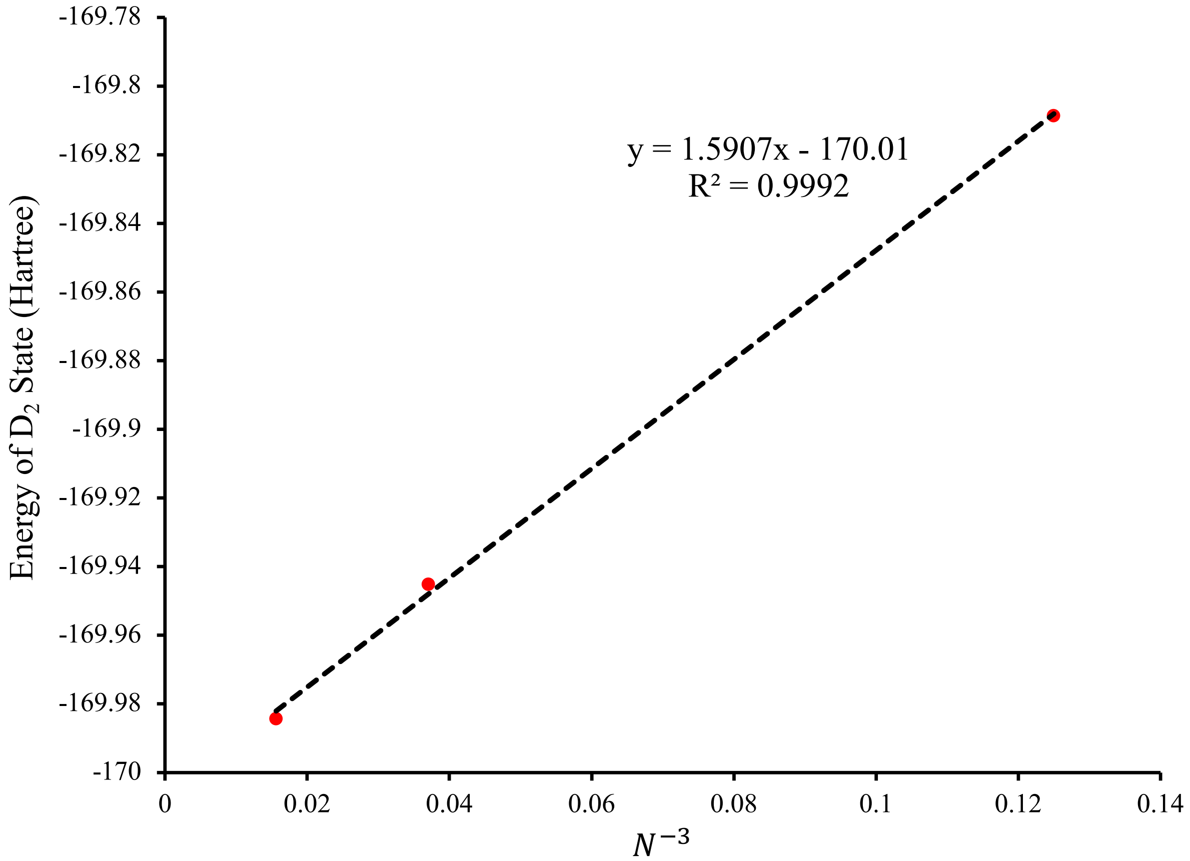

Figure S1. Example of the three-point extrapolation to the complete basis set limit performed for the D_2 state of NO+CH_4. Similar quality fits are obtained at all of the geometries analyzed in this study. As described in the text, this extrapolation assumes the following functional form:

$$E^{AVNZ}(N) = E^{CBS} + \alpha N^{-3}$$

where AVNZ is a basis set with d-aug-cc-pVNZ for the N and O atoms and aug-cc-pVNZ for the C and H atoms.

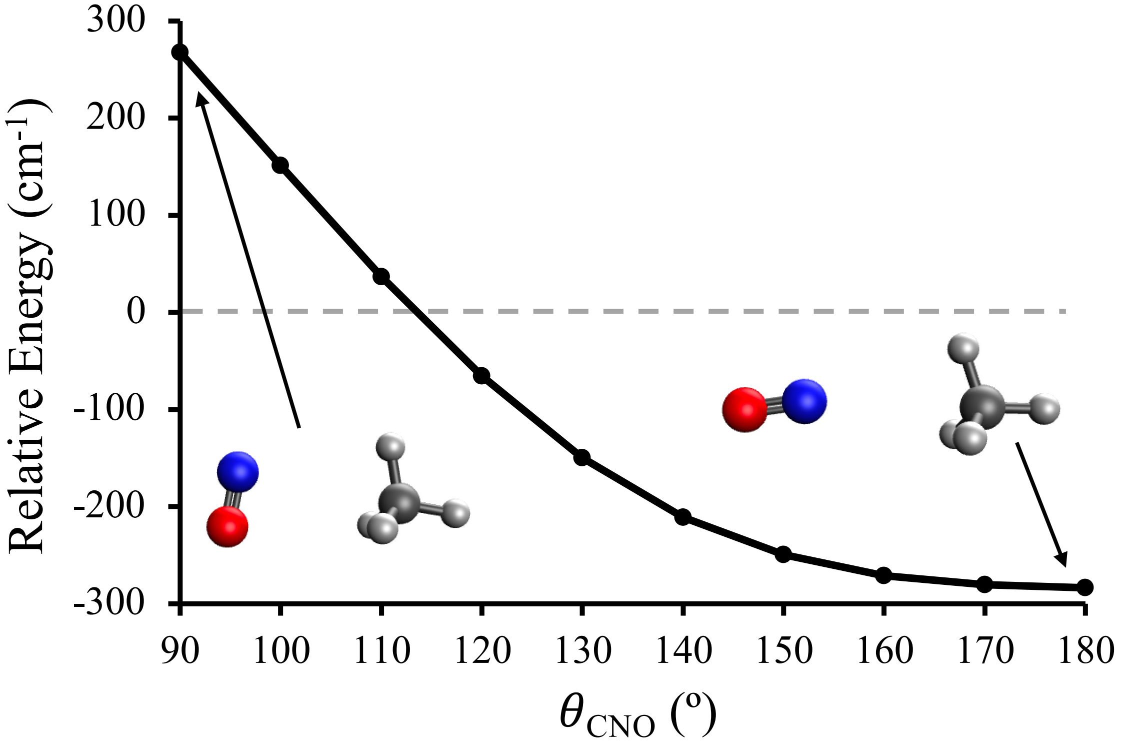

Figure S2. Dependence of the energy of the D_2 state of ON+H_3CH as a function of θ_{CNO}, the angle between the carbon atom of CH_4 and the NO, at a fixed intermolecular distance of R_{NC}=3.15 Å. The geometry optimizations were calculated using EOM-EA-CCSD/aug-cc-pVTZ and a three-point extrapolation to the CBS limit was performed for the electronic energies. All energies are reported relative to a D_2-optimized geometry with an intermolecular distance of 20 Å. The energies increase significantly as the θ_{CNO} decreases, displaying the preference of the complex to exist in its C_{3v} conformation.

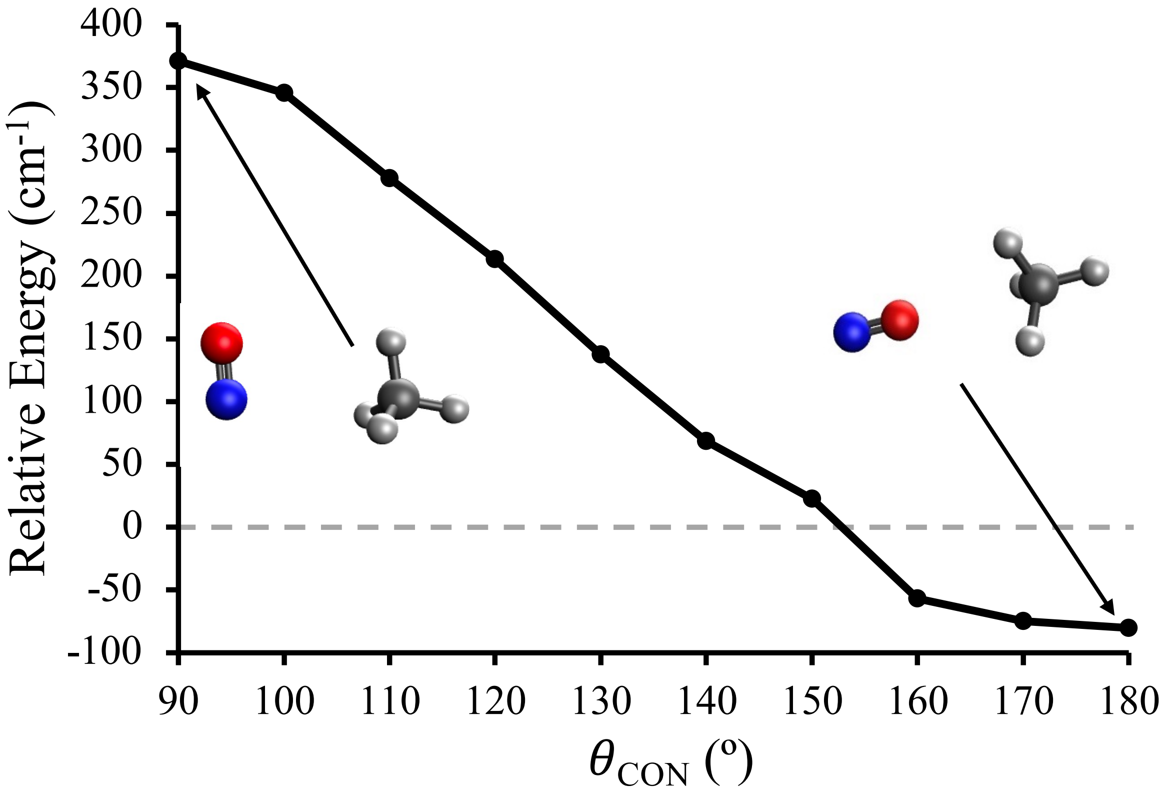

Figure S3. Dependence of the energy of the D_2 state of NO+H_3CH as a function of θ_{CON}, the angle between the carbon atom of CH_4 and the ON, at a fixed intermolecular distance of R_{OC}=3.27 Å. The geometry optimizations were calculated using EOM-EA-CCSD/aug-cc-pVTZ and a three-point extrapolation to the CBS limit was performed for the electronic energies. All energies are reported relative to a D_2-optimized geometry with an intermolecular distance of 20 Å. The energies increase significantly as the θ_{CON} decreases, displaying the preference of the complex to exist in its C_{3v} conformation.

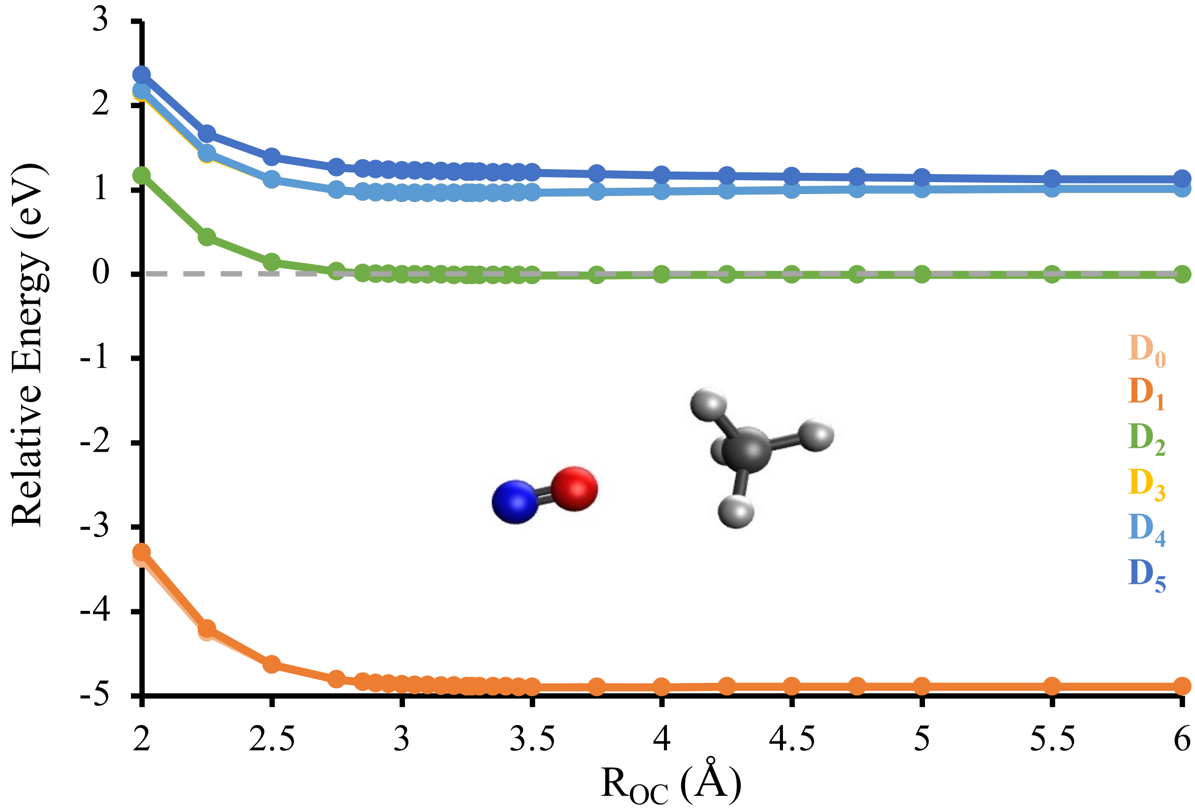

Figure S4. Energies of the D_0 to D_5 states of the NO-H_3CH plotted against R_{OC}, the distance between the oxygen and carbon atoms in the above conformation. The calculations were performed at the EOM-EA-CCSD/AVQZ//EOM-EA-CCSD/aug-cc-pVTZ level of theory, and all energies are reported relative to a D_2-optimized geometry with an intermolecular distance of 20 Å. The AVQZ basis set uses d-aug-cc-pVQZ for the N and O atoms and aug-cc-pVQZ for the C and H atoms.

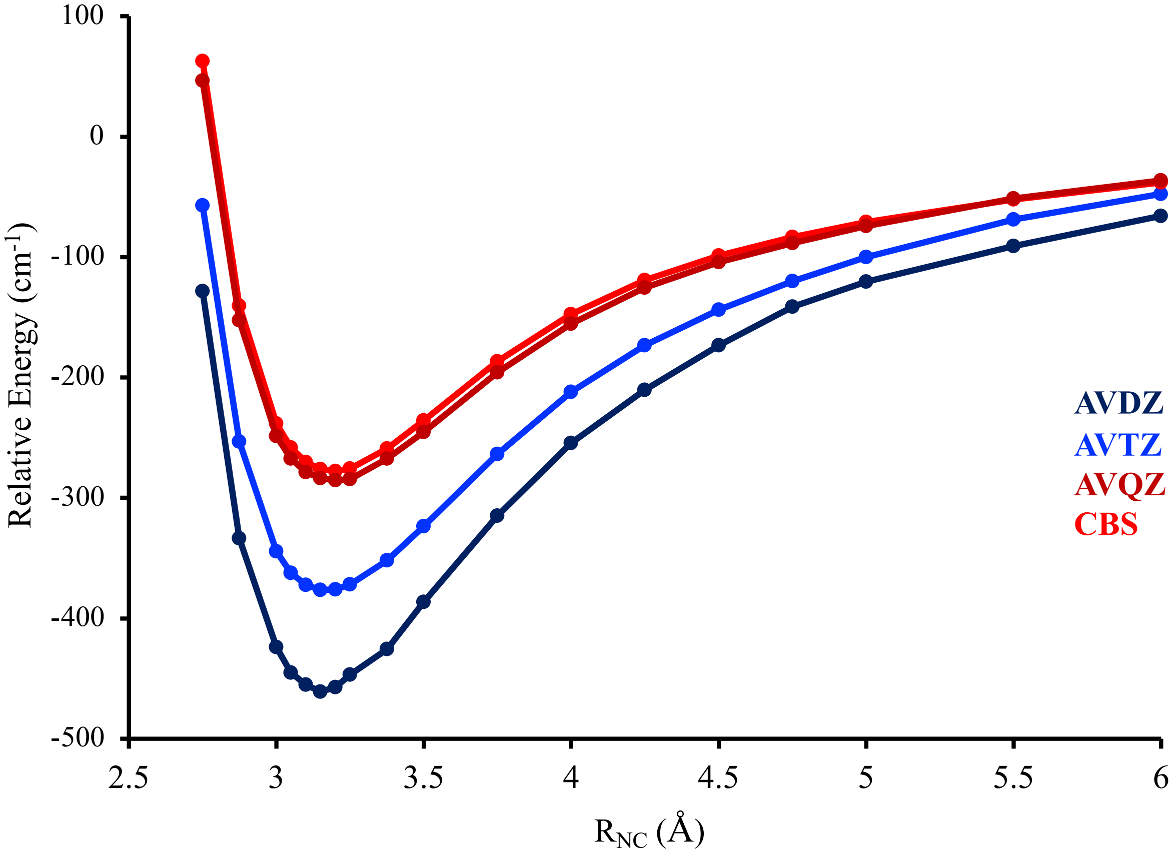

Figure S5. The D_2 energy versus intermolecular distance, R_{NC}, for ON-H_3CH evaluated using a series of different basis sets for the single-point calculations. Here AVNZ means that a d-aug-cc-pVNZ basis set is used for the N and O atoms of NO while an aug-cc-pVNZ basis set is used for the C and H atoms of CH_4. CBS indicates the results of a three-point extrapolation to the complete basis set limit as describe in the text. All energies are reported relative to a D_2-optimized geometry with an intermolecular distance of 20 Å.

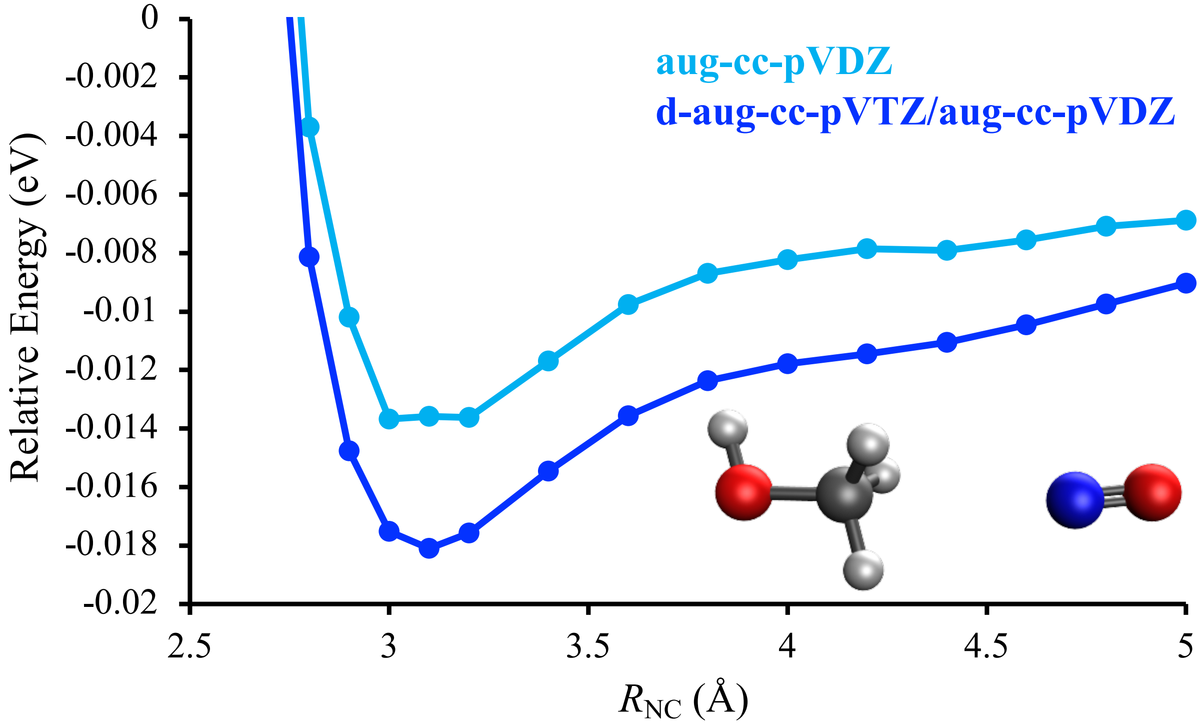

Figure S6. Energy of the D_2 state of ON-H_3COH as a function of R_{NC}, the distance between the nitrogen of NO and carbon of methanol. The geometry optimizations were performed at the EOM-EA-CCSD/aug-cc-pVDZ level of theory (light blue data) or used EOM-EA-CCSD with a d-aug-cc-pVTZ basis set for C, N, and O atoms and an aug-cc-pVDZ basis set for the H atoms (dark blue data). For all geometries, the single-point energies were evaluated at the EOM-EA-CCSD/d-aug-cc-pVTZ level of theory and all energies are reported relative to a D_2-optimized geometry with an intermolecular distance of 20 Å.

Using a larger basis set for the geometry optimizations results in a smoother potential energy curve. This reflects the fact that the intermolecular attractions are very weak at these intermolecular orientations.

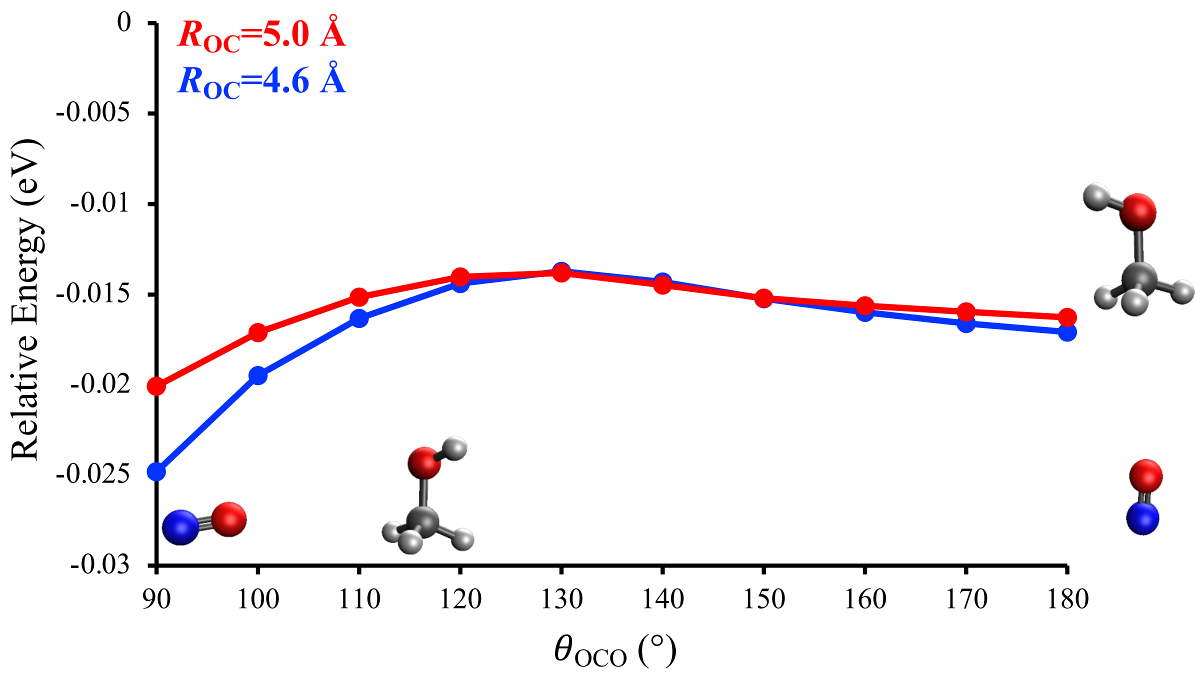

Figure S7. Energy of the D_2 state of NO-H_3COH as a function of the O-C-O angle (θ_{OCO}) at fixed intermolecular distances R_{OC}=5.0 Å (red data) and R_{OC}=4.6 Å (blue data). The geometry optimizations were performed using EOM-EA-CCSD with a d-aug-cc-pVTZ basis set for the C, N, and O atoms and an aug-cc-pVDZ basis set for the H atoms. The single-point energies were evaluated at the EOM-EA-CCSD/d-aug-cc-pVTZ level of theory and all energies are reported relative to a D_2-optimized geometry with an intermolecular distance of 20 Å.

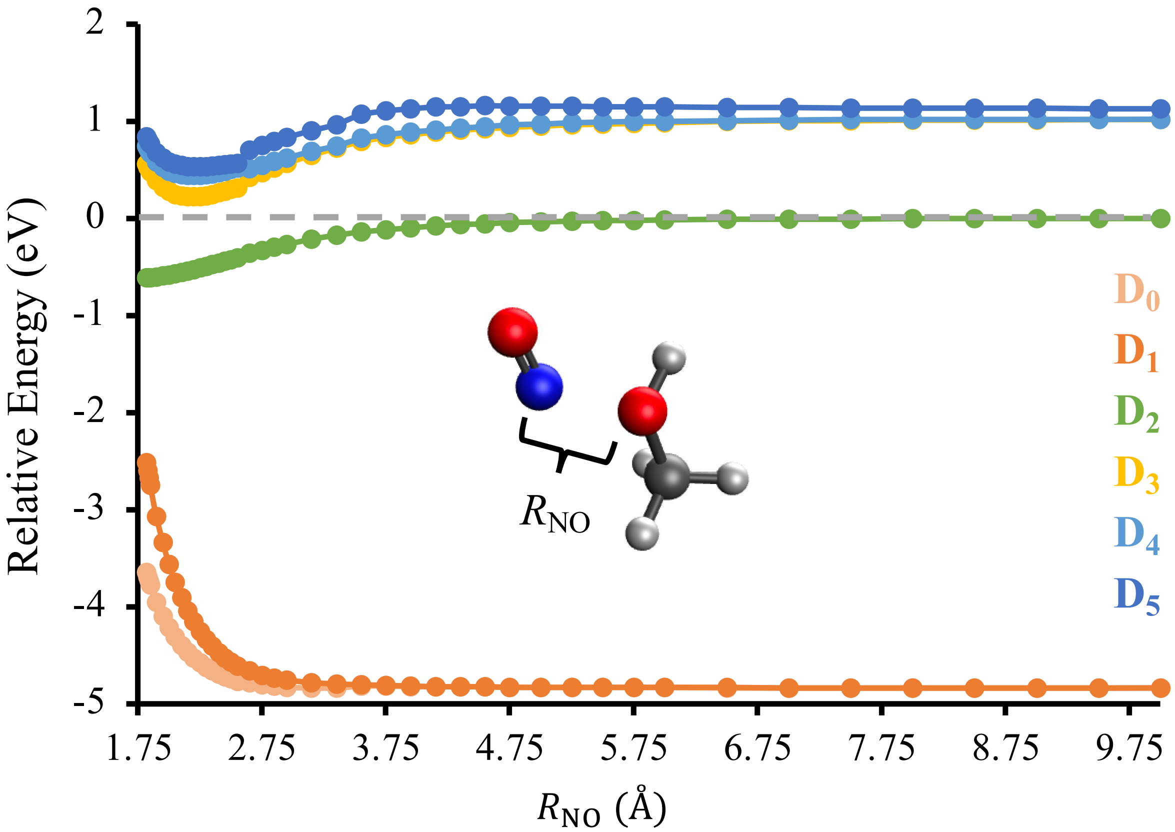

Figure S8. The energy of the D_0 (light orange), D_1 (orange), D_2 (green), D_3 (yellow), D_4 (light blue), and D_5 (blue) states as a function of the intermolecular distance, R_{NO}, when the N of NO is interacting with O of CH_3OH. The geometry optimizations were performed using EOM-EA-CCSD with a d-aug-cc-pVTZ basis set for the C, N, and O atoms and an aug-cc-pVDZ basis set for the H atoms. The single-point energies were evaluated at the EOM-EA-CCSD/d-aug-cc-pVTZ level of theory and all energies are reported relative to a D_2-optimized geometry with an intermolecular distance of 20 Å.

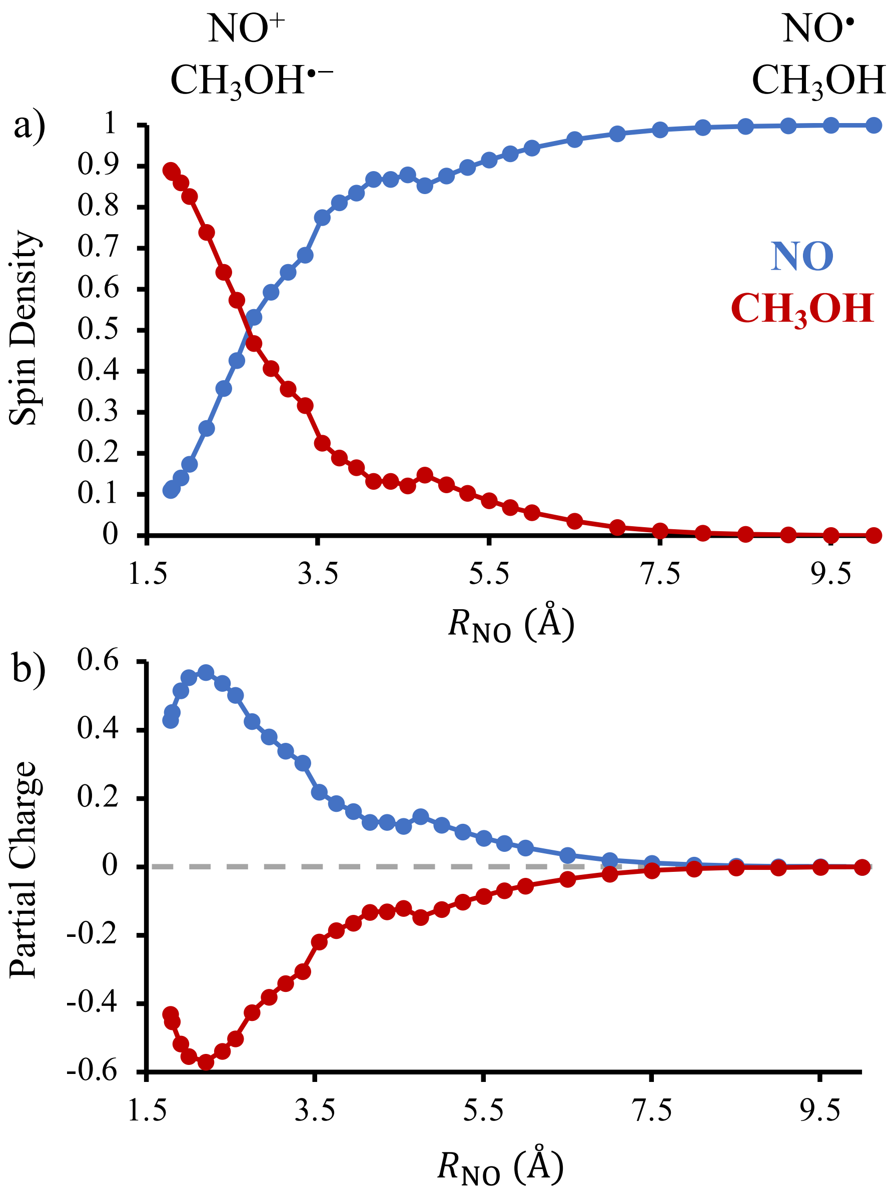

Figure S9. Panel a) shows the total Löwdin spin density on NO (blue) and CH_3OH (red) of the D_2 state as a function of the intermolecular distance, R_{NO}, when the NO is interacting with the O atom of CH_3OH. Panel b) shows the total Löwdin partial charges on the NO and CH_3OH of the D_2 state as a function of the intermolecular distance. The total spin density on a molecule is calculated by summing together the Löwdin spin densities of each atom belonging to that molecule. A similar analysis is done for the total partial charges. The geometry optimizations were performed using EOM-EA-CCSD with a d-aug-cc-pVTZ basis set for the C, N, and O atoms and an aug-cc-pVDZ basis set for the H atoms. The population analysis was performed at the EOM-EA-CCSD/aug-cc-pVTZ level of theory.

Note that this analysis supports the harpoon mechanism by showing that electron transfer occurs from NO ($A^2\Sigma^+$) to CH_3OH. This creates a transient NO^++CH_3OH^- ion pair, increasing the intermolecular attractions between the two molecules.

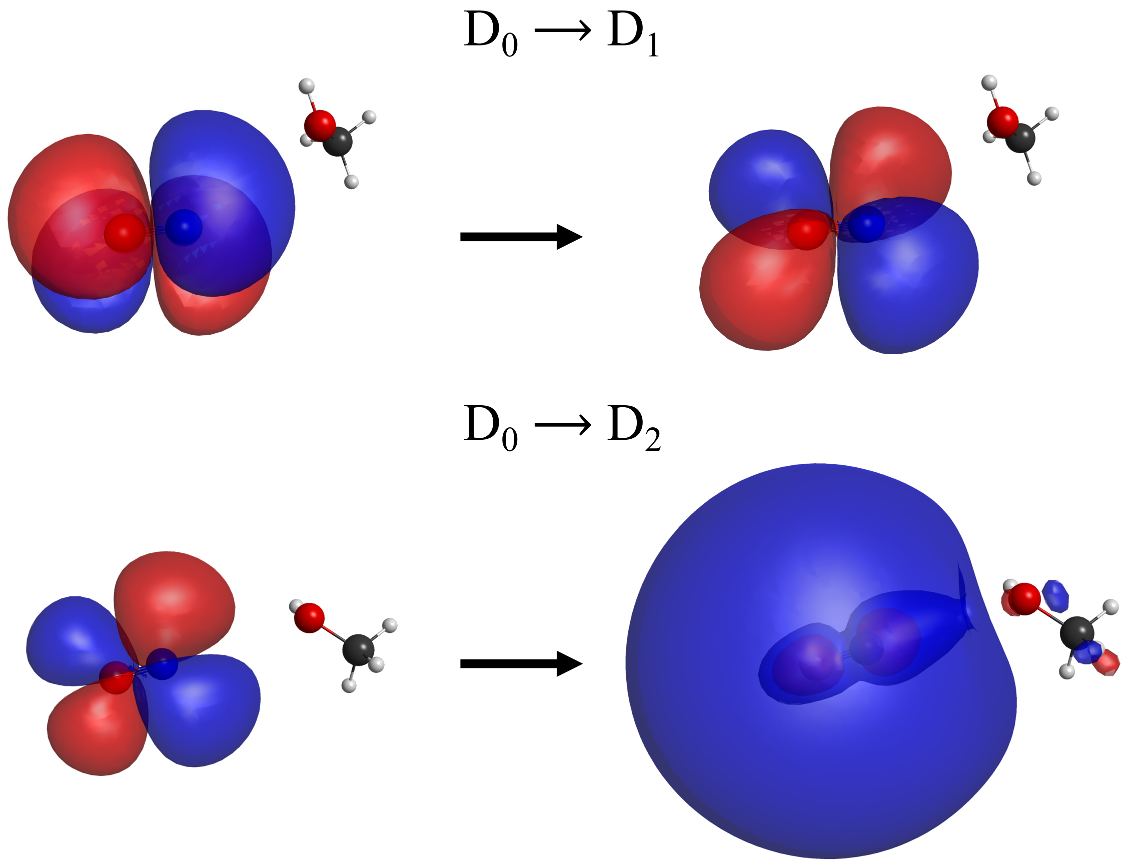

Figure S10: Natural transition orbitals for the $D_0 \rightarrow D_1$ and $D_0 \rightarrow D_2$ transition of ON+CH_3OH evaluated at a geometry with R_{NO} = 3.95 Å. The geometry optimizations were performed using EOM-EA-CCSD with a d-aug-cc-pVTZ basis set for the C, N, and O atoms and an aug-cc-pVDZ basis set for the H atoms. The natural transition orbital analysis was performed at the EOM-EA-CCSD/aug-cc-pVTZ level of theory. Note that the orientation is different for the two transitions. The orbitals were plotted using wxMacMolPlt with 150 grid points and a contour value of 0.01.

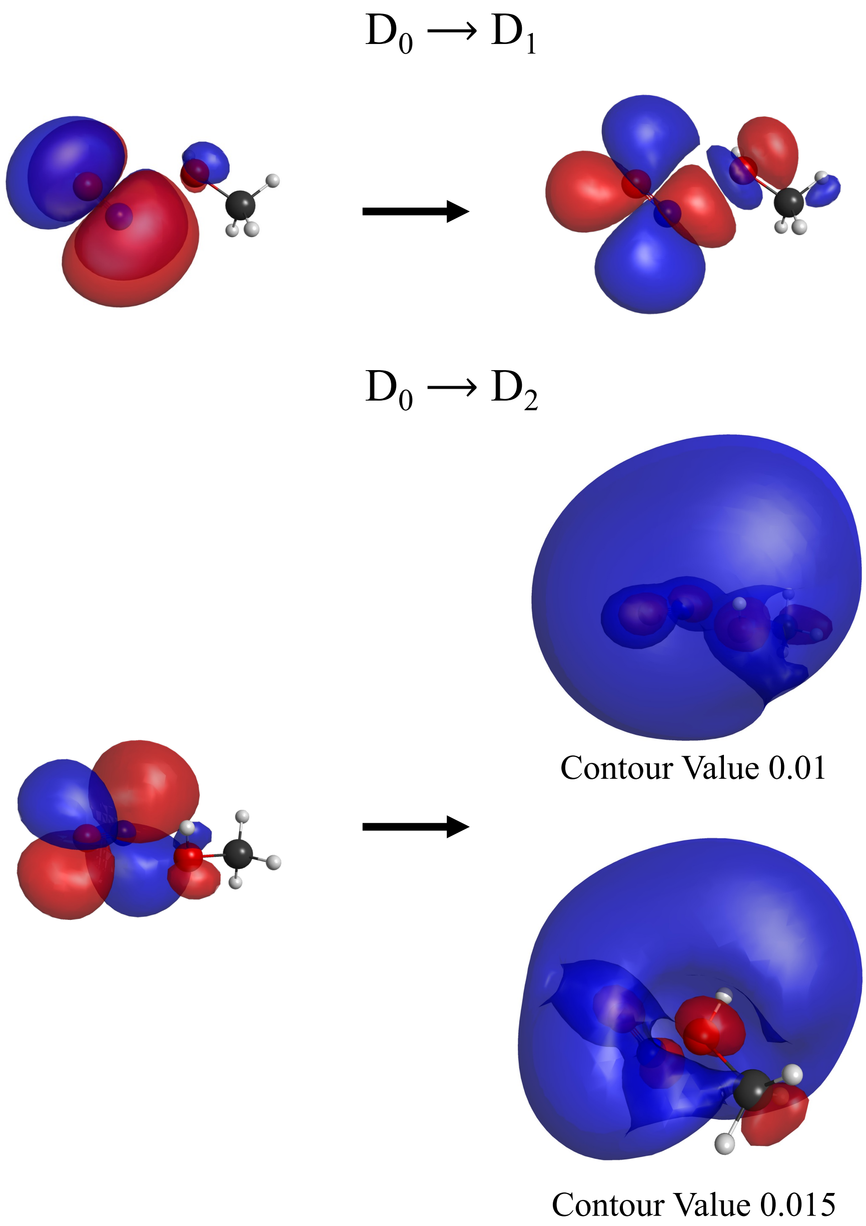

Figure S11: Natural transition orbitals for the $D_0 \rightarrow D_1$ and $D_0 \rightarrow D_2$ transition of ON+CH_3OH evaluated at a geometry with R_{NO} = 2.20 Å. The geometry optimizations were performed using EOM-EA-CCSD with a d-aug-cc-pVTZ basis set for the C, N, and O atoms and an aug-cc-pVDZ basis set for the H atoms. The natural transition orbital analysis was performed at the EOM-EA-CCSD/aug-cc-pVTZ level of theory. Note that the orientation is different for the two transitions. The orbitals were plotted using wxMacMolPlt with 150 grid points and a contour value of 0.01. The LUNTO for the $D_0 \rightarrow D_2$ transition was also plotted with a contour value of 0.015 to highlight the development of electron density on the CH_3OH.

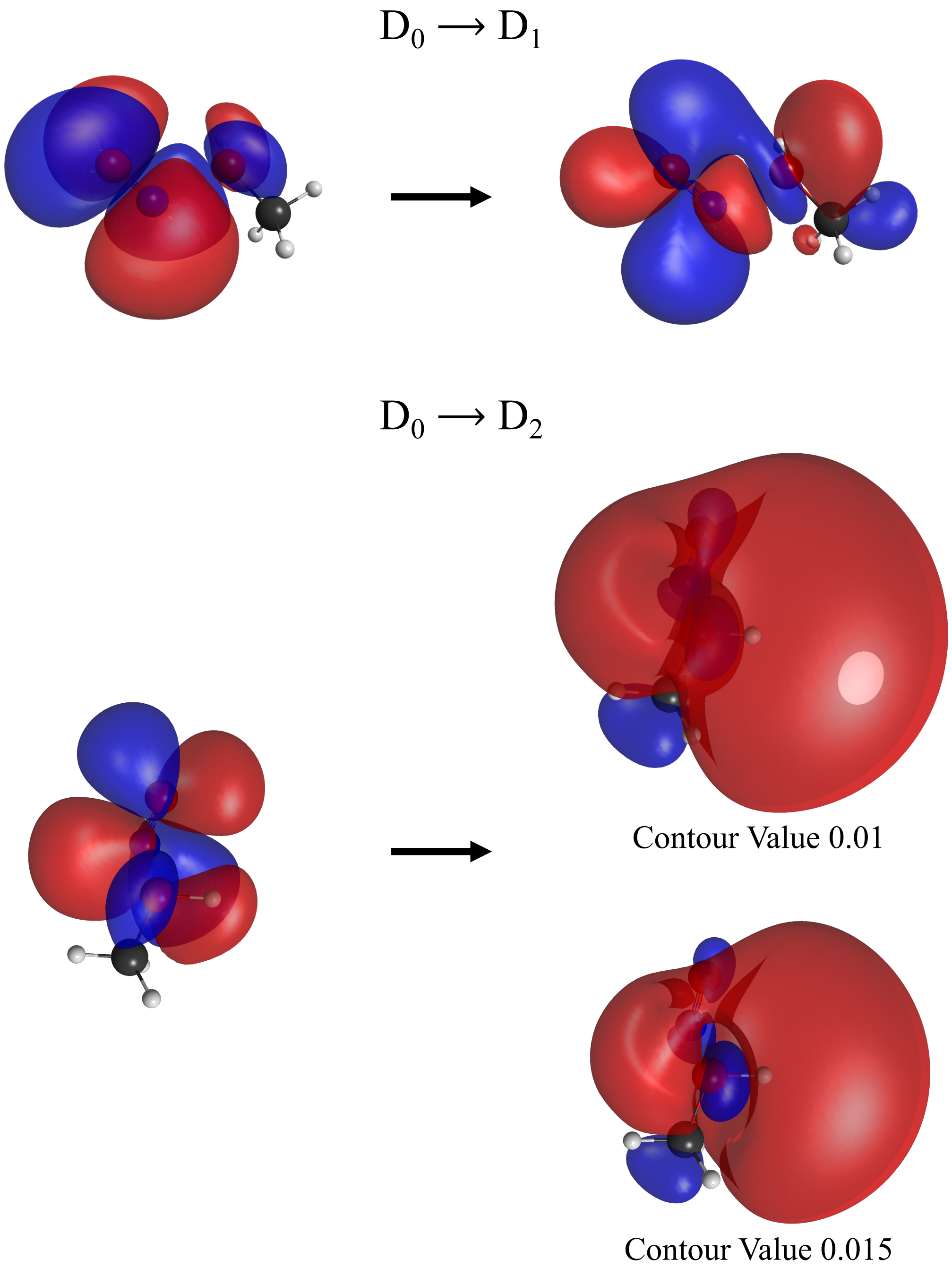

Figure S12: Natural transition orbitals for the $D_0 \rightarrow D_1$ and $D_0 \rightarrow D_2$ transition of NO+CH_3OH evaluated at a geometry with R_{NO} = 1.78 Å. The geometry optimizations were performed using EOM-EA-CCSD with a d-aug-cc-pVTZ basis set for the C, N, and O atoms and an aug-cc-pVDZ basis set for the H atoms. The natural transition orbital analysis was performed at the EOM-EA-CCSD/aug-cc-pVTZ level of theory. Note that the orientation is different for the two transitions. The orbitals were plotted using wxMacMolPlt with 150 grid points and a contour value of 0.01. The LUNTO for the $D_0 \rightarrow D_2$ transition was also plotted with a contour value of 0.015.
Note that the SOMO of the D_2 state has significant amplitude on the OH group of CH_3OH. The $D_0 \rightarrow D_2$ transition therefore exhibits significant charge-transfer character which is consistent with the harpoon mechanism.

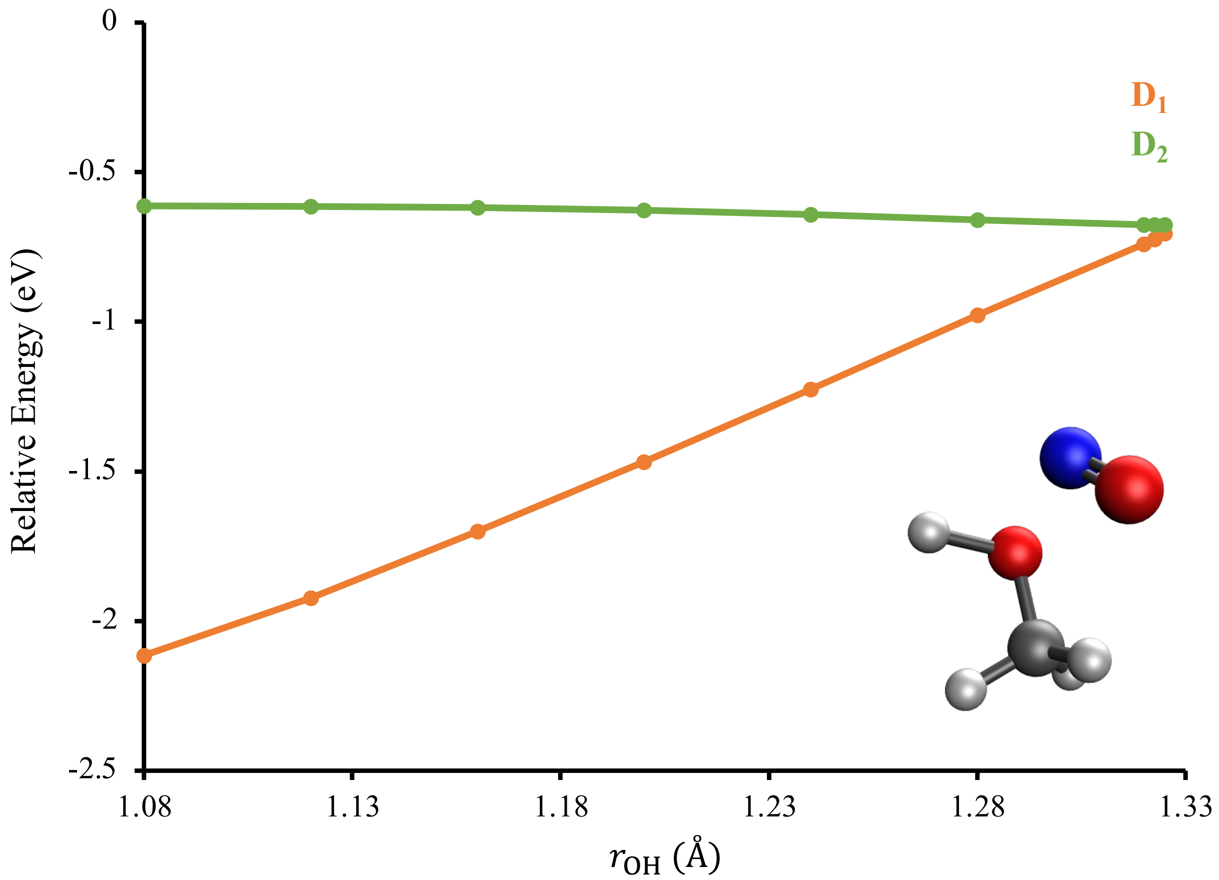

Figure S13: The energy of the D_1 and D_2 states as a function of the O-H bond length (r_{OH}) at a fixed intermolecular distance of R_{NO}=1.78 Å. The geometry optimizations were performed using EOM-EA-CCSD with a d-aug-cc-pVTZ basis set for the C, N, and O atoms and an aug-cc-pVDZ basis set for the H atoms. The single-point energies were evaluated at the EOM-EA-CCSD/d-aug-cc-pVTZ level of theory and all energies are reported relative to a D_2-optimized geometry with an intermolecular distance of 20 Å. The only constraints included in these calculations were the R_{NO} and r_{OH} bond lengths; the calculations were initialized from geometries with the NO oriented above the methyl group.

Note that this pathway to a D_2-D_1 conical intersection is nearly identical to that shown in Figure 5b.

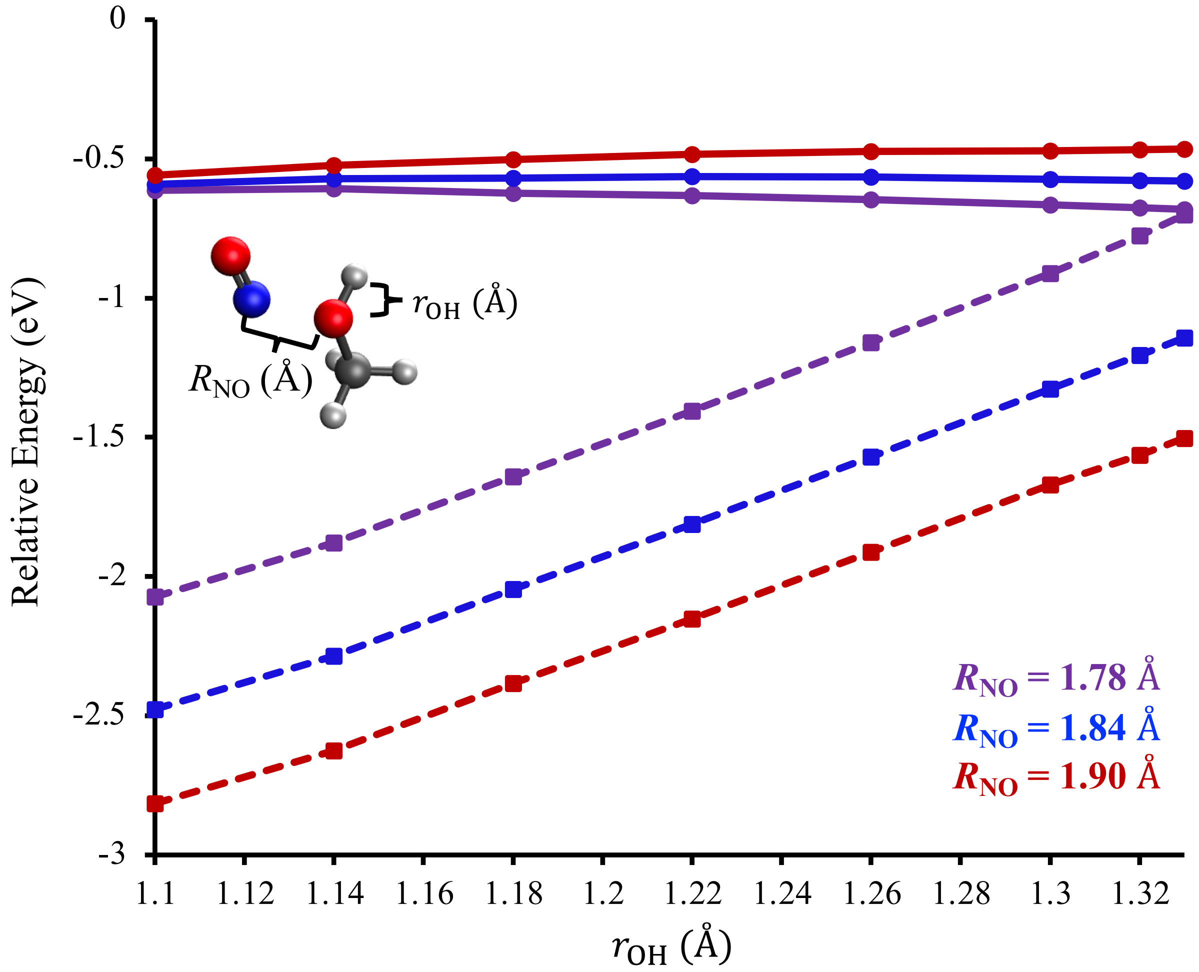

Figure S14: The energy of the D_1 (dashed lines, square points) and D_2 (solid lines, circle points) states as a function of the O-H bond length (r_{OH}) at a fixed intermolecular distance of R_{NO}=1.78 Å (purple data), R_{NO}=1.84 Å (blue data), and R_{NO}=1.90 Å (red data). The geometry optimizations were performed using EOM-EA-CCSD with a d-aug-cc-pVTZ basis set for the C, N, and O atoms and an aug-cc-pVDZ basis set for the H atoms. The single-point energies were evaluated at the EOM-EA-CCSD/d-aug-cc-pVTZ level of theory and all energies are reported relative to a D_2-optimized geometry with an intermolecular distance of 20 Å.

Note that this data demonstrates that the pathway to the D_2-D_1 conical intersection requires both decreasing R_{NO} and increasing r_{OH}.

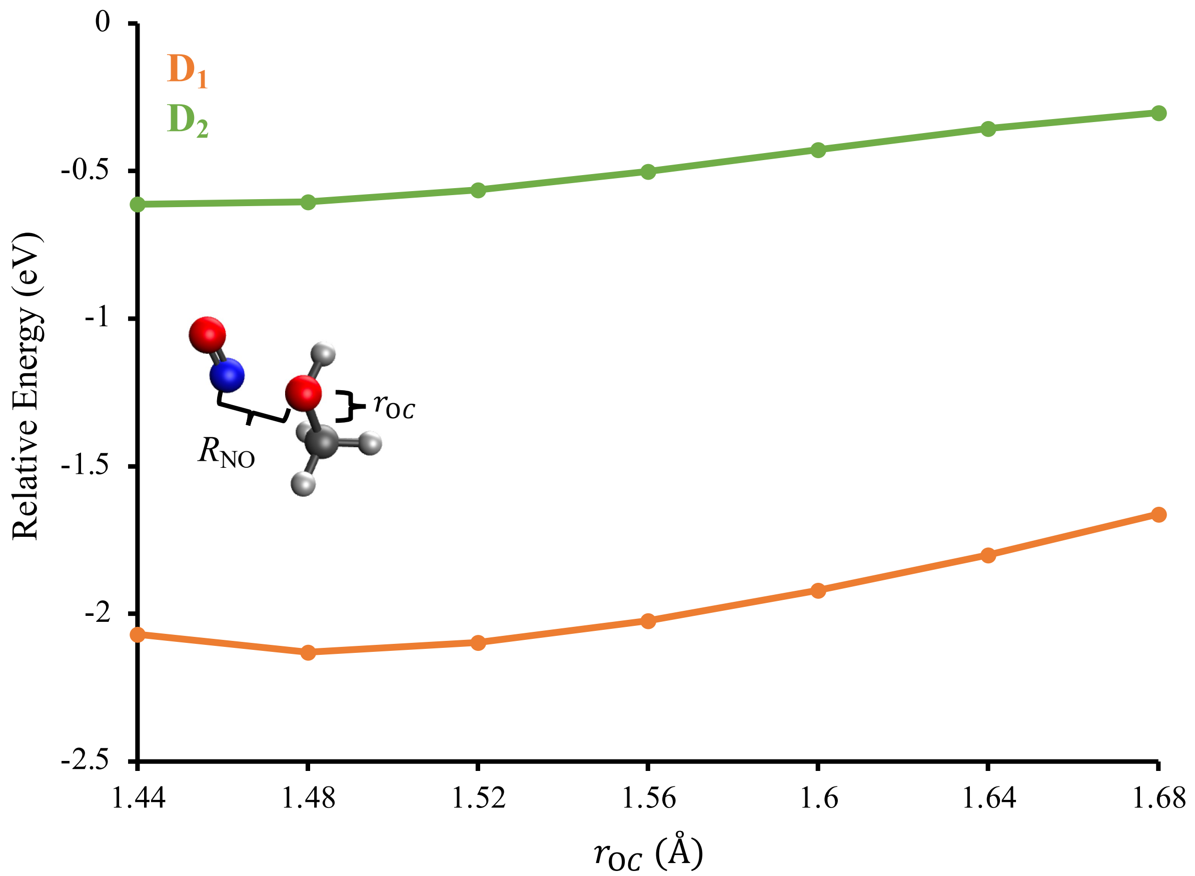

Figure S15: The energy of the D_1 and D_2 states as a function of the O-C bond length (r_{OC}) at a fixed intermolecular distance of R_{NO}=1.78 Å. The geometry optimizations were performed using EOM-EA-CCSD with a d-aug-cc-pVTZ basis set for the C, N, and O atoms and an aug-cc-pVDZ basis set for the H atoms. The single-point energies were evaluated at the EOM-EA-CCSD/d-aug-cc-pVTZ level of theory and all energies are reported relative to a D_2-optimized geometry with an intermolecular distance of 20 Å.

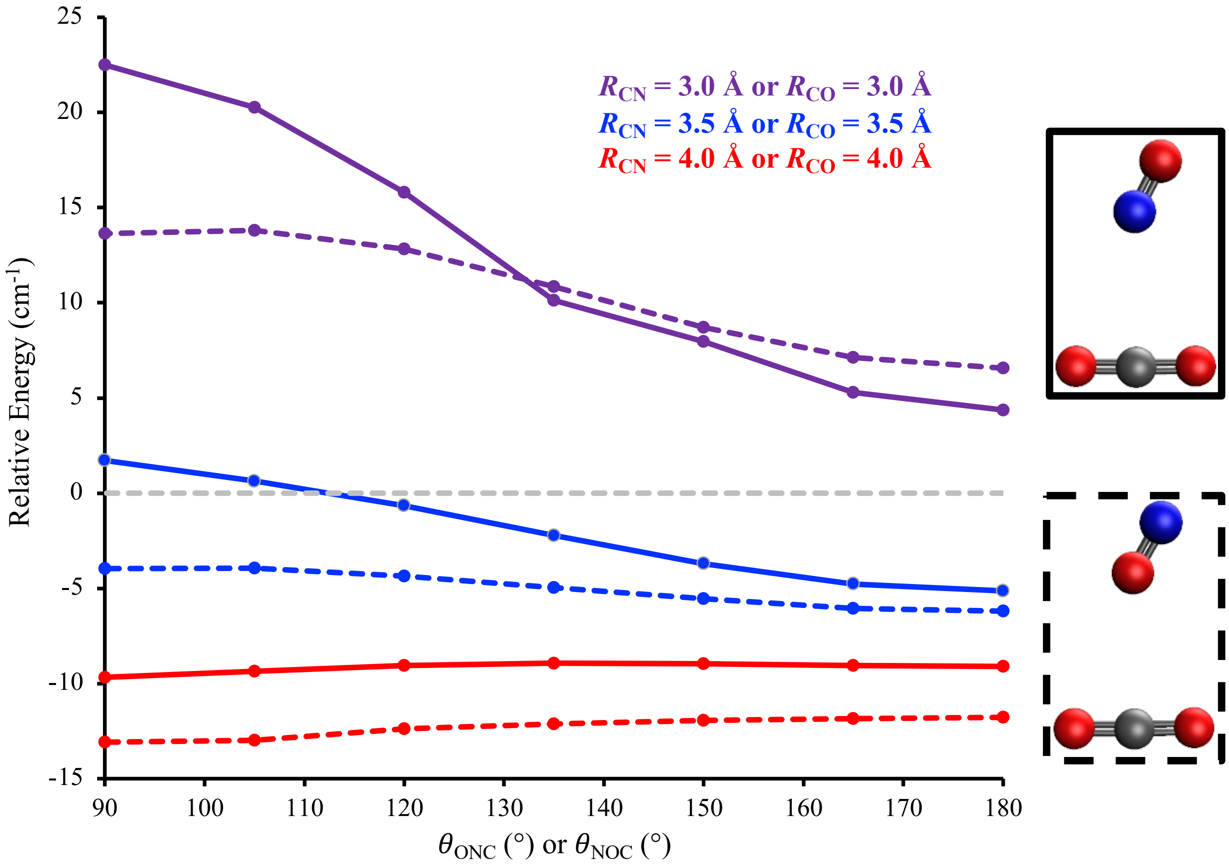

Figure S16: Energies of the D_2 state as a function of θ_{ONC} (solid lines) or θ_{NOC} (dashed lines). As illustrated by the molecular structures, all conformations have the NO interacting directly with the carbon atom of the CO_2; the θ_{NCO} or θ_{OCO} angle was also constrained to 90°. The colors denote the constrained intermolecular distance. The calculations were performed at the EOM-EA-CCSD/AVQZ//EOM-EA-CCSD/aug-cc-pVTZ level of theory and all energies are reported relative to a D_2-optimized geometry with an intermolecular distance of 20 Å. The AVQZ basis set consists of d-aug-cc-pVQZ for the N and O atoms of NO and aug-cc-pVQZ for the C and O atoms of CO_2.

Note that the intermolecular interactions become increasingly repulsive as the intermolecular distance is reduced. This suggests that interactions between the NO and the carbon atom of CO_2 are unfavorable for the D_2 state.

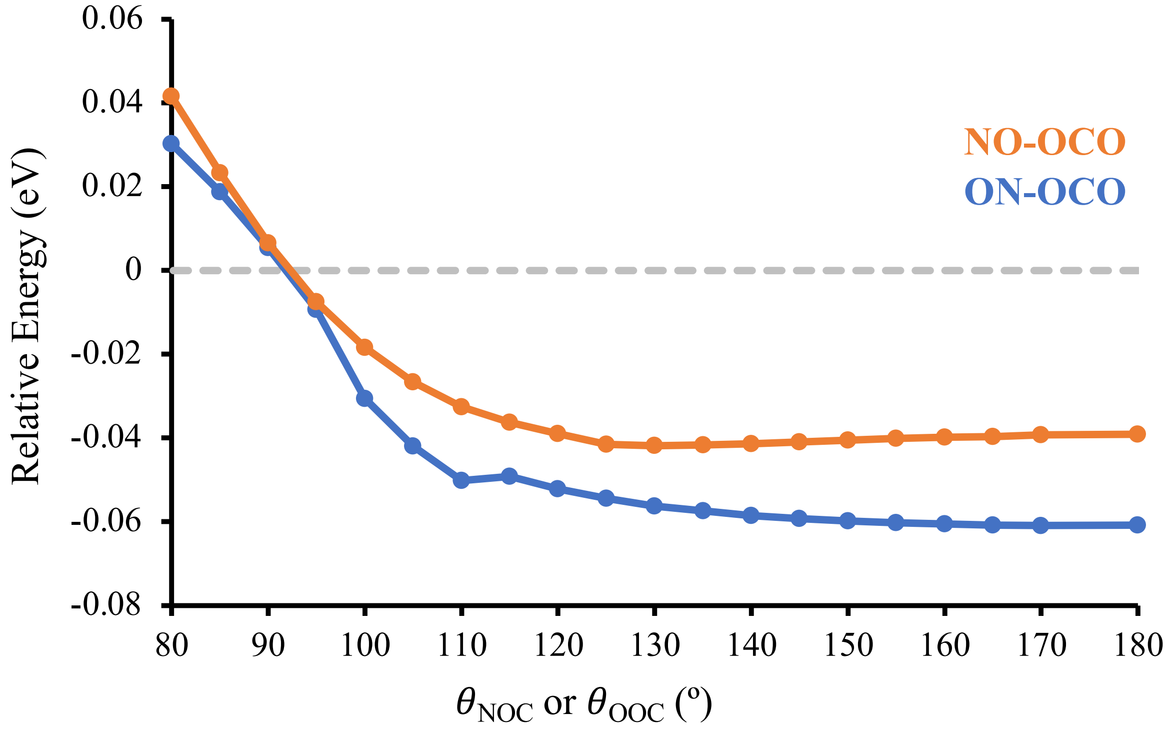

Figure S17: Energies for the D_2 states of two different molecular orientations, NO-OCO and ON-OCO, as a function of the intermolecular angle, θ_{OOC} or θ_{NOC}, respectively. The intermolecular distance R_{NO} or R_{OO} is constrained to 3.3 Å. Throughout the various angle changes, the NO-OCO confirmation is higher in energy. The maximum difference in energy is 0.02 eV. The calculations were performed at the EOM-EA-CCSD/AVQZ//EOM-EA-CCSD/aug-cc-pVTZ level of theory. The AVQZ basis set consists of d-aug-cc-pVQZ for the N and O atoms of NO and aug-cc-pVQZ for the C and O atoms of CO_2.

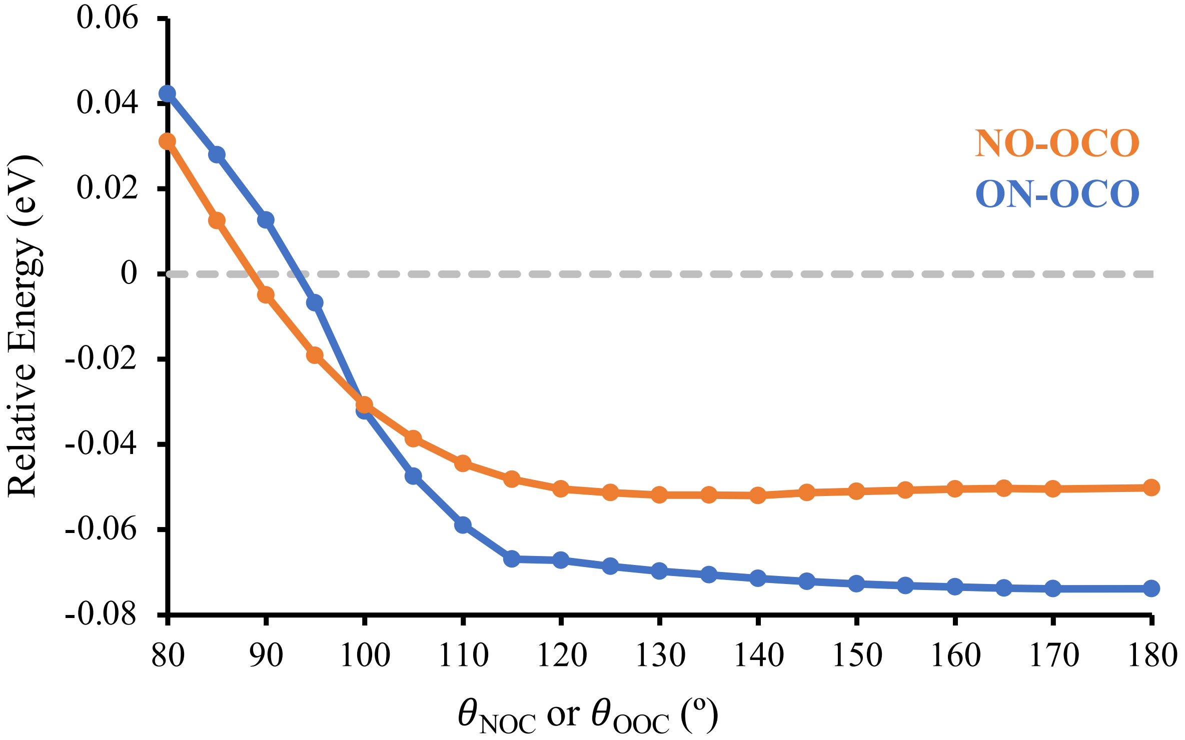

Figure S18: Energies for the D_2 states of two different molecular orientations, NO-OCO and ON-OCO, as a function of the intermolecular angle, θ_{OOC} or θ_{NOC}, respectively. The intermolecular distance R_{NO} or R_{OO} is constrained to 3.1 Å. Throughout the various angle changes, the NO-OCO confirmation is higher in energy. The maximum difference in energy is 0.02 eV. The calculations were performed at the EOM-EA-CCSD/AVQZ//EOM-EA-CCSD/aug-cc-pVTZ level of theory. The AVQZ basis set consists of d-aug-cc-pVQZ for the N and O atoms of NO and aug-cc-pVQZ for the C and O atoms of CO_2.

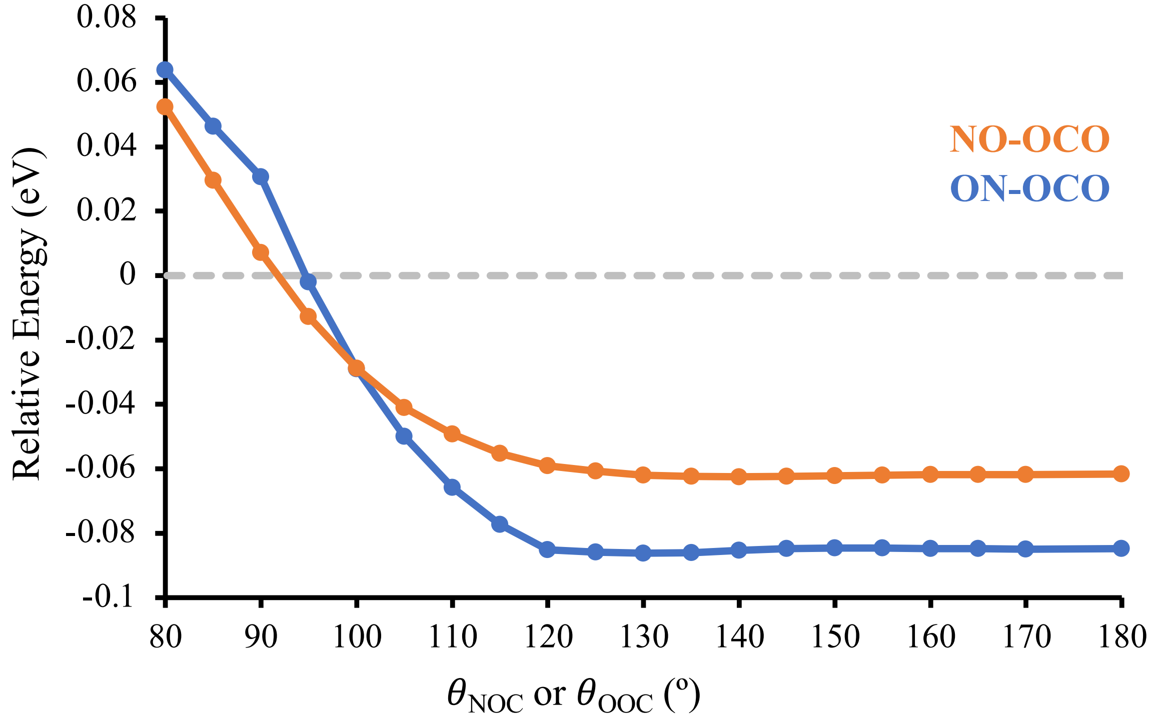

Figure S19: Energies for the D_2 states of two different molecular orientations, NO-OCO and ON-OCO, as a function of the intermolecular angle, θ_{OOC} or θ_{NOC}, respectively. The intermolecular distance R_{NO} or R_{OO} is constrained to 2.9 Å. Throughout the various angle changes, the NO-OCO confirmation is higher in energy. The maximum difference in energy is 0.02 eV. The calculations were performed at the EOM-EA-CCSD/AVQZ//EOM-EA-CCSD/aug-cc-pVTZ level of theory. The AVQZ basis set consists of d-aug-cc-pVQZ for the N and O atoms of NO and aug-cc-pVQZ for the C and O atoms of CO_2.

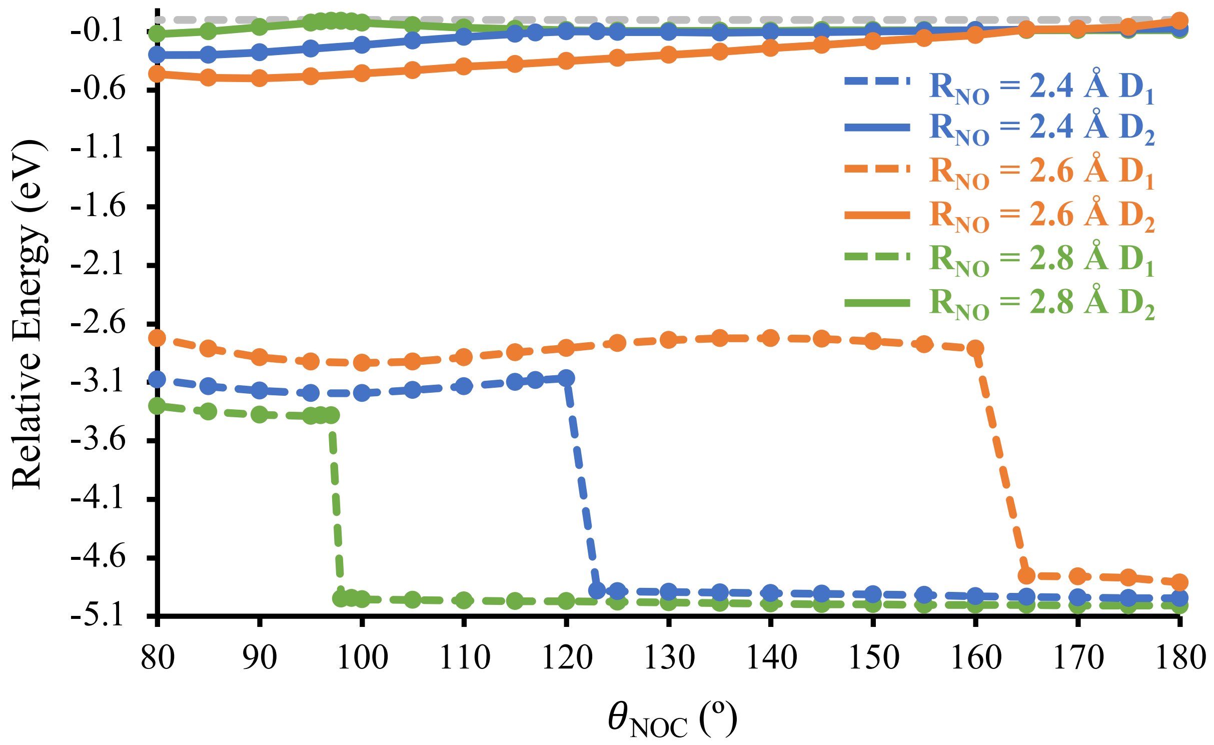

Figure S20: The energy of the D_1 (dashed lines) and D_2 (solid lines) states of ON+OCO as a function of the intermolecular angle θ_{NOC} at the intermolecular distances R_{NO}=2.4 Å (blue), R_{NO}=2.6 Å (orange), and R_{NO}=2.8 Å (green). This data was calculated at the EOM-EA-CCSD/AVQZ//EOM-EA-CCSD/aug-cc-pVDZ level of theory. All energies are reported relative to a D_2-optimized geometry with an intermolecular distance of 20 Å. The AVQZ basis set consists of d-aug-cc-pVQZ for the N and O atoms of NO and aug-cc-pVQZ for the C and O atoms of CO_2.

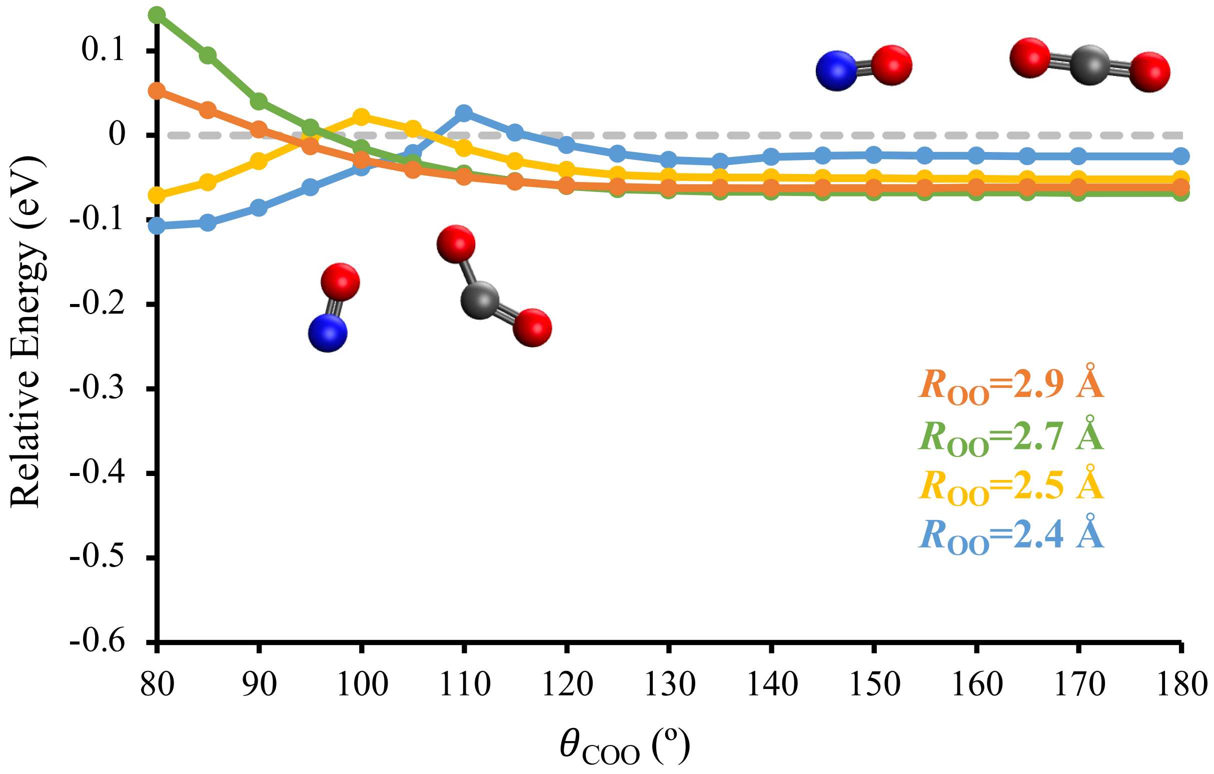

Figure S21. The energy of the D_2 state of NO+OCO as a function of the intermolecular angle θ_{OOC} at the intermolecular distances R_{OO}=2.9 Å (blue), R_{ON}=2.7 Å (orange), R_{ON}=2.5 Å (green), and R_{ON}=2.4 Å (yellow). This data was calculated at the EOM-EA-CCSD/AVQZ//EOM-EA-CCSD/aug-cc-pVDZ level of theory. All energies are reported relative to a D_2-optimized geometry with an intermolecular distance of 20 Å. The AVQZ basis set consists of d-aug-cc-pVQZ for the N and O atoms of NO and aug-cc-pVQZ for the C and O atoms of CO_2.

Note that the range of the y-axis below 0 is identical to that shown in Figure 7 to highlight the observation that the intermolecular attractions are much weaker for NO+OCO conformations than for ON+OCO conformations.

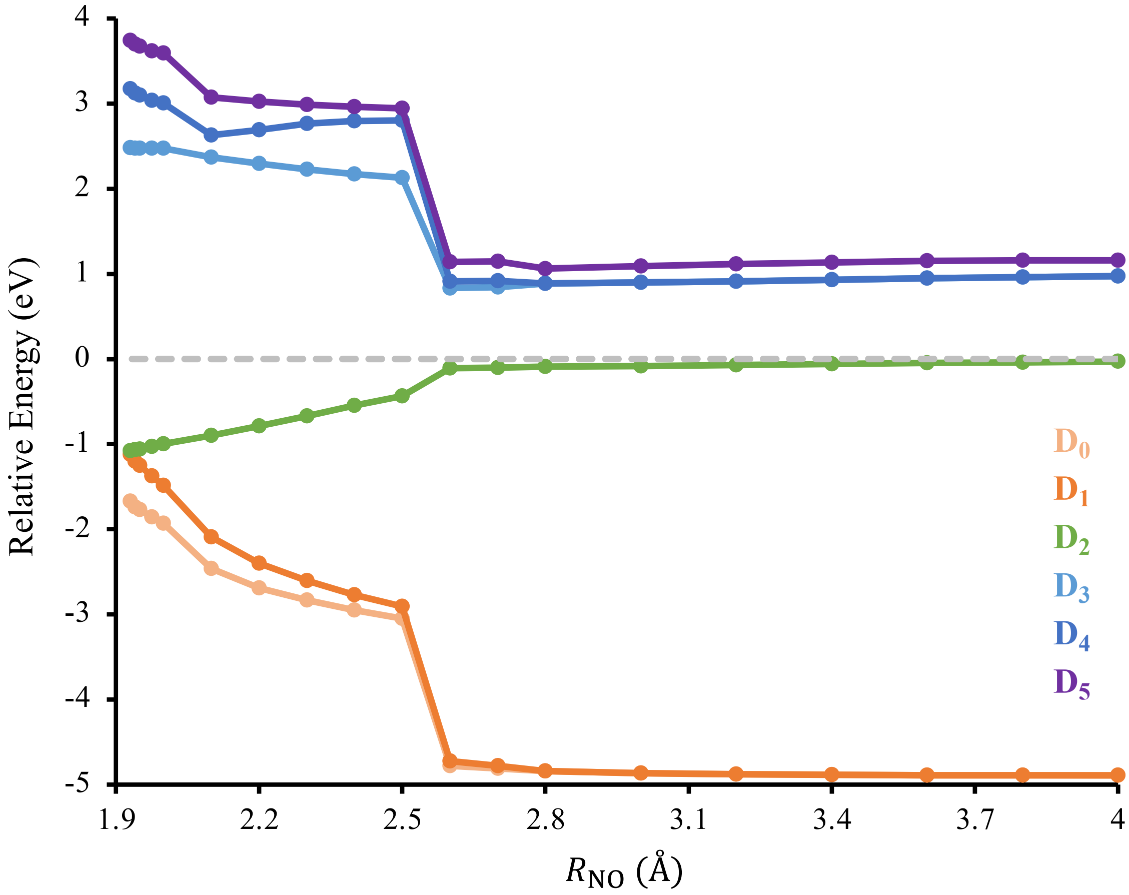

Figure S22. The energy of the D_0 (light orange), D_1 (orange), D_2 (green), D_3 (light blue), D_4 (blue), and D_5 (purple) states as a function of the intermolecular distance, R_{NO}, when the N of NO is interacting with an O of CO_2. The geometries are the same as those analyzed in Figure 10. The geometry optimizations were performed at the EOM-EA-CCSD/AVQZ//EOM-EA-CCSD/aug-cc-pVTZ level of theory and all energies are reported relative to a D_2-optimized geometry with an intermolecular distance of 20 Å. The AVQZ basis set consists of d-aug-cc-pVQZ for the N and O atoms of NO and aug-cc-pVQZ for the C and O atoms of CO_2.

Note that the D_3-D_5 states remain well-separated energetically from D_2 throughout this pathway.

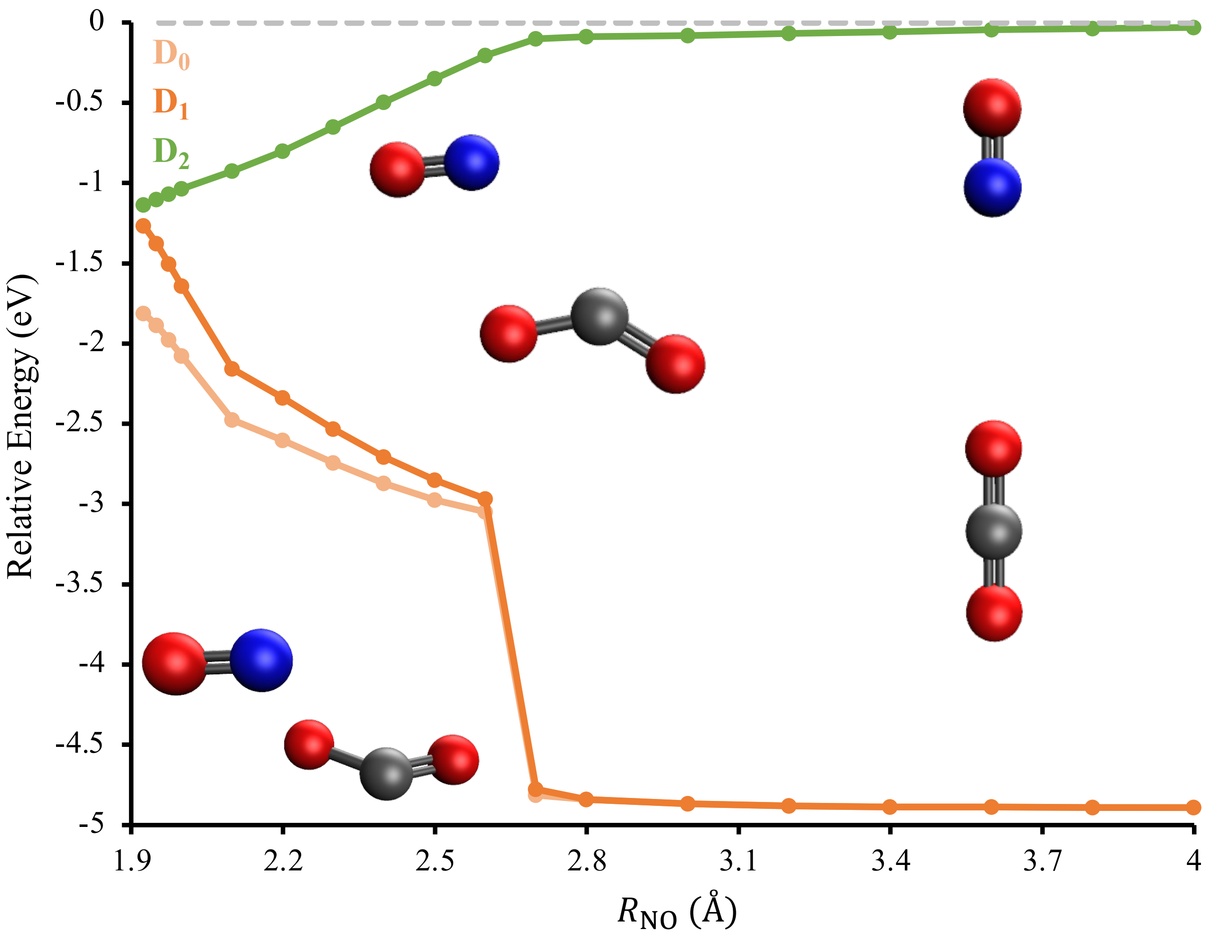

Figure S23: The energy of the D_0 (light orange), D_1 (orange), and D_2 (green) states as a function of the intermolecular distance, R_{NO}, when the N of NO is interacting with an O of CO_2. The insets show representative molecular geometries along this pathway. The geometry optimizations were performed at the EOM-EA-CCSD/AVQZ//EOM-EA-CCSD/aug-cc-pVTZ level of theory and all energies are reported relative to a D_2-optimized geometry with an intermolecular distance of 20 Å. The AVQZ basis set consists of d-aug-cc-pVQZ for the N and O atoms of NO and aug-cc-pVQZ for the C and O atoms of CO_2.

Note that, the conformations here are distinct from those in Figure 10 beginning at R_{NO}=2.7 Å. At R_{NO}=2.6 Å, the system isomerizes into a planar conformation with a bent CO_2 and the O atom of the NO oriented away from the CO_2. The D_2 potential is strongly attractive after this, leading to a D_2-D_1 conical intersection at approximately R_{NO}=1.925 Å and $E_{D_2} = -1.14$ eV. Along this pathway, the complex becomes non-planar beginning at R_{NO}=2.0 Å.

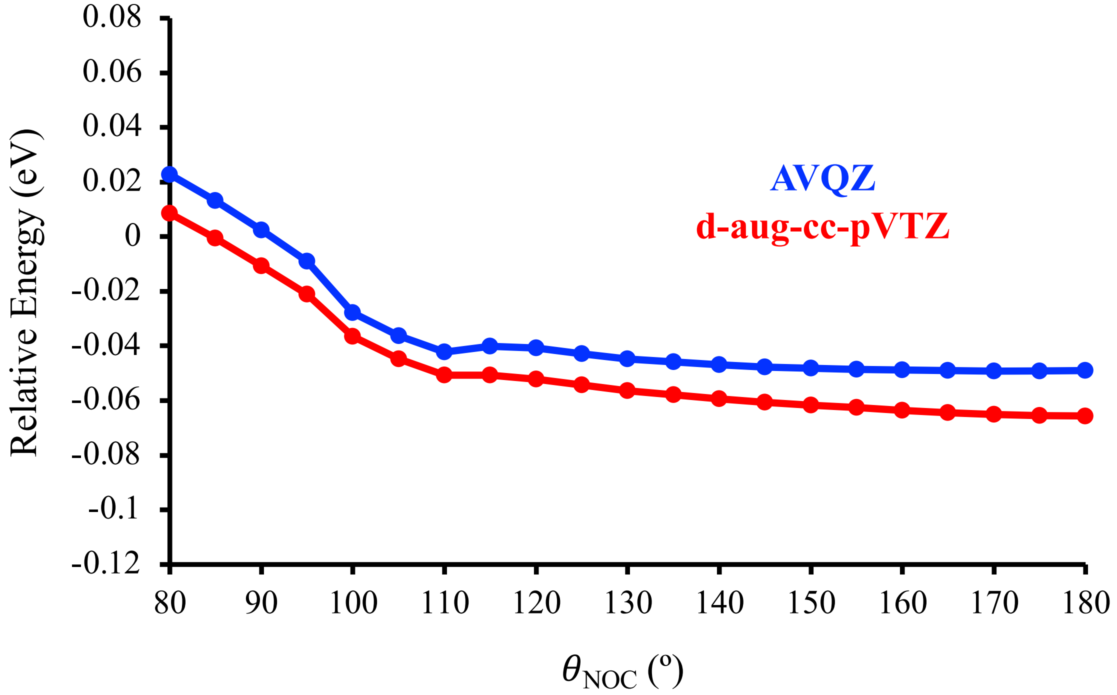

Figure S24: Energies for the D_2 states of ON-OCO as a function of the intermolecular angle, θ_{NOC} at a fixed intermolecular distance of R_{NO}=3.5 Å. The single-point energies were evaluated using a d-aug-cc-pVTZ basis set (red data points) or an AVQZ basis set (blue data points).

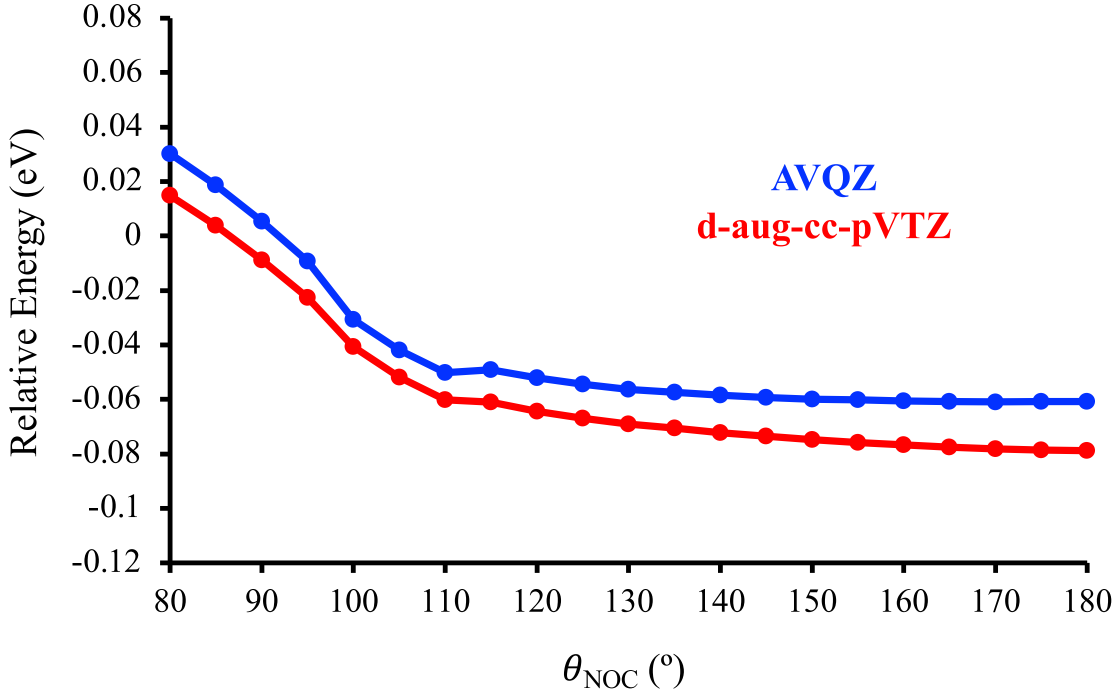

Figure S25: Energies for the D_2 states of ON-OCO as a function of the intermolecular angle, θ_{NOC} at a fixed intermolecular distance of R_{NO}=3.3 Å. The single-point energies were evaluated using a d-aug-cc-pVTZ basis set (red data points) or an AVQZ basis set (blue data points).

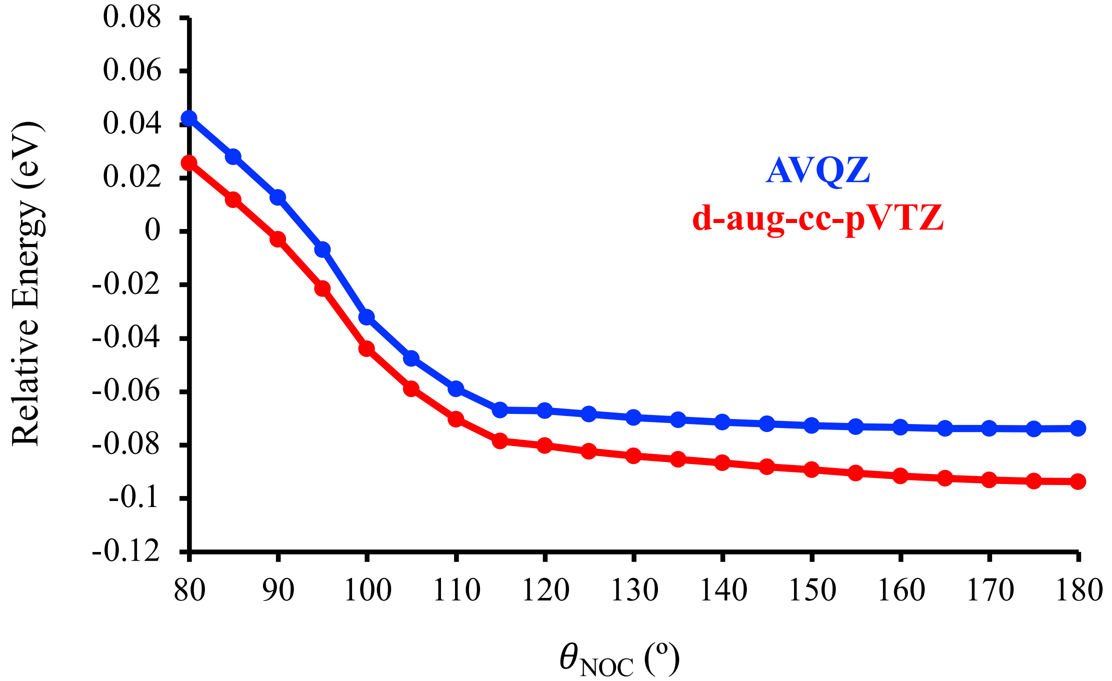

Figure S26: Energies for the D_2 states of ON-OCO as a function of the intermolecular angle, θ_{NOC} at a fixed intermolecular distance of R_{NO}=3.1 Å. The single-point energies were evaluated using a d-aug-cc-pVTZ basis set (red data points) or an AVQZ basis set (blue data points).

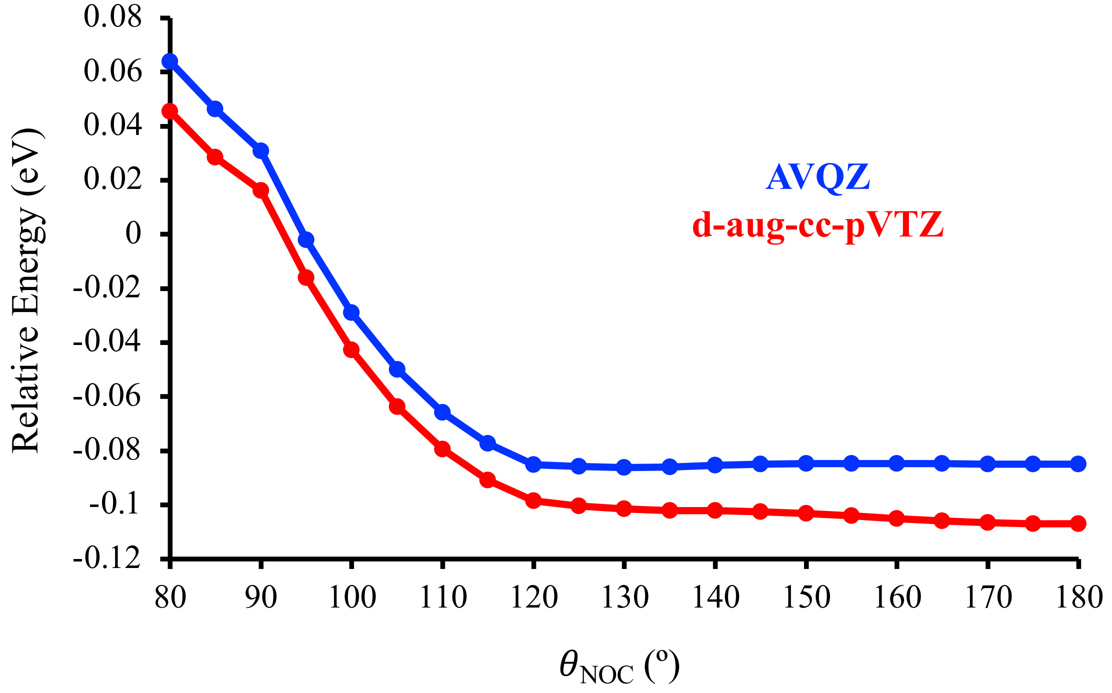

Figure S27: Energies for the D_2 states of ON-OCO as a function of the intermolecular angle, θ_{NOC} at a fixed intermolecular distance of R_{NO}=2.9 Å. The single-point energies were evaluated using a d-aug-cc-pVTZ basis set (red data points) or an AVQZ basis set (blue data points).

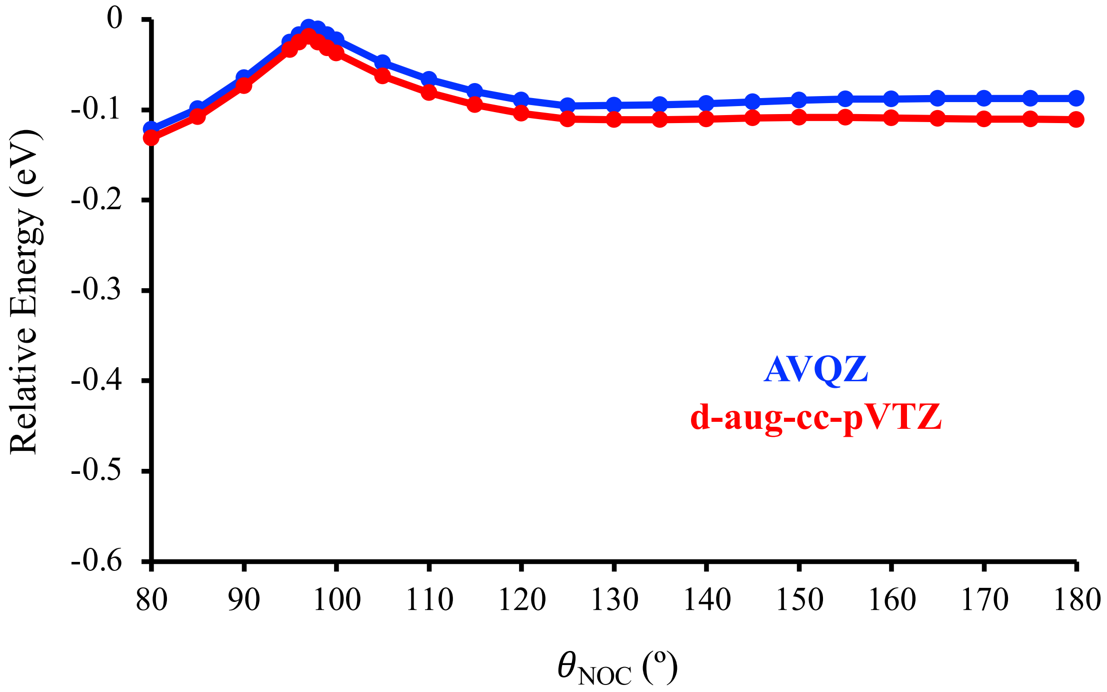

Figure S28: Energies for the D_2 states of ON-OCO as a function of the intermolecular angle, θ_{NOC} at a fixed intermolecular distance of R_{NO}=2.8 Å. The single-point energies were evaluated using a d-aug-cc-pVTZ basis set (red data points) or an AVQZ basis set (blue data points).

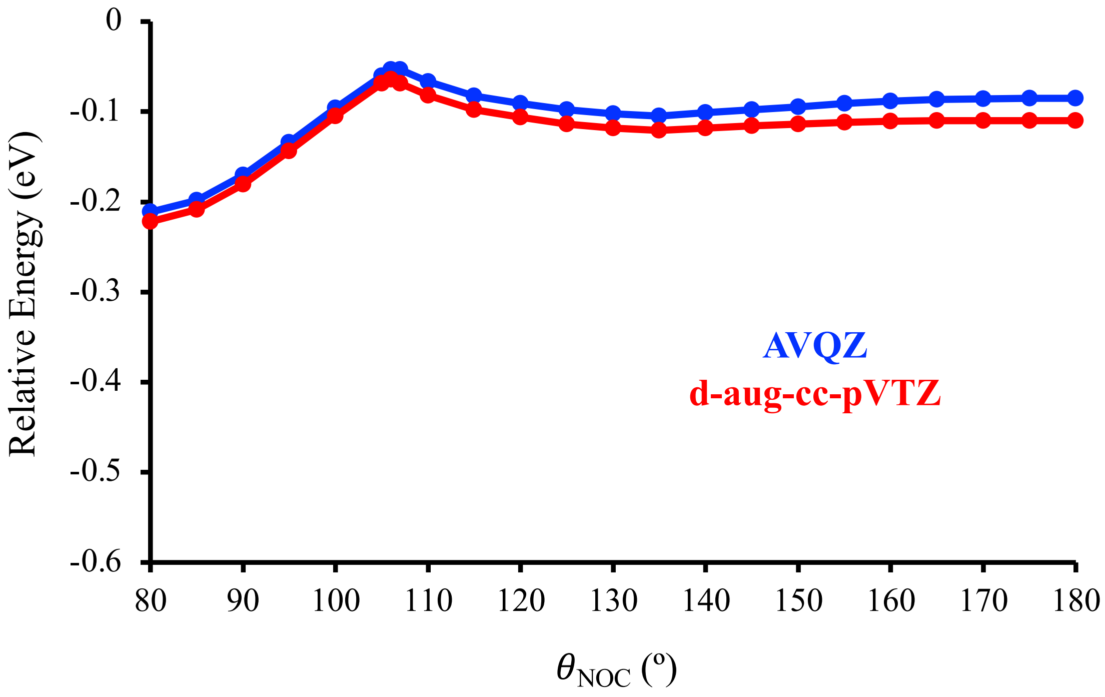

Figure S29: Energies for the D_2 states of ON-OCO as a function of the intermolecular angle, θ_{NOC} at a fixed intermolecular distance of R_{NO}=2.7 Å. The single-point energies were evaluated using a d-aug-cc-pVTZ basis set (red data points) or an AVQZ basis set (blue data points).

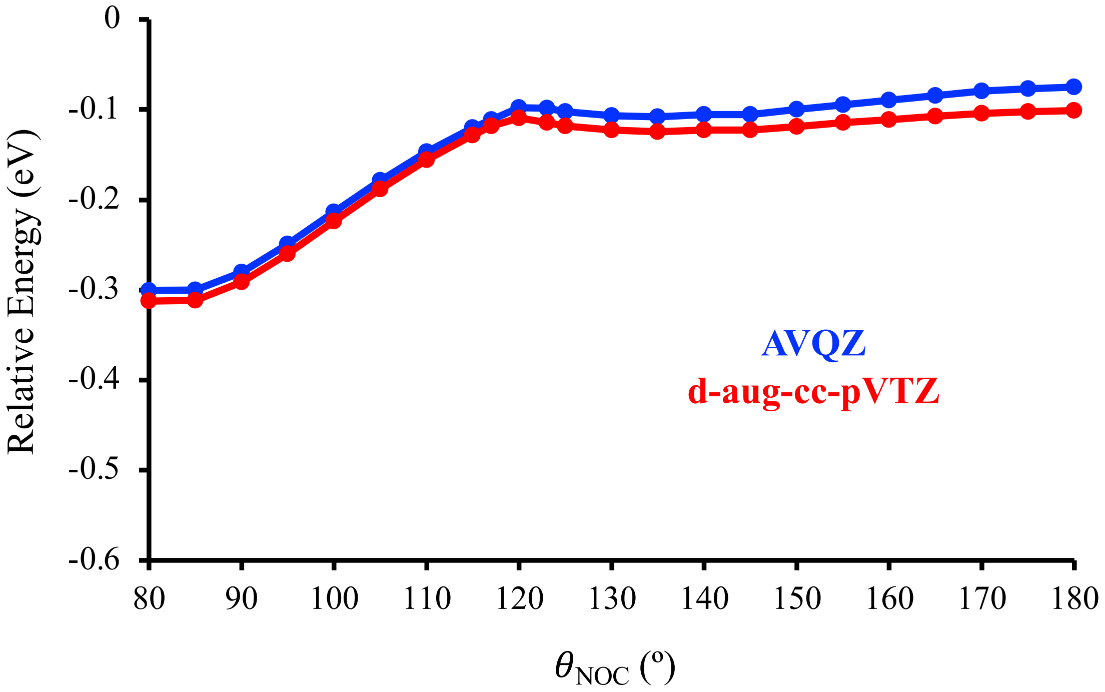

Figure S30: Energies for the D_2 states of ON-OCO as a function of the intermolecular angle, θ_{NOC} at a fixed intermolecular distance of R_{NO}=2.6 Å. The single-point energies were evaluated using a d-aug-cc-pVTZ basis set (red data points) or an AVQZ basis set (blue data points).

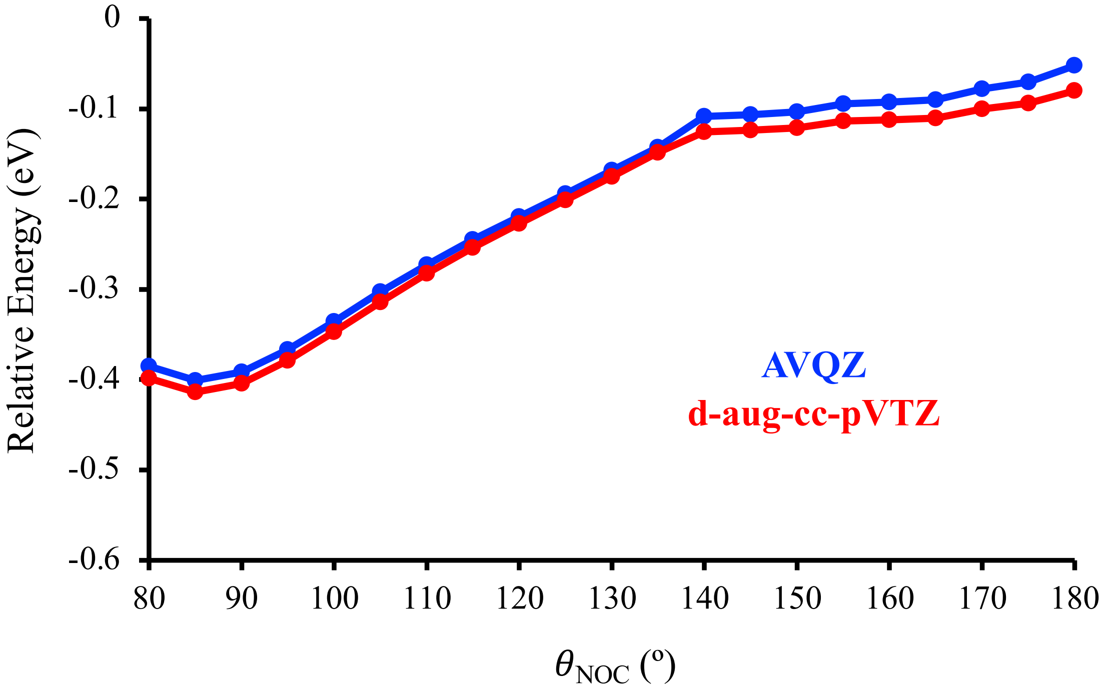

Figure S31: Energies for the D_2 states of ON-OCO as a function of the intermolecular angle, θ_{NOC} at a fixed intermolecular distance of R_{NO}=2.5 Å. The single-point energies were evaluated using a d-aug-cc-pVTZ basis set (red data points) or an AVQZ basis set (blue data points).

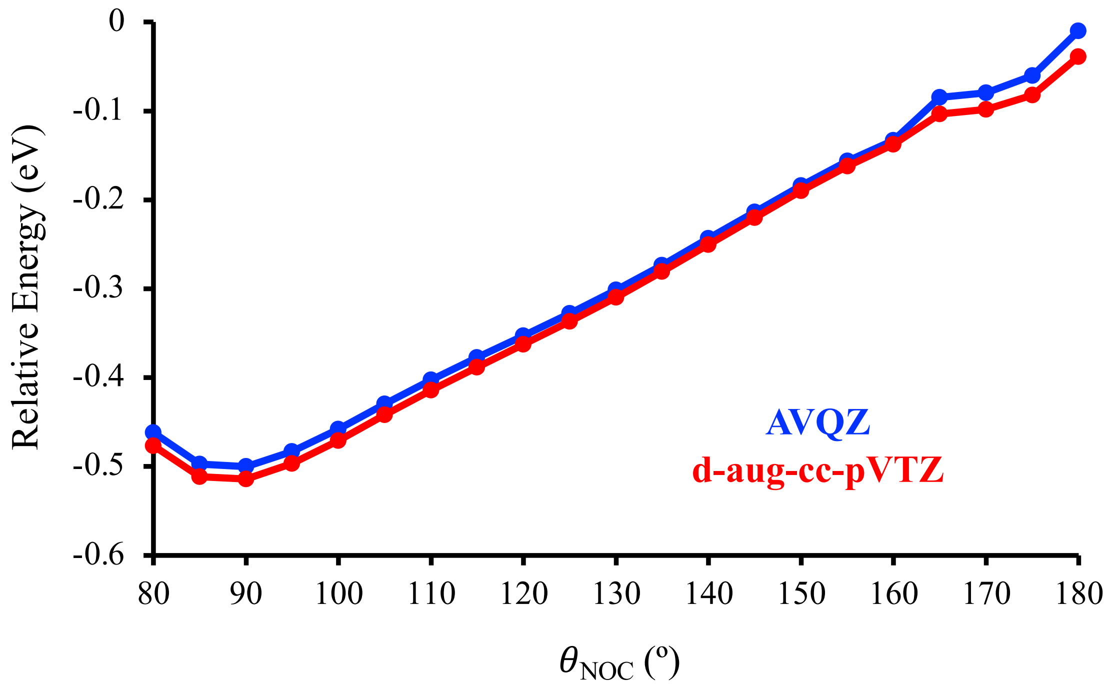

Figure S32: Energies for the D_2 states of ON-OCO as a function of the intermolecular angle, θ_{NOC} at a fixed intermolecular distance of R_{NO}=2.4 Å. The single-point energies were evaluated using a d-aug-cc-pVTZ basis set (red data points) or an AVQZ basis set (blue data points).

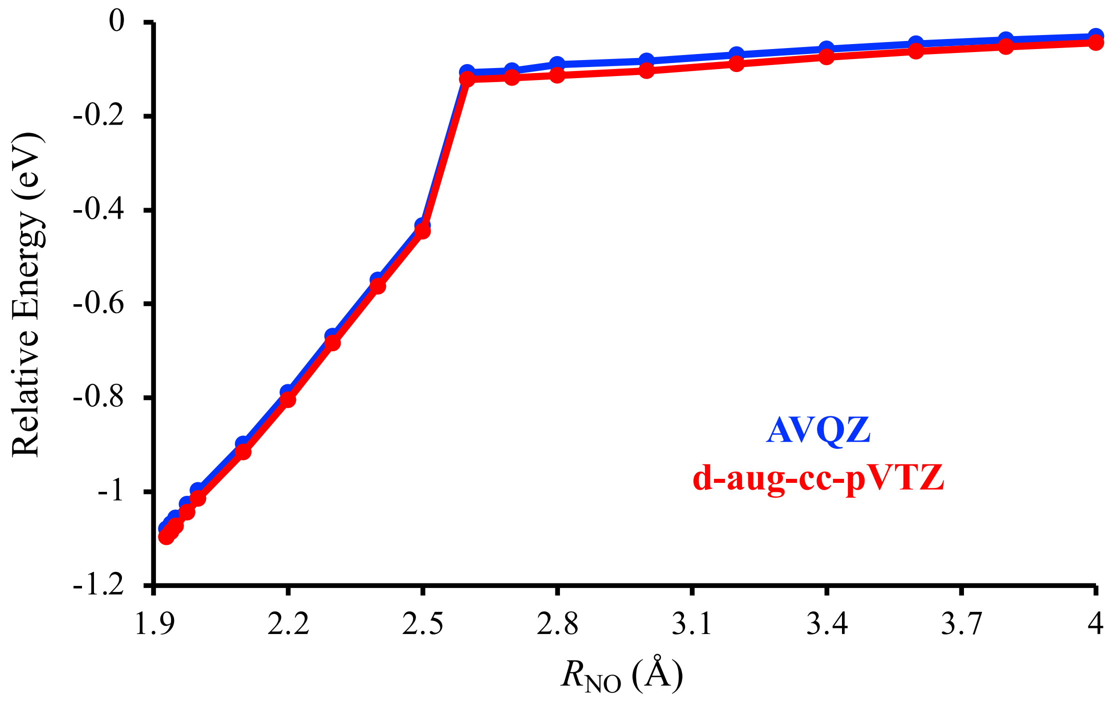

Figure S33: Energies for the D_2 states of ON-OCO as a function of the intermolecular distance R_{NO}. The single-point energies were evaluated using a d-aug-cc-pVTZ basis set (red data points) or an AVQZ basis set (blue data points).

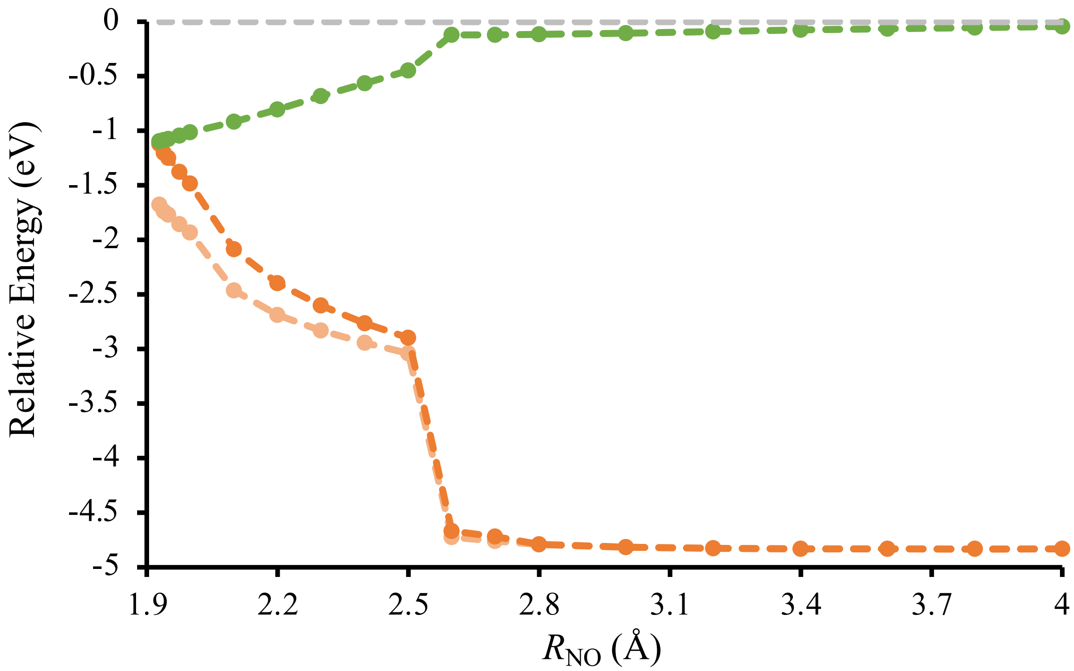

Figure S34: Energies for the D_0, D_1, and D_2 states of ON-OCO as a function of the intermolecular distance R_{NO}. The single-point energies were evaluated using a d-aug-cc-pVTZ basis set. Note that this plot shows an identical photochemical pathway to that shown in Figure 10, demonstrating that using a smaller d-aug-cc-pVTZ basis set to evaluate the electronic energies does not significantly impact the overall mechanistic picture developed by our calculations.

Table S1. Numerical values for the relative electronic energies of the D_2 state of ON+H_3CH and NO+H_3CH at the geometries shown in Figures S2 and S3. The energies are evaluated using a three-point extrapolation to the complete basis set limit and are reported relative to a D_2-optimized geometry with an intermolecular distance of 20 Å.

θ_{ONC} (°)	D_2 (cm-1)	θ_{NOC} (°)	D_2 (cm-1)
**90.00**	267.71	**90.00**	371.09
**100.00**	151.19	**100.00**	345.72
**110.00**	36.94	**110.00**	277.83
**120.00**	-65.28	**120.00**	213.32
**130.00**	-149.78	**130.00**	137.20
**140.00**	-211.14	**140.00**	68.46
**150.00**	-249.30	**150.00**	22.61
**160.00**	-270.94	**160.00**	-56.90
**170.00**	-280.24	**170.00**	-74.95
**180.00**	-283.55	**180.00**	-80.23

Table S2. Numerical values for the relative electronic energies of the D_2 state of ON+H_3CH and NO+H_3CH at the geometries shown in Figure 2. The energies are evaluated using a three-point extrapolation to the complete basis set limit and are reported relative to a D_2-optimized geometry with an intermolecular distance of 20 Å.

R_{NC} (Å)	D_2 (cm-1)	R_{OC} (Å)	D_2 (cm-1)
**2.75**	46.44	**2.85**	91.90
**2.88**	-152.51	**2.90**	42.22
**3.00**	-248.67	**2.95**	4.03
**3.05**	-267.31	**3.00**	-23.31
**3.10**	-278.83	**3.05**	-44.21
**3.15**	-283.55	**3.10**	-58.60
**3.20**	-285.49	**3.15**	-68.50
**3.25**	-284.38	**3.20**	-75.07
**3.38**	-267.24	**3.25**	-78.58
**3.50**	-245.30	**3.27**	-80.23
**3.75**	-195.93	**3.30**	-81.44
**4.00**	-155.44	**3.35**	-82.49
**4.25**	-125.45	**3.40**	-82.77
**4.50**	-104.25	**3.45**	-81.66
**4.75**	-88.34	**3.50**	-80.77
**5.00**	-74.15	**3.75**	-78.41
**5.50**	-51.38	**4.00**	-79.26
**6.00**	-36.26	**4.25**	-79.58
		**4.50**	-78.55
		**4.75**	-75.11
		**5.00**	-69.13
		**5.50**	-53.58
		**6.00**	-38.93

Table S3. Numerical values for the relative electronic energies of the first six electronic states of ON-H_3CH as a function of R_{NC}. The calculations were performed at the EOM-EA-CCSD/AVQZ//EOM-EA-CCSD/aug-cc-pVTZ level of theory and all energies are reported relative to a D_2-optimized geometry with an intermolecular distance of 20 Å.

R_{NC} (Å)	D_0 (eV)	D_1 (eV)	D_2 (eV)	D_3 (eV)	D_4 (eV)	D_5 (eV)
**2.00**	-3.3738	-3.0757	0.8693	1.8009	1.8535	1.9352
**2.25**	-4.1923	-4.0057	0.3238	1.2671	1.3094	1.4476
**2.50**	-4.5858	-4.4958	0.0897	1.0584	1.0861	1.3052
**2.75**	-4.7622	-4.7622	0.0078	1.0200	1.0201	1.3244
**2.88**	-4.8174	-4.8173	-0.0174	0.9872	0.9873	1.2977
**3.00**	-4.8535	-4.8534	-0.0295	0.9702	0.9703	1.2827
**3.05**	-4.8635	-4.8634	-0.0320	0.9663	0.9664	1.2784
**3.10**	-4.8717	-4.8716	-0.0335	0.9637	0.9638	1.2749
**3.15**	-4.8783	-4.8782	-0.0342	0.9622	0.9623	1.2718
**3.20**	-4.8838	-4.8837	-0.0344	0.9614	0.9615	1.2690
**3.25**	-4.8884	-4.8883	-0.0342	0.9613	0.9614	1.2665
**3.38**	-4.8964	-4.8964	-0.0321	0.9631	0.9632	1.2602
**3.50**	-4.9004	-4.9003	-0.0293	0.9667	0.9668	1.2534
**3.75**	-4.9038	-4.9037	-0.0232	0.9758	0.9758	1.2373
**4.00**	-4.9043	-4.9042	-0.0183	0.9845	0.9845	1.2186
**4.25**	-4.9043	-4.9043	-0.0148	0.9918	0.9919	1.1995
**4.50**	-4.9028	-4.9027	-0.0122	0.9978	0.9979	1.1817
**4.75**	-4.9017	-4.9017	-0.0103	1.0026	1.0026	1.1664
**5.00**	-4.9003	-4.9003	-0.0088	1.0063	1.0063	1.1540
**5.50**	-4.8999	-4.8999	-0.0065	1.0116	1.0116	1.1370
**6.00**	-4.8971	-4.8971	-0.0047	1.0148	1.0148	1.1285

Table S4. Numerical values for the relative electronic energies of the first six electronic states of NO-H_3CH as a function of R_{OC}. The calculations were performed at the EOM-EA-CCSD/AVQZ//EOM-EA-CCSD/aug-cc-pVTZ level of theory and all energies are reported relative to a D_2-optimized geometry with an intermolecular distance of 20 Å.

R_{NH} (Å)	D_0 (eV)	D_1 (eV)	D_2 (eV)	D_3 (eV)	D_4 (eV)	D_5 (eV)
**1.25**	-3.3799	-3.2995	1.1656	2.1452	2.1760	2.3604
**1.50**	-4.2492	-4.2064	0.4369	1.4118	1.4321	1.6600
**1.75**	-4.6357	-4.6357	0.1386	1.1214	1.1214	1.3822
**2.00**	-4.8040	-4.8040	0.0282	0.9954	0.9954	1.2655
**2.25**	-4.8373	-4.8373	0.0096	0.9747	0.9748	1.2447
**2.50**	-4.8497	-4.8497	0.0034	0.9680	0.9680	1.2371
**2.75**	-4.8597	-4.8597	-0.0014	0.9631	0.9631	1.2311
**3.00**	-4.8677	-4.8677	-0.0050	0.9596	0.9597	1.2260
**3.05**	-4.8744	-4.8744	-0.0076	0.9576	0.9576	1.2219
**3.10**	-4.8797	-4.8796	-0.0094	0.9565	0.9565	1.2184
**3.20**	-4.8839	-4.8838	-0.0107	0.9562	0.9562	1.2154
**3.25**	-4.8871	-4.8871	-0.0115	0.9565	0.9565	1.2127
**3.30**	-4.8895	-4.8895	-0.0120	0.9573	0.9573	1.2103
**3.35**	-4.8906	-4.8906	-0.0122	0.9577	0.9577	1.2093
**3.40**	-4.8918	-4.8918	-0.0123	0.9584	0.9585	1.2079
**3.45**	-4.8933	-4.8933	-0.0124	0.9599	0.9599	1.2057
**3.47**	-4.8945	-4.8945	-0.0124	0.9615	0.9616	1.2035
**3.50**	-4.8951	-4.8951	-0.0122	0.9634	0.9634	1.2013
**3.52**	-4.8958	-4.8958	-0.0120	0.9654	0.9654	1.1992
**3.55**	-4.8970	-4.8970	-0.0112	0.9751	0.9751	1.1876
**3.60**	-4.8956	-4.8956	-0.0107	0.9837	0.9837	1.1755
**3.65**	-4.8943	-4.8943	-0.0103	0.9911	0.9911	1.1639
**3.70**	-4.8932	-4.8932	-0.0099	0.9971	0.9971	1.1537
**3.75**	-4.8925	-4.8925	-0.0093	1.0018	1.0018	1.1453
**3.80**	-4.8920	-4.8920	-0.0086	1.0056	1.0056	1.1384
**3.85**	-4.8914	-4.8914	-0.0068	1.0110	1.0110	1.1294
**3.90**	-4.8913	-4.8913	-0.0051	1.0145	1.0145	1.1251
**3.95**	-3.3799	-3.2995	1.1656	2.1452	2.1760	2.3604
**4.00**	-4.2492	-4.2064	0.4369	1.4118	1.4321	1.6600
**4.25**	-4.6357	-4.6357	0.1386	1.1214	1.1214	1.3822
**4.50**	-4.8040	-4.8040	0.0282	0.9954	0.9954	1.2655
**4.75**	-4.8373	-4.8373	0.0096	0.9747	0.9748	1.2447
**5.00**	-4.8497	-4.8497	0.0034	0.9680	0.9680	1.2371
**5.50**	-4.8597	-4.8597	-0.0014	0.9631	0.9631	1.2311
**6.00**	-4.8677	-4.8677	-0.0050	0.9596	0.9597	1.2260

Table S5. Numerical values for the energy of the D_2 state as a function of the intermolecular distance when the NO is interacting with the CH_3 group of CH_3OH. This is the data used in the plots shown in Figure 4a. The geometry optimizations were performed using EOM-EA-CCSD with a d-aug-cc-pVTZ basis set for the C, N, and O atoms and an aug-cc-pVDZ basis set for the H atoms. The single-point energies were evaluated at the EOM-EA-CCSD/d-aug-cc-pVTZ level of theory and all energies are reported relative to a D_2-optimized geometry with an intermolecular distance of 20 Å.

R_{NC} (Å)	ON-CH_3OH D_2 (eV)	R_{OC} (Å)	NO-CH_3OH D_2 (eV)
**1.8**	1.5483	**2**	0.9962
**2**	0.7260	**2.2**	0.4474
**2.2**	0.3099	**2.4**	0.1884
**2.4**	0.1123	**2.6**	0.0747
**2.6**	0.0250	**2.8**	0.0279
**2.8**	-0.0081	**3**	0.0088
**2.9**	-0.0147	**3.2**	0.0004
**3**	-0.0175	**3.4**	-0.0044
**3.1**	-0.0181	**3.6**	-0.0082
**3.2**	-0.0175	**3.7**	-0.0100
**3.4**	-0.0154	**3.8**	-0.0114
**3.6**	-0.0135	**3.9**	-0.0127
**3.8**	-0.0123	**4**	-0.0138
**4**	-0.0117	**4.1**	-0.0148
**4.2**	-0.0114	**4.2**	-0.0156
**4.4**	-0.0110	**4.4**	-0.0166
**4.6**	-0.0104	**4.5**	-0.0168
**4.8**	-0.0097	**4.6**	-0.0170
**5**	-0.0090	**4.7**	-0.0170
**5.5**	-0.0073	**4.8**	-0.0169
**6**	-0.0056	**4.9**	-0.0166
**6.5**	-0.0040	**5**	-0.0162
**7**	-0.0028	**5.2**	-0.0153
		**5.4**	-0.0142
		**5.6**	-0.0130
		**5.8**	-0.0118
		**6**	-0.0106
		**6.2**	-0.0095
		**6.4**	-0.0085
		**6.6**	-0.0076
		**6.8**	-0.0068
		**7**	-0.0061

Table S6. Numerical values of the energy of the D_2 state as a function of the N-C-O intermolecular angle (θ_{NCO}) at fixed intermolecular distances R_{NC}=3.0 Å, R_{NC}=3.2, R_{NC}=3.4 Å, and R_{NC}=3.6 Å. This is the data plotted in Figure 4b. The geometry optimizations were performed using EOM-EA-CCSD with a d-aug-cc-pVTZ basis set for the C, N, and O atoms and an aug-cc-pVDZ basis set for the H atoms. The single-point energies were evaluated at the EOM-EA-CCSD/d-aug-cc-pVTZ level of theory and all energies are reported relative to a D_2-optimized geometry with an intermolecular distance of 20 Å.

θ_{NCO}	R_{NC} = 3.0 Å	R_{NC} = 3.2 Å	R_{NC} = 3.4 A	R_{NC} = 3.6 A
**180**	-0.01752	-0.01758	-0.01545	-0.01355
**170**	-0.01201	-0.01464	-0.01382	-0.01261
**160**	-0.000915	-0.00823	-0.01075	-0.01099
**150**	0.01511	-0.00108	-0.00758	-0.00974
**140**	0.02725	0.00383	-0.00622	-0.00988
**130**	0.02629	0.00198	-0.00909	-0.01289
**120**	0.01335	-0.00753	-0.01605	-0.01873
**110**	-0.00774	-0.02311	-0.02815	-0.02838
**100**	-0.03827	-0.04616	-0.04591	-0.04248
**90**	-0.07923	-0.07834	-0.07120	-0.06282

Table S7. Numerical values of the energy of the D_2 state of ON-H_3COH as a function of R_{NC}, the distance between the nitrogen of NO and the carbon of methanol. This is the data plotted in Figure S6. The geometry optimizations were performed at the EOM-EA-CCSD/aug-cc-pVDZ level of theory or used EOM-EA-CCSD with a d-aug-cc-pVTZ basis set for C, N, and O atoms and an aug-cc-pVDZ basis set for the H atoms. For all geometries, the single-point energies were evaluated at the EOM-EA-CCSD/d-aug-cc-pVTZ level of theory and all energies are reported relative to a D_2-optimized geometry with an intermolecular distance of 20 Å.

R_{NC} (Å)	aug-cc-pvdz Basis Set	d-aug-cc-pvtz/aug-cc-pVDZ Basis Set
**1.8**	1.55019	1.54833
**2**	0.72986	0.72604
**2.2**	0.31537	0.30992
**2.4**	0.11849	0.11238
**2.6**	0.03236	0.02501
**2.8**	-0.00370	-0.00812
**2.9**	-0.01019	-0.01476
**3**	-0.01367	-0.01752
**3.1**	-0.01358	-0.01810
**3.2**	-0.01362	-0.01758
**3.4**	-0.01169	-0.01545
**3.6**	-0.00976	-0.01355
**3.8**	-0.00870	-0.01236
**4**	-0.00823	-0.01178
**4.2**	-0.00786	-0.01145
**4.4**	-0.00790	-0.01105
**4.6**	-0.00754	-0.01044
**4.8**	-0.00709	-0.00974
**5**	-0.00687	-0.00904

Table S8. Numerical values of the energy of the D_2 state as a function of the O-C-O intermolecular angle (θ_{OCO}) at fixed intermolecular distances R_{OC}=5.0 Å and R_{OC}=4.6 Å. This data is plotted in Figure S7. The geometry optimizations were performed using EOM-EA-CCSD with a d-aug-cc-pVTZ basis set for the C, N, and O atoms and an aug-cc-pVDZ basis set for the H atoms. The single-point energies were evaluated at the EOM-EA-CCSD/d-aug-cc-pVTZ level of theory and all energies are reported relative to a D_2-optimized geometry with an intermolecular distance of 20 Å.

θ_{OCO}	R_{OC}=4.6 Å	R_{OC}=5.0 Å
**180**	-0.01708	-0.01627
**170**	-0.01661	-0.01595
**160**	-0.01600	-0.01561
**150**	-0.01524	-0.01521
**140**	-0.01432	-0.01447
**130**	-0.01372	-0.01381
**120**	-0.01438	-0.01402
**110**	-0.01632	-0.01516
**100**	-0.01948	-0.01711
**90**	-0.02480	-0.02007

Table S9. Numerical values of the energy of the D_0, D_1, D_2, D_3, D_4, and D_5 states as a function of the intermolecular distance, R_{NO}, when the N of NO is interacting with O of CH_3OH. The geometry optimizations were performed using EOM-EA-CCSD with a d-aug-cc-pVTZ basis set for the C, N, and O atoms and an aug-cc-pVDZ basis set for the H atoms. The single-point energies were evaluated at the EOM-EA-CCSD/d-aug-cc-pVTZ level of theory and all energies are reported relative to a D_2-optimized geometry with an intermolecular distance of 20 Å. This data is plotted in Figures 5a and S8.

R_{NO} (Å)	D_0 (eV)	D_1 (eV)	D_2 (eV)	D_3 (eV)	D_4 (eV)	D_5 (eV)
**10**	-4.8361	-4.8359	-0.0013	1.0175	1.0208	1.1303
**9.5**	-4.8359	-4.8357	-0.0017	1.0165	1.0204	1.1313
**9**	-4.8357	-4.8355	-0.0023	1.0151	1.0198	1.1324
**8.5**	-4.8355	-4.8352	-0.0031	1.0133	1.0189	1.1338
**8**	-4.8353	-4.8348	-0.0042	1.0107	1.0176	1.1354
**7.5**	-4.8350	-4.8344	-0.0058	1.0073	1.0157	1.1373
**7**	-4.8346	-4.8339	-0.0081	1.0024	1.0127	1.1397
**6.5**	-4.8341	-4.8332	-0.0114	0.9955	1.0083	1.1426
**6**	-4.8336	-4.8323	-0.0165	0.9857	1.0013	1.1461
**5.75**	-4.8331	-4.8315	-0.0197	0.9795	0.9967	1.1484
**5.5**	-4.8327	-4.8308	-0.0239	0.9718	0.9907	1.1506
**5.25**	-4.8322	-4.8299	-0.0291	0.9624	0.9832	1.1530
**5**	-4.8316	-4.8288	-0.0355	0.9510	0.9738	1.1553
**4.75**	-4.8310	-4.8275	-0.0437	0.9368	0.9620	1.1570
**4.55**	-4.8255	-4.8241	-0.0547	0.9241	0.9432	1.1616
**4.35**	-4.8233	-4.8201	-0.0644	0.9073	0.9284	1.1485
**4.15**	-4.8229	-4.8209	-0.0799	0.8906	0.9080	1.1468
**3.95**	-4.8214	-4.8169	-0.0950	0.8632	0.8865	1.1306
**3.75**	-4.8194	-4.8127	-0.1148	0.8323	0.8606	1.1096
**3.55**	-4.8173	-4.8047	-0.1392	0.7920	0.8270	1.0733
**3.35**	-4.8384	-4.7994	-0.1723	0.7209	0.7482	0.9638
**2.75**	-4.8361	-4.7842	-0.2161	0.6512	0.6894	0.9055
**2.55**	-4.8276	-4.7568	-0.2686	0.5673	0.6218	0.8332
**2.5**	-4.8181	-4.7345	-0.2978	0.5203	0.5851	0.7922
**2.45**	-4.8044	-4.7046	-0.3282	0.4709	0.5476	0.7496
**2.4**	-4.7818	-4.6612	-0.3593	0.4223	0.5131	0.7049
**2.35**	-4.7655	-4.6139	-0.4088	0.3144	0.5179	0.5641
**2.3**	-4.7442	-4.5724	-0.4253	0.2973	0.5023	0.5609
**2.25**	-4.7205	-4.5256	-0.4423	0.2802	0.4870	0.5558
**2.2**	-4.6924	-4.4714	-0.4595	0.2629	0.4721	0.5487
**2.15**	-4.6597	-4.4091	-0.4766	0.2469	0.4591	0.5412
**2.1**	-4.6223	-4.3372	-0.4934	0.2332	0.4485	0.5344

**2.05**	-4.5771	-4.2524	-0.5098	0.2239	0.4424	0.5304
**2**	-4.5259	-4.1553	-0.5258	0.2213	0.4407	0.5332
**1.95**	-4.4655	-4.0406	-0.5412	0.2223	0.4423	0.5359
**1.9**	-4.3955	-3.9067	-0.5559	0.2305	0.4499	0.5441
**1.85**	-4.3131	-3.7490	-0.5697	0.2461	0.4641	0.5588
**1.84**	-4.2139	-3.5620	-0.5824	0.2757	0.4875	0.5825
**1.83**	-4.0952	-3.3388	-0.5936	0.3218	0.5257	0.6221
**1.82**	-3.9533	-3.0733	-0.6030	0.3863	0.5816	0.6793
**1.81**	-3.7771	-2.7495	-0.6099	0.4789	0.6681	0.7660
**1.8**	-3.7360	-2.6757	-0.6109	0.5025	0.6911	0.7888
**1.79**	-3.6924	-2.5980	-0.6117	0.5285	0.7168	0.8142
**1.78**	-3.6453	-2.5155	-0.6124	0.5576	0.7462	0.8432

Table S10. Numerical values of the energy of the D_1 and D_2 states as a function of the O-H bond length (r_{OH}) at a fixed intermolecular distance of R_{NO}=1.78 Å. This data is plotted in Figure 5b. The geometry optimizations were performed using EOM-EA-CCSD with a d-aug-cc-pVTZ basis set for the C, N, and O atoms and an aug-cc-pVDZ basis set for the H atoms. The single-point energies were evaluated at the EOM-EA-CCSD/d-aug-cc-pVTZ level of theory and all energies are reported relative to a D_2-optimized geometry with an intermolecular distance of 20 Å.

r_{OH} (Å)	D_1 (eV)	D_2 (eV)
**1.1**	-2.0730	-0.6128
**1.14**	-1.8791	-0.6067
**1.18**	-1.6400	-0.6238
**1.22**	-1.4052	-0.6322
**1.26**	-1.1601	-0.6465
**1.3**	-0.9103	-0.6652
**1.32**	-0.7755	-0.6756
**1.325**	-0.7409	-0.6780
**1.33**	-0.7008	-0.6809

Table S11. Numerical values of the energy of the D_1 and D_2 states as a function of the O-H bond length (r_{OH}) at a fixed intermolecular distance of R_{NO}=1.78 Å. The geometry optimizations were performed using EOM-EA-CCSD with a d-aug-cc-pVTZ basis set for the C, N, and O atoms and an aug-cc-pVDZ basis set for the H atoms. The single-point energies were evaluated at the EOM-EA-CCSD/d-aug-cc-pVTZ level of theory and all energies are reported relative to a D_2-optimized geometry with an intermolecular distance of 20 Å. The only constraints included in these calculations were the R_{NO} and r_{OH} bond lengths; the calculations were initialized from geometries with the NO oriented above the methyl group. This data is plotted in Figure S13.

r_{OH} (Å)	D_1 (eV)	D_2 (eV)
**1.08**	-2.1147	-0.6125
**1.12**	-1.9221	-0.6144
**1.16**	-1.7002	-0.6177
**1.2**	-1.4689	-0.6263
**1.24**	-1.2254	-0.6409
**1.28**	-0.9783	-0.6595
**1.32**	-0.7400	-0.6749
**1.3225**	-0.7247	-0.6753
**1.325**	-0.7040	-0.6759

Table S12. Numerical values of the energy of the D_1 and D_2 states as a function of the O-H bond length (r_{OH}) at a fixed intermolecular distance of R_{NO}=1.78 Å, R_{NO}=1.84 Å, and R_{NO}=1.90 Å. This data is plotted in Figure S14. The geometry optimizations were performed using EOM-EA-CCSD with a d-aug-cc-pVTZ basis set for the C, N, and O atoms and an aug-cc-pVDZ basis set for the H atoms. The single-point energies were evaluated at the EOM-EA-CCSD/d-aug-cc-pVTZ level of theory and all energies are reported relative to a D_2-optimized geometry with an intermolecular distance of 20 Å.

r_{OH} (Å)	R_{NO} = 1.78 Å D_1 (eV)	R_{NO} = 1.78 Å D_2 (eV)	R_{NO} = 1.84 Å D_1 (eV)	R_{NO} = 1.84 Å D_2 (eV)	R_{NO} = 1.90 Å D_1 (eV)	R_{NO} = 1.90 Å D_2 (eV)
**1.1**	-2.0730	-0.6128	-2.4763	-0.5913	-2.8152	-0.5586
**1.14**	-1.8791	-0.6067	-2.2854	-0.5705	-2.6256	-0.5231
**1.18**	-1.6400	-0.6238	-2.0448	-0.5685	-2.3829	-0.5019
**1.22**	-1.4052	-0.6322	-1.8122	-0.5635	-2.1522	-0.4834
**1.26**	-1.1601	-0.6465	-1.5707	-0.5657	-1.9125	-0.4732
**1.3**	-0.9103	-0.6652	-1.3268	-0.5738	-1.6704	-0.4703
**1.32**	-0.7755	-0.6756	-1.2041	-0.5781	-1.5638	-0.4658
**1.33**	-0.7008	-0.6809	-1.1422	-0.5803	-1.5027	-0.4644

Table S13. Numerical values of the energy of the D_1 and D_2 states as a function of the O-C bond length (r_{OC}) at a fixed intermolecular distance of R_{NO}=1.78 Å. This data is plotted in Figure S15. The geometry optimizations were performed using EOM-EA-CCSD with a d-aug-cc-pVTZ basis set for the C, N, and O atoms and an aug-cc-pVDZ basis set for the H atoms. The single-point energies were evaluated at the EOM-EA-CCSD/d-aug-cc-pVTZ level of theory and all energies are reported relative to a D_2-optimized geometry with an intermolecular distance of 20 Å.

r_{OC} (Å)	D_1 (eV)	D_2 (eV)
**1.44**	-2.0696	-0.6124
**1.48**	-2.1296	-0.6033
**1.52**	-2.0972	-0.5632
**1.56**	-2.0224	-0.5015
**1.6**	-1.9205	-0.4282
**1.64**	-1.8010	-0.3555
**1.68**	-1.6619	-0.3020

Table S14. Energies for the D_2 states of two different molecular orientations, NO-CO_2 and ON-CO_2, as a function of the intermolecular bond angles θ_{ONC} or θ_{NOC} at representative intermolecular distances. The calculations were performed at the EOM-EA-CCSD/AVQZ//EOM-EA-CCSD/aug-cc-pVTZ level of theory and all energies are reported relative to a D_2-optimized geometry with an intermolecular distance of 20 Å. This data is plotted in Figure S16.

θ_{ONC} or θ_{NOC} (°)	ON-CO_2 R_{NC} =4.0 Å D_2 (cm^{-1})	ON-CO_2 R_{NC} =3.5 Å D_2 (cm^{-1})	ON-CO_2 R_{NC} =3.0 Å D_2 (cm^{-1})	NO-CO_2 R_{OC} =4.0 Å D_2 (cm^{-1})	NO-CO_2 R_{OC} =3.5 Å D_2 (cm^{-1})	NO-CO_2 R_{OC} =3.0 Å D_2 (cm^{-1})
**180**	-9.11	-11.78	-5.14	-6.20	4.37	6.57
**165**	-9.05	-11.85	-4.77	-6.05	5.30	7.13
**150**	-8.96	-11.94	-3.70	-5.55	7.97	8.71
**135**	-8.93	-12.13	-2.22	-4.96	10.14	10.84
**120**	-9.07	-12.39	-0.66	-4.35	15.81	12.83
**105**	-9.36	-12.99	0.64	-3.94	20.27	13.81
**90**	-9.68	-13.08	1.72	-3.97	22.50	13.64

Table S15. Energies of the D_2 states of ON-OCO as a function of θ_{NOC} for the intermolecular distances R_{NO}=3.5, 3.3, 3.1, and 2.9 Å. This data was calculated at the EOM-EA-CCSD/AVQZ //EOM-EA-CCSD/aug-cc-pVDZ level of theory. All energies are reported relative to a D_2-optimized geometry with an intermolecular distance of 20 Å. This data is plotted in Figure 6 in the paper.

θ_{NOC} (°)	R_{NO}=3.5 Å D_2 (eV)	R_{NO}=3.3 Å D_2 (eV)	R_{NO}=3.1 Å D_2 (eV)	R_{NO}=2.9 Å D_2 (eV)
**180**	-0.0490	-0.0608	-0.0738	-0.0848
**175**	-0.0491	-0.0608	-0.0739	-0.0850
**170**	-0.0491	-0.0609	-0.0739	-0.0849
**165**	-0.0490	-0.0607	-0.0737	-0.0848
**160**	-0.0488	-0.0605	-0.0734	-0.0847
**155**	-0.0486	-0.0602	-0.0731	-0.0846
**150**	-0.0482	-0.0598	-0.0727	-0.0847
**145**	-0.0476	-0.0592	-0.0722	-0.0848
**140**	-0.0469	-0.0585	-0.0715	-0.0853
**135**	-0.0459	-0.0573	-0.0706	-0.0860
**130**	-0.0448	-0.0563	-0.0697	-0.0862
**125**	-0.0429	-0.0545	-0.0685	-0.0858
**120**	-0.0408	-0.0521	-0.0671	-0.0851
**115**	-0.0402	-0.0492	-0.0668	-0.0773
**110**	-0.0422	-0.0501	-0.0589	-0.0658
**105**	-0.0362	-0.0419	-0.0475	-0.0500
**100**	-0.0279	-0.0306	-0.0321	-0.0289
**95**	-0.0089	-0.0093	-0.0068	-0.0020
**90**	0.0024	0.0055	0.0127	0.0307
**85**	0.0132	0.0187	0.0280	0.0464
**80**	0.0228	0.0302	0.0423	0.0639

Table S16. Energies for the D_2 states of two different molecular orientations, NO-OCO and ON-OCO, based on the θ_{OOC} or θ_{NOC}, respectively. The intermolecular distance R_{NO} or R_{OO} is 3.3 Å. Throughout the various angle changes, the NO-OCO confirmation is higher in energy. The maximum difference in energy is 0.02 eV. The calculations were performed at the EOM-EA-CCSD/AVDZ//EOM-EA-CCSD/aug-cc-pVTZ level of theory and the data is plotted in Figure S17.

θ_{NOC}	ON-OCO (eV)	θ_{OOC}	NO-OCO (eV)
**180**	-0.0608	**180**	-0.0391
**175**	-0.0609	**175**	-0.0392
**170**	-0.0607	**170**	-0.0396
**165**	-0.0605	**165**	-0.0398
**160**	-0.0602	**160**	-0.0401
**155**	-0.0598	**155**	-0.0406
**150**	-0.0592	**150**	-0.0409
**145**	-0.0585	**145**	-0.0414
**140**	-0.0573	**140**	-0.0417
**135**	-0.0563	**135**	-0.0418
**130**	-0.0545	**130**	-0.0416
**125**	-0.0521	**125**	-0.0389
**120**	-0.0492	**120**	-0.0363
**115**	-0.0501	**115**	-0.0326
**110**	-0.0419	**110**	-0.0266
**105**	-0.0306	**105**	-0.0184
**100**	-0.0093	**100**	-0.0075
**95**	0.0055	**95**	0.0066
**90**	0.0187	**90**	0.0233
**85**	0.0302	**85**	0.0416
**80**	-0.0608	**80**	-0.0391

Table S17. Energies for the D_2 states of two different molecular orientations, NO-OCO and ON-OCO, based on the θ_{OOC} or θ_{NOC}, respectively. The intermolecular distance R_{NO} or R_{OO} is 3.1 Å. Throughout the various angle changes, the NO-OCO confirmation is higher in energy. The maximum difference in energy is 0.02 eV. The calculations were performed at the EOM-EA-CCSD/AVDZ//EOM-EA-CCSD/aug-cc-pVTZ level of theory and the data is plotted in Figure S18.

θ_{NOC}	ON-OCO (eV)	θ_{OOC}	NO-OCO (eV)
**180**	-0.0738	**180**	-0.0501
**175**	-0.0739	**175**	-0.0504
**170**	-0.0737	**170**	-0.0503
**165**	-0.0734	**165**	-0.0505
**160**	-0.0731	**160**	-0.0507
**155**	-0.0727	**155**	-0.0509
**150**	-0.0722	**150**	-0.0512
**145**	-0.0715	**145**	-0.0520
**140**	-0.0706	**140**	-0.0519
**135**	-0.0697	**135**	-0.0518
**130**	-0.0685	**130**	-0.0512
**125**	-0.0671	**125**	-0.0504
**120**	-0.0668	**120**	-0.0482
**115**	-0.0589	**115**	-0.0445
**110**	-0.0475	**110**	-0.0387
**105**	-0.0321	**105**	-0.0307
**100**	-0.0068	**100**	-0.0191
**95**	0.0127	**95**	-0.0049
**90**	0.0280	**90**	0.0125
**85**	0.0423	**85**	0.0312
**80**	-0.0738	**80**	-0.0501

Table S18. Energies for the D_2 states of two different molecular orientations, NO-OCO and ON-OCO, based on the θ_{OOC} or θ_{NOC}, respectively. The intermolecular distance R_{NO} or R_{OO} is 2.9 Å. Throughout the various angle changes, the NO-OCO confirmation is higher in energy. The maximum difference in energy is 0.02 eV. The calculations were performed at the EOM-EA-CCSD/AVDZ//EOM-EA-CCSD/aug-cc-pVTZ level of theory and the data is plotted in Figure S19.

θ_{NOC}	ON-OCO (eV)	θ_{OOC}	NO-OCO (eV)
**180**	-0.0848	**180**	-0.0617
**175**	-0.0849	**175**	-0.0618
**170**	-0.0848	**170**	-0.0618
**165**	-0.0847	**165**	-0.0618
**160**	-0.0846	**160**	-0.0619
**155**	-0.0847	**155**	-0.0622
**150**	-0.0848	**150**	-0.0623
**145**	-0.0853	**145**	-0.0625
**140**	-0.0860	**140**	-0.0624
**135**	-0.0862	**135**	-0.0620
**130**	-0.0858	**130**	-0.0607
**125**	-0.0851	**125**	-0.0590
**120**	-0.0773	**120**	-0.0552
**115**	-0.0658	**115**	-0.0492
**110**	-0.0500	**110**	-0.0409
**105**	-0.0289	**105**	-0.0288
**100**	-0.0020	**100**	-0.0128
**95**	0.0307	**95**	0.0071
**90**	0.0464	**90**	0.0296
**85**	0.0639	**85**	0.0524
**80**	-0.0848	**80**	-0.0617

Table S19. Energies of the D_2 states of ON-OCO as a function of θ_{NOC} for the intermolecular distances R_{NO}=2.8, 2.7, and 2.6 Å. This data was calculated at the EOM-EA-CCSD/AVQZ//EOM-EA-CCSD/aug-cc-pVDZ level of theory. All energies are reported relative to a D_2-optimized geometry with an intermolecular distance of 20 Å. This data is plotted in Figure 7 in the paper.

θ_{NOC} (°)	R_{NO}=2.8 Å D_2 (eV)	θ_{NOC} (°)	R_{NO}=2.7 Å D_2 (eV)	θ_{NOC} (°)	R_{NO}=2.6 Å D_2 (eV)
**180**	-0.0875	**180**	-0.0852	**180**	-0.0750
**175**	-0.0872	**175**	-0.0851	**175**	-0.0769
**170**	-0.0874	**170**	-0.0856	**170**	-0.0796
**165**	-0.0875	**165**	-0.0867	**165**	-0.0844
**160**	-0.0878	**160**	-0.0885	**160**	-0.0896
**155**	-0.0884	**155**	-0.0911	**155**	-0.0947
**150**	-0.0894	**150**	-0.0944	**150**	-0.0999
**145**	-0.0910	**145**	-0.0976	**145**	-0.1055
**140**	-0.0930	**140**	-0.1009	**140**	-0.1053
**135**	-0.0945	**135**	-0.1048	**135**	-0.1081
**130**	-0.0952	**130**	-0.1024	**130**	-0.1066
**125**	-0.0956	**125**	-0.0979	**125**	-0.1022
**120**	-0.0895	**120**	-0.0908	**123**	-0.0986
**115**	-0.0801	**115**	-0.0828	**120**	-0.0981
**110**	-0.0666	**110**	-0.0665	**117**	-0.1110
**105**	-0.0479	**107**	-0.0534	**115**	-0.1205
**100**	-0.0227	**106**	-0.0535	**110**	-0.1469
**99**	-0.0172	**105**	-0.0602	**105**	-0.1785
**98**	-0.0106	**100**	-0.0960	**100**	-0.2136
**97**	-0.0090	**95**	-0.1344	**95**	-0.2494
**96**	-0.0170	**90**	-0.1707	**90**	-0.2805
**95**	-0.0250	**85**	-0.1985	**85**	-0.3001
**90**	-0.0646	**80**	-0.2113	**80**	-0.3005
**85**	-0.0987				
**80**	-0.1215				

Table S20. Energies of the D_2 states of ON-OCO as a function of θ_{NOC} for the intermolecular distances R_{NO}=2.5 and 2.4 Å. This data was calculated at the

EOM-EA-CCSD/AVQZ//EOM-EA-CCSD/aug-cc-pVDZ level of theory. All energies are reported relative to a D_2-optimized geometry with an intermolecular distance of 20 Å. This data is plotted in Figure 7 in the paper.

θ_{NOC} (°)	R_{NO}=2.5 Å D_2 (eV)	R_{NO}=2.4 Å D_2 (eV)
**180**	-0.0522	-0.0098
**175**	-0.0701	-0.0601
**170**	-0.0778	-0.0795
**165**	-0.0900	-0.0849
**160**	-0.0926	-0.1333
**155**	-0.0946	-0.1566
**150**	-0.1032	-0.1840
**145**	-0.1067	-0.2135
**140**	-0.1085	-0.2436
**135**	-0.1425	-0.2733
**130**	-0.1682	-0.3015
**125**	-0.1939	-0.3278
**120**	-0.2193	-0.3531
**115**	-0.2446	-0.3776
**110**	-0.2725	-0.4024
**105**	-0.3028	-0.4296
**100**	-0.3355	-0.4580
**95**	-0.3668	-0.4831
**90**	-0.3914	-0.5001
**85**	-0.4008	-0.4971
**80**	-0.3852	-0.4620

Table S21. Energies of the D_1 and D_2 states of ON-OCO as a function of θ_{NOC} for the intermolecular distances R_{NO}=2.8 and 2.6 Å. This data was calculated at the EOM-EA-CCSD/AVQZ//EOM-EA-CCSD/aug-cc-pVDZ level of theory. All energies are reported relative to a D_2-optimized geometry with an intermolecular distance of 20 Å. This data

is plotted in Figure S20 in the Supporting Information.

θ_{NOC} (°)	R_{NO}=2.8 Å D$_1$ (eV)	R_{NO}=2.8 Å D$_2$ (eV)	θ_{NOC} (°)	R_{NO}=2.6 Å D$_1$ (eV)	R_{NO}=2.6 Å D$_2$ (eV)
**180**	-5.0076	-0.0875	**180**	-4.9459	-0.0750
**175**	-5.0074	-0.0872	**175**	-4.9438	-0.0769
**170**	-5.0067	-0.0874	**170**	-4.9399	-0.0796
**165**	-5.0059	-0.0875	**165**	-4.9345	-0.0844
**160**	-5.0047	-0.0878	**160**	-4.9274	-0.0896
**155**	-5.0030	-0.0884	**155**	-4.9217	-0.0947
**150**	-5.0006	-0.0894	**150**	-4.9151	-0.0999
**145**	-4.9974	-0.0910	**145**	-4.9082	-0.1055
**140**	-4.9932	-0.0930	**140**	-4.9056	-0.1053
**135**	-4.9892	-0.0945	**135**	-4.8987	-0.1081
**130**	-4.9839	-0.0952	**130**	-4.8926	-0.1066
**125**	-4.9769	-0.0956	**125**	-4.8850	-0.1022
**120**	-4.9740	-0.0895	**123**	-4.8820	-0.0986
**115**	-4.9712	-0.0801	**120**	-3.0649	-0.0981
**110**	-4.9671	-0.0666	**117**	-3.0844	-0.1110
**105**	-4.9618	-0.0479	**115**	-3.0988	-0.1205
**100**	-4.9554	-0.0227	**110**	-3.1356	-0.1469
**99**	-4.9477	-0.0172	**105**	-3.1665	-0.1785
**98**	-4.9486	-0.0106	**100**	-3.1905	-0.2136
**97**	-3.3838	-0.0090	**95**	-3.1921	-0.2494
**96**	-3.3853	-0.0170	**90**	-3.1721	-0.2805
**95**	-3.3856	-0.0250	**85**	-3.1330	-0.3001
**90**	-3.3776	-0.0646	**80**	-3.0748	-0.3005
**85**	-3.3490	-0.0987			
**80**	-3.3016	-0.1215			

Table S22. Energies of the D$_1$ and D$_2$ states of ON-OCO as a function of θ_{NOC} for the intermolecular distances R_{NO}=2.4 Å. This data was calculated at the EOM-EA-CCSD/AVQZ//EOM-EA-CCSD/aug-cc-pVDZ level of theory. All energies are reported relative to a D$_2$-optimized geometry with an intermolecular distance of 20 Å. This data is plotted in Figure S20 in the Supporting Information.

θ_{NOC} (°)	R_{NO}=2.4 Å D$_1$ (eV)	R_{NO}=2.4 Å D$_2$ (eV)
**180**	-4.8115	-0.0098
**175**	-4.7689	-0.0601
**170**	-4.7597	-0.0795
**165**	-4.7582	-0.0849
**160**	-2.8127	-0.1333
**155**	-2.7752	-0.1566
**150**	-2.7470	-0.1840
**145**	-2.7281	-0.2135
**140**	-2.7212	-0.2436
**135**	-2.7246	-0.2733
**130**	-2.7414	-0.3015
**125**	-2.7639	-0.3278
**120**	-2.8051	-0.3531
**115**	-2.8455	-0.3776
**110**	-2.8866	-0.4024
**105**	-2.9225	-0.4296
**100**	-2.9359	-0.4580
**95**	-2.9245	-0.4831
**90**	-2.8852	-0.5001
**85**	-2.8137	-0.4971
**80**	-2.7208	-0.4620

Table S23 Energies of the D$_2$ states of NO-OCO as a function of θ_{OOC} for the intermolecular distances R_{OO}=2.7, 2.5, and 2.4 Å. This data was calculated at the EOM-EA-CCSD/AVQZ//EOM-EA-CCSD/aug-cc-pVDZ level of theory. All energies are reported relative to a D$_2$-optimized geometry with an intermolecular distance of 20 Å. This data is plotted in Figure S21 in the Supporting Information.

θ_{OOC} (°)	R_{OO}=2.7 Å D$_2$ (eV)	R_{OO}=2.5 Å D$_2$ (eV)	R_{OO}=2.4 Å D$_2$ (eV)

**180**	-0.0681	-0.0521	-0.0248
**175**	-0.0679	-0.0520	-0.0248
**170**	-0.0677	-0.0516	-0.0245
**165**	-0.0677	-0.0514	-0.0241
**160**	-0.0677	-0.0509	-0.0239
**155**	-0.0675	-0.0507	-0.0236
**150**	-0.0674	-0.0503	-0.0238
**145**	-0.0670	-0.0497	-0.0259
**140**	-0.0665	-0.0495	-0.0310
**135**	-0.0654	-0.0493	-0.0290
**130**	-0.0635	-0.0471	-0.0220
**125**	-0.0602	-0.0407	-0.0116
**120**	-0.0545	-0.0303	0.0032
**115**	-0.0457	-0.0150	0.0263
**110**	-0.0328	0.0074	-0.0211
**105**	-0.0150	0.0216	-0.0376
**100**	0.0089	-0.0024	-0.0613
**95**	0.0400	-0.0307	-0.0857
**90**	0.0942	-0.0558	-0.1038
**85**	0.1420	-0.0711	-0.1072
**80**	-0.0681	-0.0521	-0.0248

Table S24. Energies of the D_0, D_1, and D_2 states of ON-OCO as a function of R_{NO}. This data was calculated at the EOM-EA-CCSD/AVQZ//EOM-EA-CCSD/aug-cc-pVTZ level of theory. All energies are reported relative to a D_2-optimized geometry with an intermolecular distance of 20 Å. This data is plotted in Figure 10 in the paper.

R_{NO} (Å)	D_0 (eV)	D_1 (eV)	D_2 (eV)
**4**	-4.8911	-4.8911	-0.0307
**3.8**	-4.8903	-4.8903	-0.0375
**3.6**	-4.8889	-4.8889	-0.0462

**3.4**	-4.8863	-4.8863	-0.0571
**3.2**	-4.8807	-4.8807	-0.0698
**3**	-4.8681	-4.8681	-0.0826
**2.8**	-4.8401	-4.8401	-0.0898
**2.7**	-4.8127	-4.7764	-0.1035
**2.6**	-4.7811	-4.7242	-0.1069
**2.5**	-3.0509	-2.9057	-0.4337
**2.4**	-2.9531	-2.7728	-0.5494
**2.3**	-2.8352	-2.6067	-0.6696
**2.2**	-2.6932	-2.4009	-0.7888
**2.1**	-2.4652	-2.0886	-0.8992
**2**	-1.9317	-1.4854	-0.9984
**1.975**	-1.8576	-1.3752	-1.0269
**1.95**	-1.7703	-1.2518	-1.0567
**1.94**	-1.7368	-1.2038	-1.0682
**1.93**	-1.6742	-1.1208	-1.0796

Table S25. Energies of the D_0, D_1, and D_2 states of ON-OCO as a function of R_{NO}. This data was calculated at the EOM-EA-CCSD/AVQZ//EOM-EA-CCSD/aug-cc-pVTZ level of theory. All energies are reported relative to a D_2-optimized geometry with an intermolecular distance of 20 Å. These conformations have the O atom of NO oriented away from the CO_2. This data is plotted in Figure S23 in the Supporting Information.

R_{NO} (Å)	D_0 (eV)	D_1 (eV)	D_2 (eV)
**4**	-4.8911	-4.8911	-0.0307
**3.8**	-4.8903	-4.8903	-0.0375
**3.6**	-4.8889	-4.8889	-0.0462
**3.4**	-4.8863	-4.8863	-0.0571

**3.2**	-4.8807	-4.8807	-0.0698
**3**	-4.8681	-4.8681	-0.0826
**2.8**	-4.8401	-4.8401	-0.0898
**2.7**	-4.8127	-4.7764	-0.1035
**2.6**	-3.0495	-2.9675	-0.2068
**2.5**	-2.9739	-2.8523	-0.3483
**2.4**	-2.8706	-2.7066	-0.4980
**2.3**	-2.7455	-2.5337	-0.6514
**2.2**	-2.6038	-2.3400	-0.8001
**2.1**	-2.4755	-2.1572	-0.9264
**2**	-2.0786	-1.6428	-1.0372
**1.975**	-1.9776	-1.5042	-1.0710
**1.95**	-1.8869	-1.3761	-1.1045
**1.925**	-1.8130	-1.2672	-1.1368
